# Gene Expression Profiles of *Chlamydophila* *pneumoniae* during the Developmental Cycle and Iron Depletion–Mediated Persistence

**DOI:** 10.1371/journal.ppat.0030083

**Published:** 2007-06-22

**Authors:** André P Mäurer, Adrian Mehlitz, Hans J Mollenkopf, Thomas F Meyer

**Affiliations:** 1 Department of Molecular Biology, Max Planck Institute for Infection Biology, Berlin, Germany; 2 Microarray Core Facility, Max Planck Institute for Infection Biology, Berlin, Germany; University of California San Francisco, United States of America

## Abstract

The obligate intracellular, gram-negative bacterium *Chlamydophila pneumoniae (Cpn)* has impact as a human pathogen. Little is known about changes in the *Cpn* transcriptome during its biphasic developmental cycle (the acute infection) and persistence. The latter stage has been linked to chronic diseases. To analyze *Cpn* CWL029 gene expression, we designed a pathogen-specific oligo microarray and optimized the extraction method for pathogen RNA. Throughout the acute infection, ratio expression profiles for each gene were generated using 48 h post infection as a reference. Based on these profiles, significantly expressed genes were separated into 12 expression clusters using self-organizing map clustering and manual sorting into the “early”, “mid”, “late”, and “tardy” cluster classes. The latter two were differentiated because the “tardy” class showed steadily increasing expression at the end of the cycle. The transcriptome of the *Cpn* elementary body (EB) and published EB proteomics data were compared to the cluster profile of the acute infection. We found an intriguing association between “late” genes and genes coding for EB proteins, whereas “tardy” genes were mainly associated with genes coding for EB mRNA. It has been published that iron depletion leads to *Cpn* persistence. We compared the gene expression profiles during iron depletion–mediated persistence with the expression clusters of the acute infection. This led to the finding that establishment of iron depletion–mediated persistence is more likely a mid-cycle arrest in development rather than a completely distinct gene expression pattern. Here, we describe the *Cpn* transcriptome during the acute infection, differentiating “late” genes, which correlate to EB proteins, and “tardy” genes, which lead to EB mRNA. Expression profiles during iron mediated–persistence led us to propose the hypothesis that the transcriptomic “clock” is arrested during acute mid-cycle.

## Author Summary

*Chlamydophila (Chlamydia) pneumoniae (Cpn)* accounts for approximately one-tenth of the cases of community-acquired pneumonia worldwide, and persistent *Cpn* infections are thought to be associated with a variety of chronic diseases. Little is known about *Cpn* transcriptome changes during its biphasic developmental cycle (the acute infection) and persistence stages. Iron limitation, among several other treatments, has recently been shown to lead to persistent *Cpn* infection. How this pathogen reacts to iron-limiting host defense mechanisms is of great interest, as iron is an important factor affecting virulence. This article reports on the *Cpn* transcriptome during the developmental cycle and iron depletion–mediated persistence and reveals that genes coding for proteins of the infectious particle (the elementary body [EB]) were expressed constantly at the end of the cycle. In contrast, genes contributing to EB mRNA but not to EB protein showed an increasing expression at the end of the cycle. This suggested that most EB proteins are made in mid-cycle, and the redifferentiation process is initiated only by a limited number of genes. During iron depletion–mediated persistence, the *Cpn* transcriptome was altered in such a way that an arrest in *Cpn* gene expression can be proposed.

## Introduction

*Chlamydophila (Chlamydia) pneumoniae (Cpn)* is an obligate, intracellular gram-negative bacterium of the family Chlamydiaceae with a disposition for causing acute and persistent infections [1]. *Cpn* primarily infects the human respiratory tract, and accounts for about 10% of the cases of community-acquired pneumonia and 5% of bronchitis, sinusitis, and pharyngitis cases [2–4]. The pathogen is widely distributed, and up to 50% of the population of the developed world is seropositive by the age of 20 [5,6]. *Cpn* has been implicated in chronic inflammatory diseases, including reactive arthritis [7], asthma [8–11], chronic obstructive pulmonary disease [12], and atherosclerosis [13,14]. Consistent with this, respiratory infection in experimental animal models induces the formation of atherosclerotic lesions [15]. The acute infection of the Chlamydiaceae is characterized by a biphasic developmental cycle that alternates between metabolically dormant, infectious elementary bodies (EBs) and metabolically active, non-infectious reticulate bodies (RBs) [16]. After host cell entry, the EB is localized to an endosome, and the primary differentiation process is initiated. This process involves the commencement of bacterial metabolism and the conversion of the EB into the intracellular RB form. Bacterial reproduction takes place in a specialized vacuole termed inclusion, which separates the pathogen from the endocytotic pathway of the host cell. The pathogen modifies endosomal properties such that entry into the lysosomal pathway is prevented [17–19]. The RBs multiply by binary fission before differentiating back into EBs towards the end of the cycle. Finally, the EBs are released to infect neighboring cells.

Three gene expression patterns have been classified based on work done with *Chlamydia trachomatis (Ctr),* another member of the family Chlamydiaceae. These groups include genes for the early cycle (for initial events of the acute infection), mid cycle (for RB growth and division), and late cycle (for RB-to-EB differentiation) [20]. Besides going into the stage of the acute infection, it has been reported that Chlamydiaceae go into persistence. Persistence describes a long-term relationship between the bacteria and the infected host cell [21]. This state leads to an altered chlamydial growth characteristic, including the loss of infectivity and the appearance of inclusions containing fewer and altered particles referred to as aberrant bodies [16,22]. Several in vitro persistence models have been published, such as exposure to antibiotics [23,24] or interferon γ (IFN-γ) [25–29], or depletion of essential nutrients such as amino acids [30,31]. Iron starvation is also a common persistence model, and leads to abnormal growth and disruption of the developmental cycle in vitro [32–34]. Depletion of the intracellular iron pool is achieved in this model using the iron chelator deferoxamine-mesylate (DAM). The acute infection can be reconstituted by adding iron-loaded transferrin [32,34] or standard growth medium, leading to a release of the organisms from the persistent state. The iron depletion model is particularly important for understanding *Cpn* infections in vivo. The establishment of chronic *Cpn* infections in coronary arteries and heart disease has been shown to depend on the availability of iron [35–37]. Iron levels also fluctuate in endometrial tissues with changing estradiol levels [38], and thus contribute to the outcome of chlamydial infections. Moreover, iron is involved in many vital cellular functions [39]. Limiting iron availability is part of the host defense against bacterial infections [40,41], and may lead to *Cpn* persistence in vivo. Gene expression during the developmental cycle [42,43] as well as during IFN-γ treatment [44] has recently been described for *Ctr*. Gene expression has also recently been reported during three different models of persistence in *Chlamydia psittaci (Cps)* [45]. To date, expression of only a limited number of genes has been analyzed in *Cpn*. IFN-γ treatment leads to normal expression of genes required for DNA replication, the downregulation of genes related to cell division [46] and the upregulation of genes with functions in cell wall structure, glycolysis, and peptidoglycan synthesis [47]. The transcription of several genes of the type three secretion system (TTSS) is altered [48] during IFN-γ-induced persistence and in a continuous persistence model [49]. Genes encoding histone-like proteins, such as *hctA* and *hctB* [50,51], and components of the chlamydial membrane, such as *ompB* and *ompC,* have been shown to be expressed late during the developmental cycle and during the phase of redifferentiation [52]. Recently, a study showed that late *Cpn* genes remain upregulated in IFN-γ-mediated persistence [53].

Here, we present a detailed study of the *Cpn* transcriptome during the developmental cycle. We describe four different classes of genes, termed “early”, “mid”, “late”, and “tardy” genes. The latter is described as a fourth temporal group of genes, based on a transcriptional profile that is different from “late” genes. This study also describes the transcriptome of the *Cpn* EB. We have found a significant association between “late” gene clusters and genes coding for EB proteins, whereas “tardy” genes were mainly associated with genes coding for EB mRNA. The *Cpn* gene expression profiles provided a deeper insight into the complexity of *Cpn* transcriptional regulatory networks during the cycle of development. We also describe the altered transcriptional profile of *Cpn* during iron depletion–mediated persistence. The comparison of gene expression during persistence with the acute infection led to the finding that establishment of iron depletion–mediated persistence is more likely a mid-cycle arrest than a completely distinct gene expression pattern.

## Results

As microarray studies for obligate intracellular bacteria are still far from being routine procedures in most laboratories, special care was taken to optimize the procedures for RNA extraction, labeling, hybridization, and microarray design. Isolation of good quality RNA is an important precondition for obtaining accurate transcription profiles. *Cpn* mRNA represents only a minor proportion of the total RNA isolated from infected host cells. We developed a highly efficient method for RNA purification that led to an increase of *Cpn* mRNA from total RNA (Figure S1). The optimized purification protocol incorporates sonication of the chlamydial RB and the addition of glycogen as an RNA carrier during precipitation (Table S1). This optimized RNA purification protocol enabled us to use random priming instead of an organism-specific 3′ open reading frame primer set often used to amplify only the bacterial mRNA [42,43]. It has been shown that random priming is more accurate for the measurement of gene expression levels in bacteria [54]. Enrichment of *Cpn* using centrifugation was avoided, as this may induce a bacterial stress response leading to a biased gene expression signature. The method developed here constitutes an advancement compared to established protocols used for gene expression studies of Chlamydiaceae and other bacteria [42,43,55], and may present a promising alternative for future transcriptome studies of intracellular pathogens. An additional enrichment for prokaryotic mRNA was required only at 6 h and 12 h post infection (hpi) to increase the signal intensity on the microarray. For this purpose we used Ambion's MICROBEnrich kit. In order to ensure that this additional step had no influence on the relative gene expression pattern, the 6- and 12-hpi–enriched samples were compared with 48-hpi samples also enriched for chlamydial RNA to compensate for kit-specific bias. Additionally, comparison of samples from the same time points with and without enrichment showed no significant differences in their transcriptome profiles (Figure S2). At all other time points (18 to 72 hpi and the persistent stages), total RNA was processed without kit-specific enrichment. RNA quality and quantity was monitored using the Agilent Bioanalyzer 2100 (Figure S3). A 50-mer oligo microarray was designed to cover all open reading frames of the *Cpn* strains CWL029, AR39, and J138. Each oligo was checked for homology to the human genome using the BLAST algorithm to ensure little to no cross-hybridization with host RNA. The amount of cross-hybridization with host RNA was also evaluated experimentally. Total RNA was isolated using the same procedure from non-infected HEp-2 cells, and hybridized to the microarray using the same conditions as for RNA from infected cells. No hybridization signals were detected using host RNA (unpublished data). The occurrence of acute and persistent infections was monitored by confocal microscopy with immunofluorescence staining of *Cpn* (Figure S4). In addition, the establishment of the iron depletion–mediated persistent infection was verified using an infectivity assay [32]. All persistent infections showed markedly reduced numbers of progeny (Figure S5). Differences in RNA preparation, labeling, hybridization, and bacterial multiplication during the developmental cycle were compensated using LOWESS normalization of the signals from both channels on each array. Statistical analysis with Significance Analysis of Microarrays (SAM) software [56] revealed the lowest false discovery rate (FDR), and therefore, the least variation at 48 hpi (unpublished data). Most *Cpn* genes were expressed at the cycle midpoint (unpublished data), and EB progeny were not present at this time point (Figure S6). These data indicated that 48 hpi was suitable as a common reference time point. Interestingly, hybridization of genomic DNA as a common reference revealed a higher variance in measured gene expression values (unpublished data). Transcriptional changes throughout the *Cpn* developmental cycle were defined by comparison of expression levels at various time points to 48 hpi. The experimental setup is consistent with a recent publication for C. trachomatis [43]. Complete synchronization of the acute infection, especially at later stages, is not possible. For this reason, appropriate statistical analysis is important for studying *Cpn* gene expression. Statistical analysis was performed using SAM software on the raw data values. Expression profiles of genes that SAM analysis detected not to be robust enough to be distinguished were omitted from further data analysis. These included situations where gene expression was possibly coming from a minor population of bacteria, and occurred especially towards the end of the cycle. To our knowledge, this is the first report to apply significance analysis to *Cpn* gene expression analysis. Usage of 48 hpi as a common reference time point in combination with LOWESS normalization and SAM analysis resulted in data which were highly consistent with the results of C. trachomatis microarray studies [42,43] (unpublished data).

### The *Cpn* Transcriptome in the Acute Infection Can Be Separated into Four Classes Based on the Gene Expression Profiles

**Figure 1:**
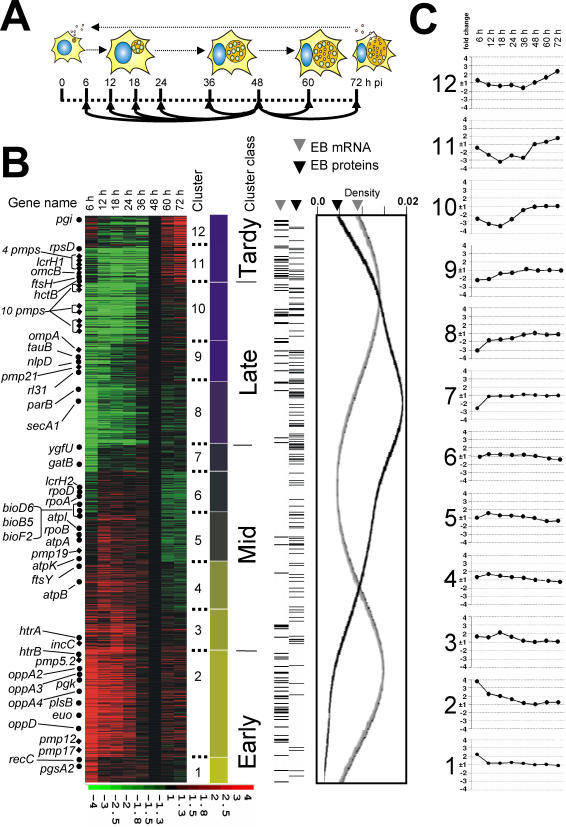
Analysis of *Cpn* Gene Expression in the Acute Infection (A) Cycle of development, time scale, and experimental design. Cells were infected and RNA extracted at the indicated time points, and 48 hpi was used as a reference time point. The arrows indicate the direction of the cycle of development. (B) Between 6 and 72 hpi, 754 significant expression profiles were determined using ratio profiles with SAM and SAMSTER and analyzed using SOM clustering (YDim = 12). Gene Cluster software using a one-class time-course analysis was carried out and plotted with TreeView software. Gene expression is displayed in fold change represented by the color bar beneath the figure with the expression at 48 hpi set to ±1. Names of genes mentioned in the text are depicted on the left, and cluster numbers, a color representation for the clusters, and cluster classes are depicted on the right of the cluster profile. Gene identification numbers, gene definitions, and gene names can also be viewed at http://www.ncbi.nlm.nih.gov. The cluster profile positions of 91 genes coding for mRNA (EB mRNA) and 127 genes coding for proteins present in the *Cpn* EB [63] (EB proteins) are displayed on the right of the cluster profile. A Gaussian Kernel Density Estimation for the EB mRNA and EB proteins positions in the cluster profile is represented by the graph on the right. Expanded annotated details for the gene lists and calculations are provided in Figures S8 and S9 and Tables S5 and S6. (C) Mean expression values of genes located in the defined clusters. For expanded annotated details, see Table S3A.

Statistical analysis of the time course identified 754 of 1,062 genes as significantly expressed (Table S2), and 533 genes met a 1.8-fold cutoff criterion (Table S3). *Ctr* and *Cpn* gene expression are primarily characterized relative to the onset of gene expression in the literature. Instead, we used the gene expression profiles to define clusters and classes of genes active at the same time. Using self-organizing map (SOM) cluster analysis, 12 clusters of gene expression profiles were defined for the developmental cycle (Figure 1B). These 12 clusters were further manually grouped into four main (cluster) classes based on their expression profiles; these cluster classes were termed “early”, “mid”, “late”, and “tardy”. The terminology for the former three classes is based on gene expression publications for C. trachomatis [42,43], whereas the “tardy” class is a novel definition.

Genes of the “early” cluster class were those expressed at high levels in the initial stages (6 hpi) of the infection and at constitutive expression levels later in the cycle. This included clusters 1 and 2. Cluster 1 expression dropped to its minimum at 12 hpi, whereas the expression minimum of cluster 2 was reached at 36 hpi. None of these clusters showed significant differential regulation at later time points in the cycle. This difference in the expression profiles suggests a function for cluster 1 as immediate early genes in the cycle, whereas genes from cluster 2 are expressed up to the middle of the cycle. The “early” cluster class includes *euo,* considered to be a classical early gene [57–59]. The “mid” gene cluster class is represented by genes transcriptionally activated following the initial stages of the infection. Cluster 3 showed an expression peak at 18 hpi, and clusters 4 and 5 at 18 hpi. The expression profiles of clusters 4 and 5 were similar. Cluster 6 consisted of genes expressed at similar levels throughout the measured time points of the developmental cycle. They were manually grouped into the “mid” cluster class, however, it can not be ruled out that some or all of the genes might show an increased expression at a time point not measured (e.g., <6 hpi). Expression of cluster 7 began at 12 hpi, and continued at a constant level throughout the rest of the cycle. The “late” gene cluster class includes clusters 8 to 10. Genes of these clusters became transcriptionally active after 36 hpi, and maintained constant expression until the end of the cycle. The genes *hctA* [60] and *parB* [61] are described in the literature as classic examples of late genes; these genes are also included in the “late” class described here.

The definition of the “tardy” gene cluster class is a novel category comprising clusters 11 and 12. In contrast to expression clusters of other “late” genes, “tardy” genes were differentially expressed at 60 and 72 hpi. The expression levels for “late” and “tardy” genes also were significantly different at 72 hpi using quantitative real-time PCR (qRT-PCR), verifying the microarray results. The possibility of the “tardy” gene cluster being due to reinfection was ruled out by the following observations: First, the bulk of EB progeny was released from the host cell subsequent to 72 hpi (Figure S6). Even if a low level of reinfection occurred, it would be negligible compared to the high amount of the non-released EBs present at later time points. Second, reinfection would have also influenced the gene expression of genes belonging to “early” clusters. Yet, we observed constant expression of the “early” cluster 1 genes throughout the end of the cycle (Figure 1C), showing no evidence of reinfection. Thus, we conclude that the observed increase in gene expression of clusters 11 and 12 at later stages of the cycle did not result from reinfection at these time points.

### EB mRNA Transcripts Are Mainly Connected with “Tardy” Clusters, whereas Genes Coding for EB Proteins Are Mainly Connected with “Late” Clusters

EBs were separated from RBs and cell debris to investigate the mRNA content of EBs. The purity of the preparation was verified by electron microscopy prior to and after the purification (Figure S7). Subsequently, mRNA was extracted, labeled, and hybridized as described. Two self–self hybridizations were performed. All signal intensities below 500 were considered background noise and were not included in the analysis. After this cutoff, transcripts for 91 genes were present within the EBs (Table S4). Using Gaussian Kernel Density Estimation (http://www.wessa.net) and LACK software [62], the EB-specific mRNA transcripts were compared to the cluster classification (Figures 1B and S8; Table S5). Transcripts present in the *Cpn* EBs could be significantly linked to certain “early” and “tardy” clusters. In contrast to “early” cluster 1 or “mid” clusters, the “early” cluster 2 (*n* = 33, *p* < 0.01) contained a significant number of EB transcripts, indicating these are transcriptionally active until the end of the cycle. Of the “late” class, cluster 10 contributed most, but not significantly, to EB-specific transcripts (*n* = 14, *p* = 0.09), whereas the “tardy” clusters 11 (*n* = 11, *p* = 0.02) and 12 (*n* = 9, *p* = 0.05) were significantly linked to EB-specific transcripts. Of the clusters significantly contributing to EB mRNA content, the “early” cluster 2 showed no constantly increasing expression between 60 and 72 hpi, whereas the “tardy” clusters 11 and 12 did (Figure 1C). These data support that increasing expression levels of the “tardy” clusters did not result from accumulating EB towards the end of the cycle (Figure S6), and strengthen the definition of the “tardy” class as a separate class. Transcriptionally inactive EBs most likely represent a snapshot of the last transcriptionally active phase before redifferentiation, and predominantly include transcripts from the “early” cluster 2 and both “tardy” clusters. It has been discussed that genes showing maximum expression towards the end of the cycle may play a primary role in events such as RB-to-EB redifferentiation and the synthesis of EB-specific proteins [20]. To investigate whether genes of the “tardy” class contribute to the establishment of EB proteins, our expression data were compared with data from a proteomic study targeting the EB protein composition [63] using Gaussian Kernel Density Estimation and LACK software [62] (Figures 1B and S9; Table S6). Of the genes coding for EB proteins, 28 were linked to cluster 8 (*n* = 28, *p* < 0.01) and 15 to cluster 9 (*n* = 15, *p* < 0.05). For clusters 10 and 11, 16 (*n* = 16, *p* = 0.27) and ten genes (*n* = 10, *p* = 0.28) were linked to EB-specific proteins, respectively. Only four genes were linked to EB-specific proteins in cluster 12 (*n* = 4, *p* = 0.94). The majority of genes coding for EB proteins accumulated in clusters 8 and 9. Cluster 10 of the “late” class and clusters 11 and 12 of the “tardy” class contained only a minority of genes coding for EB proteins. However, the association of EB-specific mRNA transcripts to cluster 10 and of EB proteins to cluster 11 was not significant. Cluster 11 included genes coding for several membrane proteins (e.g., *omcB, pmp*s), proteins for energy metabolism (e.g., *sucC, sucD*), and several hypothetical proteins. The data comparison revealed that genes coding for EB proteins were primarily connected with “late” clusters, whereas genes coding for EB mRNAs were mainly connected with “tardy” clusters.

### Predominant Changes in the Transcriptional Pattern of the Acute Infection

**Figure 2:**
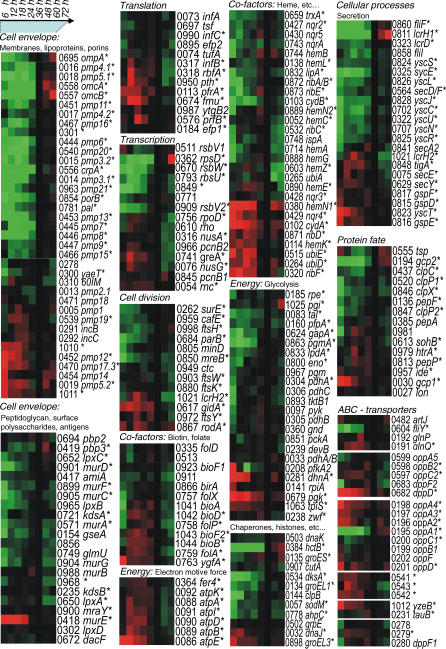
Functional Grouping and Cluster Analysis Detailed expression profiles of functionally related sets of genes. Clusters were created using SOM cluster analysis using the color scale as described for Figure 1. Genes that were calculated as significantly regulated using SAM 2.0 are indicated with an asterisk. For a more detailed view, see Table S3A. Gene identification numbers, gene definitions, and gene names can be viewed at http://www.ncbi.nlm.nih.gov.

**Figure 3:**
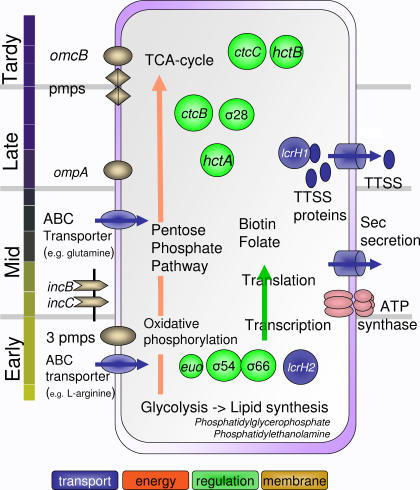
Schematic Overview of Important Cellular Functions Correlated with the Expression Profiles and Cluster Classes The illustration presents an overview of a C. pneumoniae cell, and the onset of gene expression for genes with important cellular functions according to their cluster classes. Gene identification numbers, gene definitions, and gene names can be viewed at http://www.ncbi.nlm.nih.gov.

During the cycle of development, *Cpn* undergoes morphological differentiation, reflected in expression changes of groups of functionally related genes (Figures 2 and 3). Of the family of polymorphic membrane proteins (Pmps) [64], *pmp5.2 (Cpn0019), pmp12 (Cpn0452),* and *pmp17.3 (Cpn0470)* were expressed early, whereas *pmp19 (Cpn0539)* [65] was grouped into a “mid” cluster. The majority of pmp genes, including the autotransporters *pmp6, pmp20,* and *pmp21* [66,67], were not expressed before 48 hpi and were located in the “late” cluster 10 and the “tardy” cluster 11. The expression of *incC,* associated with the chlamydial inclusion [18], and *ftsY* and *ftsW,* associated with cell division [68,69], peaked at mid-cycle. The essential inner membrane-bound protease *ftsH* belonged to the “tardy” class, suggesting a role mainly in RB-to-EB conversion. Both *ompA* and *omcB* were expressed toward the end of the cycle. Genes for peptidoglycan synthesis showed time-specific expression, with *murE* in the “early” class and *murA, murC, murD,* and *murF* in the “late” class.

**Figure 4:**
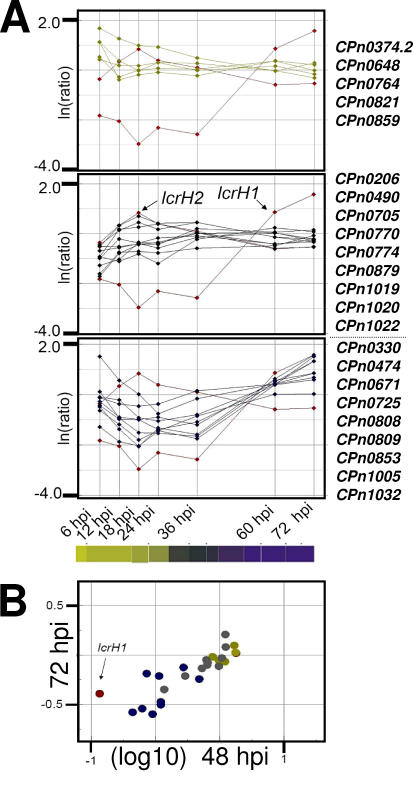
Expression Profiles of Genes Coding for Predicted Putative Factors The expression profiles of 24 genes coding for proteins predicted to be type three secreted, [74] as well as the genes coding for the TTSS chaperones LcrH1 and LcrH2, are displayed. (A) According to the expression profiles in the acute developmental cycle, the genes can be grouped into “early”, “mid”, and “tardy” genes. “Mid” gene expression correlated with *lcrH2,* whereas “tardy” gene expression correlated with *lcrH1*. This indicates time-dependent functions throughout the cycle. Data are represented as ln(ratio) (*y*-axis) versus time (*x*-axis). (B) Comparison plot showing the expression of these 24 genes coding for the predicted TTSS proteins in the iron depletion–mediated persistence. The *x*-axis represents 48 hpi and the *y*-axis 72 hpi of the persistent infection. Mainly “tardy” genes are downregulated. Data are displayed as log10. Gene identification numbers, gene definitions, and gene names can be viewed at http://www.ncbi.nlm.nih.gov.

Protein secretion plays a key role for host–pathogen interactions [70]. The microarray expression data for genes encoding the TTSS were highly consistent with a recent report [48]. Noteworthy is the expression of two TTSS chaperones, *lcrH2* in the “mid” class and *lcrH1* in the “tardy” class. Genes encoding TTSS proteins recently predicted in the *Cpn* genome [71–74] were expressed in “early”, “mid”, and “tardy” expression profile clusters (Figure 4). Expression of “mid” class TTSS genes correlated with *lcrH2,* whereas “tardy” class TTSS genes correlated with *lcrH1.* Genes coding for the type two secretion system (TIISS) were grouped entirely into the “mid” class. The expression of genes encoding the transmembrane and ATP-binding proteins of predicted transporter homologs ([75]; http://www.genome.ad.jp/kegg) peaked early during the cycle of development. Genes for substrate-binding proteins had variable profiles. For instance, the L-arginine-binding protein *artJ* was an “early” gene, whereas *fliY,* a predicted glutamine-binding protein, was a “late” gene (Figure 2). Genes involved in metabolism transport, such as *oppA2, oppA3, oppA4,* and *oppD,* were expressed early in the developmental cycle. The fructose bisphosphate aldolase *(Cpn0281)* and *pgk (Cpn0679)* genes involved in glycolysis were strongly expressed at initial stages of the cycle (6 hpi). Other genes involved in energy metabolism were expressed in both “early” and “mid” clusters. The *pgsA1 (Cpn0615)* gene was expressed early, and *tpiS (Cpn1063),* involved in the synthesis of glycerone phosphate, as well as *plsB (Cpn0958), plsC (Cpn0569), pssA (Cpn0983),* and *psdD (Cpn0839),* exhibited “mid” expression patterns. These genes are part of the pathway synthesizing phosphatidylglycerophosphate (PGP) and phosphatidylethanolamine (PE). Both PGP and PE are essential membrane components [76].

Genes coding for the synthesis of cofactors such as biotin and folate exhibited a mid-cycle expression profile. The genes *sdhB* and *sdhC,* coding for the iron-sulfur subunit of succinate dehydrogenase and the apocytochrome b558, respectively, were both upregulated early*.* Expression of genes encoding proteins of the ATP-synthase complex peaked between 6 and 24 hpi, with 4-fold upregulation of *atpE* at 6 hpi and 2-fold upregulation of *atpA, atpB, atpE,* and *atpI* at 12 hpi. The genes *gapA (Cpn0624), pgmA (Cpn0863),* and *eno (CPn0800)* were expressed at subsequent stages of the cycle. Their gene products are part of the pathway synthesizing phosphoenolpyruvate, which is fed into the citrate cycle. Malate dehydrogenase *(Cpn01028), fumC (Cpn1013), sucC (Cpn0973), sucB1 (Cpn0377),* and *lpdA (Cpn0833)* are expressed in the “late” class. Genes involved with the pentose phosphate pathway, including *zwf* (*Cpn0238* [cluster 5]), *rpe* (*Cpn0185* [cluster 7]), *and tal* (*Cpn0083* [cluster 8]), were expressed in both “mid” and “late” classes. The gene for glucose-6-phosphate isomerase, *pgi (Cpn1025),* a dimeric enzyme catalyzing the reversible isomerization of glucose-6-phosphate to fructose-6-phosphate, was a “tardy” gene.

Genes coding for DNA metabolism *(recF, recC, nth, xerC)* were members of the “early” class, whereas genes coding for transcription and translation (numerous *rs* and *rl* genes encoding ribosomal proteins) belong to the “mid” class. Transcriptional control is mediated via the alpha *(rpoA)* and beta *(rpoB)* subunits of the RNA polymerase [64,73], which were “mid” class genes, as well as the major sigma factor *rpoD/σ^66^/σ^B^* and the alternative factors *rpsD/σ^28^/σ^F^* and *rpoN/σ^54^/σ^N^*. The latter two have been discussed to be responsible for regulating “mid” and “late” genes [77–81]. The major sigma factor was constantly expressed until 72 hpi, where it decreased by 1.8-fold. The *rpoN* alternative sigma factor was expressed at a constant level throughout the cycle. The expression of the second alternative sigma factor, *rpsD,* increased more than 2-fold at the end of the cycle.

Taken together, these results suggest that at initial stages of the cycle, gene expression reflected the pathogen's need for energy and transport of metabolites, as well as de novo synthesis of cellular components such as phospholipids. The transcription of genes belonging to the “mid” cluster class paves the way for an altered energy metabolism and beginning of *Cpn* replication and cell division, as well as growth of the chlamydial inclusion. At later stages, Chlamydiaceae depend on the acquisition of host components such as 2-oxoglutarate [82]. Genes coding for inclusion membrane and cell division were expressed mid-cycle, whereas the majority of genes coding for Pmps and peptidoglycan synthesis were expressed at the end of the cycle. This may reflect the importance of the latter during RB–EB redifferentiation. The high correlation of the expression profiles for genes encoding the putative TTSS proteins and the two chaperones *lcrH1* and *lcrH2* suggest that TTSS genes might be connected to certain chaperones. Further, the appearance of genes encoding TTSS proteins in three expression profile clusters indicated different roles during the infection. The data suggest an ordered gene expression for the morphological and functional events occurring during the *Cpn* developmental cycle.

### Transcriptional Changes Suggest That Iron-Mediated Persistence Is an Arrest in the Transcription Profile

**Figure 5:**
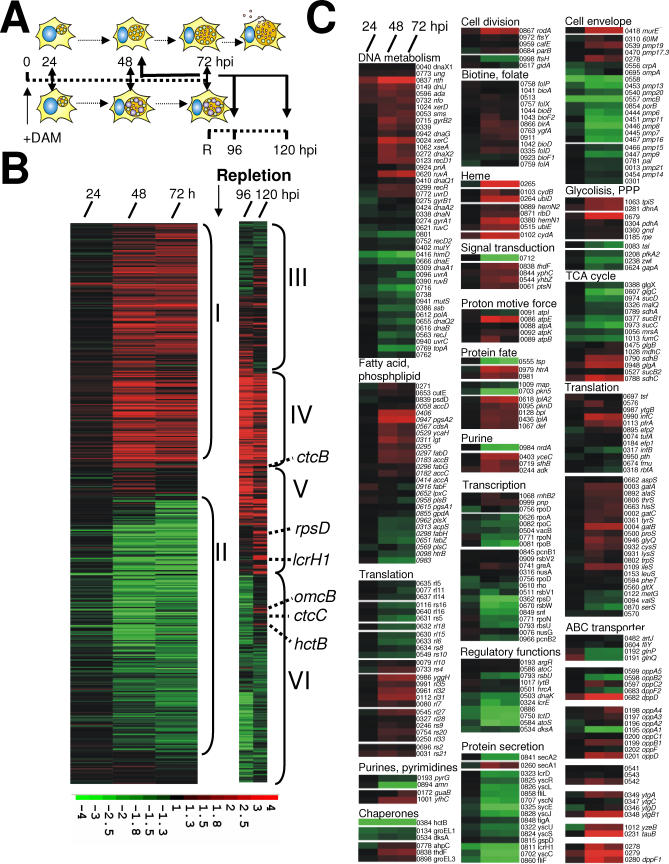
Differentially Regulated Genes during DAM-Mediated Iron-Depleted Persistent Infection (A) Experimental design. Cells were infected with *Chlamydia* at time point 0, incubated with DAM at 2 hpi to obtain the persistent infection (+DAM), and lysed at 24, 48, and 72 hpi. Iron-depleted cells were repleted at 72 hpi (R) and harvested at 96 h and 120 hpi. Iron-depleted samples were compared with the corresponding time points of the acute infection. Iron-repleted time points were compared with 48 hpi of the acute infection (indicated by arrows). (B) Clustered expression profiles of 461 significantly expressed genes calculated using SAM. Gene expression is displayed in fold change represented by the color bar beneath the figure. (C) Functional grouping and detailed expression profiles of genes differentially expressed during persistent infection are displayed. For expanded annotated details, see Table S3B. Gene identification numbers, gene definitions, and gene names can be viewed at http://www.ncbi.nlm.nih.gov.

It has previously been shown that Chlamydiaceae have an altered gene expression pattern during persistence [49]. To analyze the *Cpn* transcriptome during iron depletion–mediated persistence, *Cpn*-infected cells were treated with DAM. Iron-starved, persistent *Cpn* showed an altered morphological appearance (Figure S4) and a loss of infectious progeny (Figure S5). Total RNA extracted from DAM-treated samples taken at 24, 48, and 72 hpi was compared to the corresponding time points during acute infection. For iron repletion experiments, DAM-containing medium was replaced at 72 hpi with normal growth medium. Samples were taken 24 and 48 h after the repletion time point and compared to 48 hpi of the acute infection (Figure 5A). Statistical analysis using SAM 2.0 identified a total of 461 significantly regulated genes during DAM-mediated persistence (Table S7A–S7C). Clustering of the significantly regulated genes using a SOM algorithm displayed two main directions of regulation (Figure 5B). At 24 hpi, only eight genes showed differential expression; at 48 hpi, 98 genes were up and 82 downregulated; and at 72 hpi, 25 genes were up and 33 genes downregulated. In summary, 123 genes were downregulated and 124 genes were upregulated more than 1.8-fold between 24 and 72 hpi (Table S3B). Of the 754 genes significant in acute infection, 97 were significantly upregulated and 104 were significantly downregulated during persistence (Table S3A).

**Figure 6:**
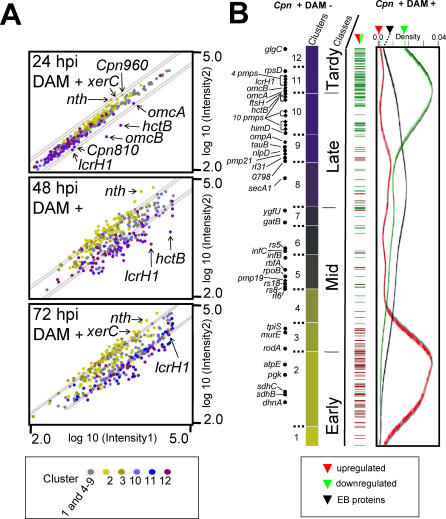
Comparison of *Cpn* Expression during Persistence with the Cluster and Cluster Class Categorization from the Acute Infection Only genes with significant expression changes during both persistent and acute infections are displayed. (A) Intensity blots for 24, 48, and 72 hpi of the persistent infection. Expression values are compared to the corresponding time points of the acute infection. Genes of the persistent infection that can be linked to the acute clusters 2, 3, 10, 11, or 12 are highlighted. Data are displayed as log10 values of the raw intensities. The blue and the red lines indicate a 1.8-fold and a 2.0-fold cutoff, respectively. (B) The 201 significantly and differentially (>±1.8-fold) regulated genes in the persistent infection were compared to their cluster categorization during acute infection (see Figure 1). The bar (DAM +) displays the expression tendency of these genes during persistence, with green for downregulation and red for upregulation, and the position of the gene in the acute cluster categorization from Figure 1. Names of genes mentioned in the text are depicted on the left. For genes upregulated and downregulated during persistence, a Gaussian Kernel Density Estimation representing the positions in the cluster profile is displayed on the right. Gene identification numbers, gene definitions, and gene names can be viewed at http://www.ncbi.nlm.nih.gov.

The nature of the establishment of persistent infections is still a matter of debate [83]. We examined which gene expression clusters of the acute infection were most affected during iron depletion–mediated persistence. Genes having no significant expression differences or showing no common direction of expression change during persistence were omitted from this analysis. Figure 6 shows graphical representations of the 201 genes with significantly altered expression during persistence in relation to the cluster profile of these genes during acute infection. At 24 hpi, only a few genes were downregulated (Figure 6A), including *omcA, omcB, hctB,* and *lcrH1,* which are all markers for RB-to-EB differentiation. The *Cpn* expression profile was altered at 48 and 72 hpi after induction of persistence. We used the LACK software and Density Kernel Estimation to analyze the distribution of genes affected by iron-mediated persistence (Figure 6B). Of the 97 genes significantly upregulated during the persistent infection, 54 were linked to cluster 2 (*n* = 54; *p* < 0.01) and 17 to cluster 3 (*n* = 17; *p* < 0.01) of the acute cycle (Figure S10; Table S8). During persistence, 104 genes were significantly downregulated, including 24 linked to cluster 10 (*n* = 24; *p* < 0.01), 31 to cluster 11 (*n* = 31; *p* < 0.01), and 19 to cluster 12 (*n* = 19; *p*<0.01) of the acute infection. Cluster 8 was slightly but not significantly affected, with downregulation of 12 genes (*n* = 12; *p* = 0.53) (Figure S11; Table S9).

A minority of genes with altered expression during persistence were located in other acute clusters (1 and 4–9; Figures S10 and S11). These included genes involved in various processes. Expression of the *rs5, rl6, rs8, rl18, rpoB, rbfA, infB,* and *gyrA* genes was altered. These are involved in transcription, translation, and DNA metabolism. Genes involved in metabolism and transport, such as *nrdA, nrdB, arcD, secY, yscN,* and *yscL,* were downregulated during persistence. Genes coding for membrane proteins *(pmp19* and *nlpD),* involved in transport *(secA1* and *tauB),* and factors for translation *(infC* and *rl31),* respectively, were upregulated during persistence*.* The expression of these genes during persistence most closely resembled their expression at 36 hpi in the acute cycle. During persistence, primarily genes in acute clusters 2 and 3 were upregulated, whereas mainly genes in acute clusters 10, 11, and 12 were downregulated. This finding supports the idea that iron depletion–mediated persistence is an arrest during the “mid” cycle of development, rather than being a new transcriptional profile. It remains open whether the transcriptional profile observed during DAM-mediated persistence results from iron depletion in the host cell, leading to a loss of the signal for switching off “early” and switching on “late” and “tardy” genes, or a reduced iron level inside the pathogen itself.

### Predominant Changes in the Transcriptional Pattern of the Persistent Infection

Of the genes coding for Pmps, only *pmp19* was upregulated during persistent infection, whereas 11 other pmp genes were downregulated. The *omcA* and *omcB* genes were also downregulated. Of the genes involved in peptidoglycan synthesis, *murB* was downregulated and *murE* was upregulated. The rod-shape determining protein *rodA,* which is involved in cell division, was upregulated 2-fold. This gene has been speculated to be responsible for abnormal chlamydial forms during persistent infection [84]. Of the other genes involved in cell division, only *ftsH* was downregulated, and expression of the remaining genes was unaltered.

Genes coding for protein secretion were also affected during persistent infection. Genes responsible for the TTSS were downregulated. In fact, expression of the *lcrH1* chaperone was 9-fold lower than during acute infection. Of the 24 predicted putative TTSS proteins [74] that have been shown to fit into “early”, “mid”, and “tardy” clusters in the acute infection, those belonging to “tardy” clusters were affected during persistent infection, whereas expression of the others remained unchanged (Figure 4B). Genes coding for the TIISS were also downregulated during persistence, the only exception being *secA1,* which was upregulated.

During persistence, 13 genes involved in energy metabolism were differentially expressed. The *atpE* ATP synthase subunit was increased up to 3-fold at 48 hpi. Three genes involved in glycolysis *(tpiS, pkg,* and *dhnA),* two genes of the TCA cycle *(sdhB* and *sdhC),* and the *glgA* gene involved in starch and sucrose metabolism were also upregulated. The *zwf* gene of the pentose phosphate pathway and two genes of starch and sucrose metabolism *(glgC* and *glgP)* were downregulated during persistence.

It is not yet clear how iron depletion mediates changes of gene expression. Expression of the DNA-binding proteins *himD,* a histone-like DNA-binding stress response protein, and *hctB,* a histone-like protein, were reduced, whereas *hctA* expression was unaltered. The expression of *σ28* was downregulated 2-fold during persistence, while *σ54* expression remained unchanged. Taken together, these results demonstrate that *Cpn* altered the expression of genes coding for membrane proteins, secretion processes, energy metabolism, and key genes for the transcriptional control during the persistent state.

### Transcriptome Signatures of Repleted *Cpn*

Persistent *Cpn* were returned to the acute infection state, replacing the DAM-containing media with normal media. Gene expression changes were monitored during the recovery phase. Reactivation of *Cpn* was accompanied by an upregulation of genes belonging to the “tardy” class, including *rpsD, omcB, lcrH1, hctB,* and *atoS* (group V, Figure 5B). Some genes upregulated during persistence were also upregulated during iron repletion (group IV, Figure 5B). However, after iron repletion, the majority of genes upregulated during persistence returned to expression levels similar to those in the “tardy” stages of the acute developmental cycle (group III, Figure 5B). Group VI includes genes downregulated during persistence, which are upregulated again after iron repletion. In summary, iron repletion leads to a coordinated continuation of the developmental cycle.

### qRT-PCR Verification, geNorm, and Housekeeping Index

**Figure 7:**
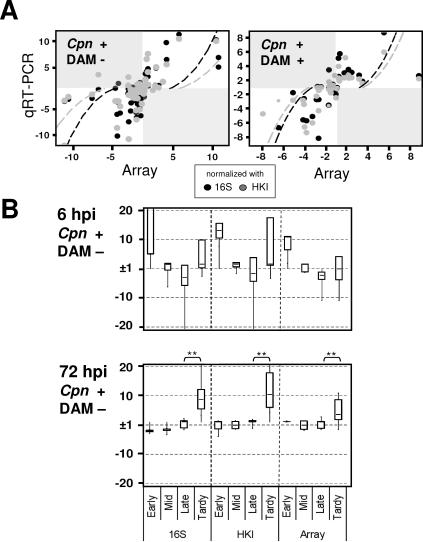
qRT-PCR Verification of Microarray Data Genes were picked from different cluster classes shown in Figure 1 (see Table S12 for expression data), with a focus on “late” and “tardy” genes. (A) Correlation between the fold changes for the acute infection (6, 12, and 72 hpi) and the iron depletion–mediated persistence (24, 48, and 72 hpi) between microarray data and qRT-PCR data normalized using either 16S rRNA (white dots) or the HKI (solid dots). For most genes, the qRT-PCR data correlated well with the array data, whereas a few genes were slightly anticorrelated. The data approximately follow X̂3 curves (dashed lines), indicating that expression reported by qRT-PCR was consistantly higher than expression reported by the microarray. (B) Box-plot analysis of genes for the “early”, “mid”, “late”, and “tardy” clusters at 6 hpi and 72 hpi. The qRT-PCR data were normalized using either 16S rRNA or the HKI as above. Expression levels of the “late” and “tardy” genes were calculated to be significantly different at 72 hpi using the Student *t*-test, verifying the microarray results.

Normalization of microarrays and qRT-PCR is based on different premises. Whereas microarray data ideally measures the expression of all genes in the genome simultaneously, qRT-PCR measures only a single gene. Therefore, normalization against an internal standard is required for qRT-PCR. Housekeeping genes that exhibit minimal variation of expression within the experimental setting are chosen as the internal standard. Housekeeping gene expression, although often constant in a given cell type or experimental condition, may show significant variation under other conditions [85–87]. Total RNA includes a large amount of rRNA molecules, yet this does not warrant their suitability as control genes for gene expression analysis [88]. Transcription of rRNA may be subject to variation by biological factors and drugs [89–91]. As no published data were available prior to our study about the suitability of 16S rRNA as a housekeeping gene for qRT-PCR analysis of *Cpn* gene expression, its variation throughout the measured time points was compared with other housekeeping candidates. Ten genes were chosen from the 100 least regulated genes based on the microarray results of the acute and persistent infection (Table S11). Genes belonging to different functional classes were chosen in order to reduce the risk that genes involved in the same function might be co-regulated. A gene stability measurement was carried out relying on the principle that the expression ratio of two ideal internal control genes is identical in all samples, regardless of the experimental condition or cell type [92]. Real-time PCR was performed on samples from the acute infection and DAM-mediated persistence, and these houskeeping candidates were ranked according to their expression stability using the geNorm software (http://medgen.ugent.be/~jvdesomp/genorm) (Table S11). The M-value is the gene expression stability parameter as calculated by geNorm; the lower the M-value, the more stably expressed is the reference gene. 16S rRNA has been used in a variety of published studies to normalize chlamydial gene expression [42]. However, 16S rRNA was not among the best three candidates for housekeeping genes. Subsequently, a housekeeping index (HKI) was defined with the geometric mean of the three most stably expressed housekeeping candidates, *abcT, l29,* and *tufA.* In addition, 16S rRNA was also used for normalization. To our knowledge, this is the first report applying such an approach for expression studies of an intracellular bacterium. Equivalent to the microarray experiments, the 48 hpi time point was used as the reference for the acute infection. To verify the microarray results, qRT-PCR experiments were performed for selected genes from different regulated cluster classes. Emphasis was placed on genes of the “late” and the “tardy” classes (Table S10) to verify the different expression profiles of these two classes toward the end of the cycle. Relative expression was calculated using the efficiency corrected ΔΔCT model [93]. Experiments were conducted in at least three technical and two biological replicates. The qRT-PCR data were normalized using either 16S rRNA or the housekeeping index (HKI). Comparisons of gene expression during acute and persistent infection measured using qRT-PCR and microarrays are presented in Figure 7A. Normalization to 16S rRNA produced higher standard deviation and scattering as compared to normalization using the HKI (Table S12). We verified our results from microarray analysis via qRT-PCR, regardless of the normalization method (either 16S rRNA or the HKI). Expression measured using qRT-PCR correlated well with expression measured using the microarray for most genes, with a limited number being anticorrelated (Figure 7A). Microarray analysis tended to underestimate expression changes in comparison to analysis using qRT-PCR. Based on these results, a lower fold change detected by microarray analysis could be considered relevant, which led to the introduction of the 1.8-fold cutoff for microarray data. We were interested to reproduce the different expression profiles observed for “late” and “tardy” genes at initial (6 hpi) and late time (72 hpi) points using qRT-PCR. qRT-PCR showed expression differences between initial and late time points for the four cluster classes (Figure 7B). At 6 hpi, “early” and “tardy” genes (of cluster 12) showed increased expression as opposed to “late” genes. At 72 hpi, only “tardy” gene (clusters 11 and 12) expression was increased, compared to all other clusters. The expression difference between “late” and “tardy” genes was calculated to be significant (*p* < 0.01) using the Student's *t*-test. The qRT-PCR experiments verified the results obtained using microarray technology, as well as the expression differences for “late” and “tardy” genes at 6 hpi and 72 hpi.

## Discussion

The microarray analysis of *Cpn* presented here was performed to identify transcriptional changes at various stages of the developmental cycle, as well as during *Cpn* persistence induced by iron depletion. Further, the mRNA profile of the infectious EBs was analyzed. Whereas existing literature mainly use the onset of transcription for gene classification [42,43], this study used gene expression profiles to characterize the *Cpn* transcriptome throughout the developmental cycle. Twelve gene expression clusters were defined using SOM clustering, and four transcriptional classes were distinguished manually and termed as “early”, “mid”, “late”, and “tardy”. The terminology of the former three classes has been used since 2000 [20], whereas the “tardy” class is a novel category based on steadily increasing expression toward the end of the cycle. Levels of gene expression for “late” and “tardy” genes were verified using qRT-PCR, also stressing the difference between these two classes. Moreover, genes coding for EB mRNA were also expressed in cluster 2, which did not show increased expression towards the end of the cycle. This supported the view that the “tardy” expression profile is not simply based on accumulation of EB or reinfection.

The fact that all *Chlamydia* and *Chlamydophila* species encode two alternative sigma factors suggested a role for these in chlamydial gene regulation. Two reports have published promoters of the alternative sigma 28 factor for *Ctr* [79,80]. The *Cpn* orthologues h*ctB, bioB,* and *pgk* were present in the significant acute data of this study and were clustered as “tardy”, “mid”, and “early” genes, respectively. *Pgk* has been reported to be regulated by the σ^66^ and σ^ 28^ promoters [80]. This might explain the early gene expression for *pgk*. Expression profiles for *tsp* and *dnaK,* which were not calculated as significant in this study, showed an increased expression towards the end of the cycle (Figure 2, *Cpn0555, tsp,* category “Protein fate”, and *Cpn503, dnaK,* category “Chaperones”). Not all genes reported to be dependent on σ^ 28^ showed “tardy” expression profiles. We conclude that the different expression profiles of the “late” and “tardy” classes might be more complexly regulated than only by different sigma factors.

We also compared the EB transcriptome and published EB proteasome data with expression during acute infection. This comparison revealed that genes coding for EB mRNA were mainly associated with the “tardy” class, whereas genes coding for EB proteins were mainly associated with the “late” class. It is unlikely that this observation is based on a technical bias, as high numbers of genes were identified for the EB transcriptome and proteasome. Only a partial, but not significant, overlap was observed for EB mRNA in cluster 10 and EB proteins in cluster 11 (e.g., *omcB, pmp7*). The unexpected result that genes expressed toward the end of the cycle (“late” and “tardy”) did not contribute equally to EB mRNA and EB proteins can have many explanations. One possibility is that several genes might code for secreted factors, as we demonstrated that potential secreted effectors showed a “tardy” expression profile. Moreover, several genes of the “tardy” cluster 12 were also upregulated at initial stages of the cycle (6 hpi), either resulting from de novo synthesis or possibly reflecting carryover of mRNA. It has been recently shown that RNA carryover from the end into initial stages of the cycle is possible [78]. No evidence exists to date that carryover mRNA is functional at initial time points. Douglas and Hatch [52] have demonstrated that “carried-over” *omcB* mRNA does not result in protein synthesis early in infection. However, *omcB* was correlated with an EB-specific protein, whereas most genes located in the “tardy” class are not. Some genes even imply functions early in the cycle, including *guaB,* involved in de novo purine biosynthesis, *glgC,* catalyzing the conversion of glucose-1-phosphate to ADP-glucose, and *pgi,* which converts glucose-6-phosphate and fructose-6-phosphate during glycolysis. It has been shown in Escherichia coli that *glgC* transcription can be uncoupled from translation [94]; however, no similar mechanism has yet been proposed for Chlamydiaceae. Further analysis will be needed to clarify the discrepancy between “late” and “tardy” genes and their association with EB mRNA and proteins.

The categorization of 12 clusters and four cluster classes enabled the description of time-specific functions of genes belonging to the defined clusters. Differences in the cluster profiles of the “early” clusters 1 and 2 lead to the conclusion that genes of cluster 1 mediate processes at the beginning of the cycle, whereas genes of cluster 2 play a role later in the cycle. This is also consistent with the finding that the bulk of EB mRNA correlates with genes of cluster 2. The *incC* gene, coding for an inclusion membrane protein, has been defined as an “early” gene for *Ctr* [43], whereas it was grouped into the “mid” class for *Cpn,* because its expression peaked at 18 hpi. This classification corresponds well with the morphological changes, since the inclusion size increases greatly at mid-cycle [16]. Using expression profiles, we were able to more precisely group the expression of genes coding for membrane proteins, secretion processes, transcription, translation, and metabolic pathways. Proteins exposed on the surface are primary mediators in pathogen infection and transmission. It has been proposed that the family of Pmps have different roles in chlamydial biology [67,95,96]. Whereas a minority of pmp genes were expressed at initial stages of the cycle, most genes were linked to “late” and “tardy” clusters, suggesting a role in RB-to-EB redifferentiation. Classical markers for redifferentiation, such as *omcB, hctB,* and *lcrH1,* were expressed at the end of the cycle [20]. Chlamydiaceae possess only *ftsY* and *ftsW,* but not *ftsZ* present in E. coli [68,69]. Therefore, a role for peptidoglycans has been discussed not only in the transition from RB to EB prior to their release, but also in RB division [97]. Of the peptidoglycan genes, *murE* is grouped in the “early” cluster class, and *murA, murC, murD,* and *murF* are included in the “late” cluster class*.* Genes involved in cell division, such as *ftsY* and *ftsW,* were most strongly expressed mid-cycle. This indicates that *murE* may be involved in binary fission, whereas it is most likely that the remainder of the peptidoglycan genes play a role in RB-to-EB redifferentiation. *Cpn* uses different energy and metabolic pathways throughout the cycle. While glycolysis and ATP production are the focal elements at early time points, acquisition of host intermediates gains increasing importance to maintain the developmental cycle at later time points. It is likely that toward the end of the cycle, reverse reactions support glycogenesis and the pentose phosphate pathway. Protein secretion is crucial for *Cpn* pathogenicity. The putative chlamydial TTSS is proposed to play a key role in the host–pathogen interaction by channeling different effectors into the host cell [74]. We were able to show that the TTSS is expressed late in the cycle, whereas the TIISS expression peaked early to mid-cycle. Genes coding for putative TTSS proteins were shown to belong either to the “early”, “mid”, or “tardy” classes of the acute infection, with only those of the “tardy” class being downregulated during persistence. Moreover, only the TIISS translocase *secA1* was upregulated during persistence. The presence of multiple SecA proteins in a single bacterium is unusual and only shared with a few other pathogenic bacteria [98]. It has been shown that SecA homologs in Mycobacterium tuberculosis can have different functions [99]. In summary, it can be assumed that *Cpn* gene expression paves the way for the different requirements of the pathogen during the developmental cycle, and identification of these patterns leads to a deeper insight into processes during the acute infection.

In addition, the altered expression profile of the *Cpn* transcriptome during persistence was analyzed and compared to that of the acute infection. Upregulated genes during iron depletion were significantly linked to clusters 2 and 3 at the beginning of the cycle, whereas downregulated genes during iron depletion were significantly linked to clusters 10, 11, and 12 at the end of the cycle. Therefore, persistence mediated by iron depletion can be characterized as the result of an arrest in transcriptional regulation rather than an adapted change to environmental signals, and it can be concluded that blockage of the “transcriptome clockwork” leads to persistence. Genes responsible for transcription and translation were largely downregulated during persistence. Additionally, a multiplicity of Pmps located in the acute clusters 10 and 11 showed downregulation during persistence indicating a central role of these genes in RB-to-EB redifferentiation. Moreover, the acute clusters 8 and 9 were significantly linked with the EB protein, and were not significantly affected during *Cpn* persistence. We draw the conclusion that the majority of late genes contributing to the EB protein content are not the driving force for RB-to-EB redifferentiation as they are largely unaltered during persistence. This suggests that EB proteins are made before initiation of the redifferentiation and that the redifferentiation process itself is initiated only by a limited number of genes. A recent report for *Ctr* treated with IFN-γ showed that genes required for DNA repair, phospholipid biosynthesis, and translation, including many early cycle genes such as *euo,* were strongly upregulated [44]. Genes that were downregulated in *Ctr* treated with IFN-γ were genes having functions in RB-to-EB differentiation, such as *hctA, hctB, ompB,* and *ompC*. These data are highly comparable with the data presented here for *Cpn* treated with DAM. However, in contrast to other published reports [44,47,48,100], a recent report showed that transcription of *euo, omcB, hctA, hctB,* and *lcrH1* are upregulated during IFN-γ persistence, whereas treatment with penicillin leads to transcriptional downregulation [53]. We were able to show that *omcB, hctB,* and *lcrH1* transcripts were reduced during iron depletion–mediated persistence, whereas *euo* and *hctA* expression was unaltered. It has been suggested that major functions are downregulated in *Cps,* whereas other genes display considerable variation between the response patterns due to different persistence models [45]. This might also be the case for *Cpn.* It would be interesting to investigate whether key transcriptional regulators, such as Fur, a strictly iron-regulated DNA-binding protein, play a major role in the establishment of persistence in the DAM model. Furthermore, global comparison of transcriptional changes in different persistence models, including persistence induced by treatment with IFN-γ or antibiotics, will yield a broader insight into the phenomenon of persistence in future. Experiments to answer these questions are in progress.

### Concluding Remarks

In summary, we identified different transcriptional stages throughout the developmental cycle of the obligate intracellular pathogen *Cpn* and during the iron depletion–mediated persistent infection. We also discovered remarkable connections relevant to the intracellular life style and developmental cycle of this bacterium. The analysis revealed a strong similarity of iron-depleted persistence to stages of the acute developmental cycle. As iron also has an important influence on infection, and host and pathogen compete for this precious element, the strategies used by pathogens to overrule iron-limiting host defense mechanisms are increasingly important. Investigating persistency models is the key for understanding chronic infections in vivo, and will also support the search for chlamydial effectors and mechanisms of pathogenesis.

## Materials and Methods

### Eukaryotic host cells.

The HEp-2 human epithelial cell line (ATCC-CCL23) derived from a laryngeal carcinoma was grown in RPMI medium (Gibco, http://www.invitrogen.com) with 10% fbs (Biochrom, http://www.biochrom.de) at 37 °C and 5% CO_2_. Cells were regularly checked for mycoplasma contamination.

### Propagation and infection.

The CWL029 *Cpn* strain (ATCC VR1310) was propagated in HEp-2 cells. Cells were infected with a multiplicity of infection (MOI) of 2 in RPMI medium without supplements, centrifuged at 37 °C for 1 h at 2,100 revolutions per min (rpm), and incubated at 35 °C and 5% CO_2_ for 1 h. Afterwards, medium was replaced with RPMI medium supplemented with 5% fbs, containing cycloheximide (chex) (1 μg/ml). Cultures were incubated for 72 h at 35 °C and harvested using glass beads (Sigma, http://www.sigmaaldrich.com), and the supernatant was transferred to a 50-ml tube (Greiner, http://www.greinerbioone.com). Host cells were lysed using glass beads (Sigma) and centrifuged at 800 rpm for 10 min to pellet cell debris. The supernatant was centrifuged at 10,000 rpm for 1 h to pellet EB. The pellet was washed with sucrose phosphate glutamate (SPG) buffer (pH 7.4), recentrifuged and resolved in SPG buffer, aliquoted, and stored at −80° C. Stocks were determined to be free of mycoplasma contamination. For acute and persistent infections, HEp-2 cells were infected with *Cpn* at a MOI of 40 for the 6- and 12-hpi time points, and an MOI of 15 for all other time points. The time course started upon infection (Figure 1). To generate persistent infections, 30 μM of DAM was added to the medium throughout the infection process starting at the time of infection. Cells were incubated at 35 °C and 5% CO_2_ until the respective time points, washed with prewarmed PBS, and harvested with 1 ml TRIzol (Invitrogen, http://www.invitrogen.com) per well. The bacteria were examined by staining cell cultures grown on coverslips with an anti-LPS antibody (Progen, http://www.progen.de) to confirm proper growth and establishment of acute and persistent infections.

### RNA Isolation, labeling, and quality control.

*Cpn*-infected HEp-2 cells were lysed with TRIzol. Total RNA was isolated using a modified protocol. For a detailed description of the modified protocol, see Table S1. Integrity of the total RNA was verified using the Bioanalyzer 2100 (Agilent, http://www.chem.agilent.com), and RNA was quantified by photometer and Nanodrop (Kisker, http://www.kisker-biotech.com) measurements prior to labeling. In addition, 50 μg of total RNA for the 6- and 12-hpi time points was hybridized with 20 μg of total RNA from the 48-hpi time point, and was purified using the MICROBEnrich (Ambion, http://www.ambion.com) procedure to increase signal intensities.

A modified protocol for indirect incorporation of aminoallyl nucleotides during a first-strand reverse transcription reaction was established to yield a maximum of labeled cDNA copies of the total RNA pool for *Cpn.* Each labeling reaction consisted of 20 μg of total RNA (1 μg/μl) (or the yield of 50 μg of total RNA processed with the MICROBEnrich procedure), 2 μg random nonamer primers (Amersham, http://www.amersham.com), 4.0 ml of dNTP master mix, and 2 μl (4 units/μl) of Superscript II reverse transcriptase (Stratagene, http://www.stratagene.com). RNA and primers were heated to 65 °C for 10 min, slowly cooled to room temperature, snap cooled on ice before the remaining components were added, and incubated at 42 °C for 2 h. RNA was degraded by adding 10 μl (2.5 M) NaOH, incubating at 37 °C for 15 min, and neutralizing with 20 μl (2 M) HEPES. cDNA was precipitated by adding 6 μl (3 M) sodiumacetate and 150 μl EtOH, then incubated at −20 °C overnight and pelleted with 13,000 rpm at 4 °C. The pellet was resuspended in 0.1 M sodiumbicarbonate buffer (pH 9.0), added to either Cy 3 or Cy 5 mono NHS esters (Amersham), incubated at room temperature for 90 min, and purified using 30K Microcon spin columns (Millipore, http://www.millipore.com). The elution volume was diluted using hybridization buffer (Ambion) and quantified at A_552nm_ for Cy 3 and A_650nm_ for Cy 5.

### EB purification.

Purification was done as described by Mukhopadhyay [101], with slight modifications. Briefly, 10^8^ cells were lysed in 5 ml of NP-40 (Fluka, http://www.sigmaaldrich.com) complemented SPG buffer (1% NP-40 per 1 × 10^7^ cells) and were subjected to sonication for 5 min, followed by a 20-min incubation step with DNAse I (1.6 μg/ml) (Grade II; Roche, http://www.roche.com) on ice. Nuclei and bigger fragments were removed by a 5-min centrifugation step at 4,000 rpm. Post nuclear supernatant was layered on top of a 7-ml 50% and 25-ml 20% Ficoll-400 (Pharmacia, http://www.pifzer.com) step gradient and was centrifuged at 18,500*g* for 1 h at 4 °C in a swinging bucket rotor. The interphase was collected and washed by addition of 10 ml of SPG buffer and centrifuged at 18,500*g* for 30 min. The pellet was resuspended by 5 min sonication to dissolve EB. Purity was assessed by electron microscopy on a Leo 906E Transmission Electron Microscope.

### Array design, hybridization, and scanning.

A 50-mer oligo array containing all open reading frames of the genome projects of CWL029, AR39 (http://www.ncbi.nlm.nih.gov/entrez/query.fcgi?db=genomeprj), and J138 (http://kantaro.grt.kyushu-u.ac.jp/J138/ident/index.html and http://www.ncbi.nlm.nih.gov/entrez/query.fcgi?db=genomeprj) was designed. To ensure maximum specificity, oligo sequences were compared for homology against the human genome using the BLAST algorithm. Design and spotting was done by MWG-Biotech AG (http://www.mwg-biotech.com). Cross-hybridization of eukaryotic RNA was confirmed to be marginal by labeling and hybridizing eukaryotic total RNA with the described procedure. Arrays were blocked prior to hybridization by incubation for 45 min at 42 °C in blocking solution (4x SSC, 0.5% SDS, 1% BSA), washed five times with H_2_0, and dried by centrifugation (1,000 rpm, RT, 2 min). Microarray experiments were done as two-color hybridizations. In order to compensate for dye-specific effects and to ensure statistically relevant data, a color swap was performed. Reverse transcribed cDNA pools were mixed with hybridization buffer (Ambion), denatured, snap-cooled, pipetted onto the array using LifterSlips (Erie, http://www.eriemicroarray.com), and placed in a sealed humidified hybridization chamber (Scienion, http://www.scienion.de) at 42 °C for 48 h without shaking. After hybridization, the array was washed and dried according to the Ambion protocol. The microarray data discussed in this publication have been deposited in the National Center for Biotechnology Information Gene Expression Omnibus (http://www.ncbi.nlm.nih.gov/projects/geo) under GEO Series number GSE7070.

### Data analysis and statistics.

Scanning of microarrays was performed with 5 μm resolution using a microarray laser scanner (Agilent). Features were extracted using the Agilent Technologies image analysis software (version A7.5) using default settings for non-Agilent microarrays. A local background subtraction method was used and the background was adjusted globally to zero. Dye normalization was done using the rank consistency method and applying a local weighted regression normalization (LOWESS). The ratio between both channels, the log ratio error, and the *p*-values were calculated using default settings. Data analysis was carried out on the Rosetta Inpharmatics platform Resolver Build 4.2 (http://www.rosettabiosoftware.com). Transcriptome analysis was carried out with two biological replicates for 36 hpi and at least three biological replicates for all other time points. Samples were derived from independent infections, RNA preparations, labeling reactions, and hybridizations. We selected genes using a 1.8-fold expression cutoff. All data of the individual sets comprising different time points were combined as ratio experiments using negative polarity for the color swap dye-reversal hybridizations. This was achieved by combining the ratio profiles, representing single hybridizations, and using an error-weighted average based on *p*-values.

In addition, the microarray data were statistically analyzed using the SAM algorithm version 2.0 (http://www-stat.stanford.edu/~tibs/SAM). SAM uses the standard deviation of repeated gene expression measurements to assign a score to each gene and estimates a false discovery rate by permutations of the data. This analysis ascertains whether genes identified as differentially expressed could arise from a random fluctuation of the large quantity of data generated. To identify genes whose expression differed significantly during the acute time course, we performed a one-class time course analysis with log2 data taken from ratio profiles. For dye swap experiments, fold-change data were reversed, converted into ratio values, and log2 transformed. We applied an FDR of 0.46% and a delta value of 0.49 for the cycle of development. In addition, the FDR was calculated separately for each time point of the acute and persistent infection using a one-class analysis. Calculations for the acute infection and the persistent infection are depicted in Tables S2 and S7A–C. For the persistent infection, we applied FDRs of 1.564%, 18.6%, and 1.33%, and a delta of 0.73, 0.17, and 0.327 for 24, 48, and 72 hpi, respectively. Significant genes were combined for further analysis. The microarray data of SAM-positive genes were extracted using the Samster software [102], and ratio profiles or ratio experiments, representing all hybridizations derived from one time point, were clustered with Gene Cluster [103] using a SOM. Cluster calculations were displayed using TreeView (http://rana.lbl.gov/EisenSoftware.htm).

### qRT-PCR and normalization.

Primers for qRT-PCR (Table S10) were designed for selected genes using Primer3 (http://frodo.wi.mit.edu/cgi-bin/primer3/primer3_www.cgi) and checked for homology to the human genome using BLAST algorithm to achieve a high probability for *Cpn*-specific products. Product specificity was controlled using the denaturing protocol of the ABI Prism 7000 program (Applied Biosystems, http://www.appliedbiosystems.com). Standard curves were performed using total DNA from infected HEp-2 cells to measure the primer pair efficiency. qRT-PCR was performed in duplicate for at least three biological replicates. RNA was transcribed into cDNA using 2 μg of total RNA, random nonamer primers (Amersham), and the Omniscript RT Kit (Qiagen, http://www.qiagen.com) according to the manufacturers' instructions. The RT product (20 μl) was diluted with 500 μl H_2_O, and qRT-PCR was performed using 5 μl of the RT product per well. Primer pairs, c-DNA, and SYBER-green were combined according to the manufacturer's instructions. SYBR-green uptake in double-stranded DNA was measured on an ABI Prism 7000 thermocyler (Applied Biosystems). Ten putative housekeeping genes, including 16S rRNA, were chosen based on indications in the literature and our microarray data to calculate an HKI for normalization purposes. The qRT-PCR experiments were performed for all time points, and the accuracy of the chosen housekeeping candidates was calculated using the geNorm program (http://medgen.ugent.be/~jvdesomp/genorm). A set of the three most accurate housekeeping genes was used to define a HKI and to normalize gene expression. Relative quantification was performed using the 2^(−ΔΔ CP)^ method, including an efficiency correction [93] for the primers.

### Microscopy.

To analyze the growth of C. pneumoniae grown under iron-deficient conditions, infected host cells were fixed with paraformaldehyde and labeled with an anti-LPS antibody (Progen).

## Supporting Information

Figure S1 Comparison of RNA Isolation Methods for *Cpn* and RNA Isolation with and without the Use of Sonication and Glycogen (A) C. pneumoniae–infected HEp-2 cells (MOI = 15) were lysed at 48 hpi using the TRIzol protocol without the use of glycogen RNA and without a sonication step (a), with 5 min sonication, (b) and with 5 min sonication and the use of glycogen (c). The electropherograms obtained with the Agilent Bioanalyzer 2100 software show 16S (grey arrowhead), 18S (grey arrow), 23S (black arrowhead), and 28S (black arrow) rRNA. The measured fluorescence for the 28S rRNA was set to 100%.
(B) Ratio of prokaryotic 23S rRNA versus eukaryotic 28S rRNA for the electropherograms (a–c). The area for each peak was measured with the Bioanalyzer 2100 software. Sonication and sonication plus the use of glycogen lead to increased recovery of chlamydial rRNA in comparison to eukaryotic rRNA.
(67 KB PDF)

Figure S2 Compare Blot for Hybridizations with Enriched and Not Enriched RNA RNA was extracted at 48 hpi for the acute infection and the iron-mediated persistent infection. Samples were split and half was labeled as described. The second half was enriched for *Cpn* RNA using MICROBEnrich prior to labeling. Samples for the enriched and non-enriched samples were hybridized (+Cpn −DAM versus +Cpn +DAM) and compared using a compare blot. Most of the genes showed unchanged or correlated expression. Only six genes were calculated as anticorrelated expressed using Rosetta Resolver software.
(114 KB PDF)

Figure S3 Bioanalyzer 2100 Profiles of the Acute and the Iron Depletion Infections The electropherogram was obtained using Agilent Bioanalyzer 2100 software. C. pneumoniae–infected HEp-2 cells were lysed at the indicated time points with the described TRIzol protocol. The sample for 120 hpi was repleted (DAM-containing medium replaced with normal medium) at 72 hpi. Peaks indicate the amount of bacterial (arrowheads: 16S rRNA and 23S rRNA) and host rRNA (arrows: 18S rRNA and 28S rRNA) in the RNA pool. Fluorescence measure for the 28S rRNA was set to 100%. Compared to the host cell rRNA, the amount of bacterial rRNA increased throughout the developmental cycle. During DAM-mediated iron-depletion persistence, only small peaks were observed. After repletion of the persistent infection, 16S rRNA and 23S rRNA amounts indicated the resumption of the developmental cycle.
(17 KB PDF)

Figure S4 Fluorescence Pictures during Acute and DAM-Mediated Iron Depletion Infections HEp-2 cells were infected with C. pneumoniae (MOI = 5) for the acute infection, and additionally incubated with DAM to obtain a persistent infection. To determine the progeny of the acute and persistent infection, the infected cells were lysed after 72 hpi and HEp-2 cells infected with a 1:250 dilution. The bar indicates 10 μm.
(757 KB PDF)

Figure S5 Quantitative Determination of the Progeny of the Acute and Persistent Infection Cells were infected with C. pneumoniae (MOI = 5) for the acute infection and additionally incubated with DAM to obtain a persistent infection. To determine the progeny of the acute and persistent infection, the infected cells were lysed after 72 hpi and HEp-2 cells infected with a 1:250 dilution. The measured EB amount for the acute infection was set to 100%. Persistent infection led to drastically reduced progeny (3.3%). Error bars display standard deviation from five independent counts.
(5 KB PDF)

Figure S6 One Step Growth Curve of the *Cpn* EB Progeny throughout the Acute Infection HEp-2 cells were infected with C. pneumoniae (MOI = 1) for the acute infection, and the EB progeny in the supernatant and in attached cells was measured after 24, 48, 72, 96, and 120 hpi. Supernatant included detached cells. Cells were washed, and the values measured for the attached cells counted in these samples. Following lysis using glass beads, HEp-2 cells were infected with either a 1:10, 1:100, or 1:1000 dilution of the lysate of the supernatant and attached cells. Inclusions were counted using labeled LPS antibody. The EB progeny was calculated, and represented the amount if progeny for one infectious particle used in the first infection. Each experiment was performed with five technical and at least two biological replicates. No EBs were detectable at 24 and 48 hpi in the supernatant and attached cells. In the attached cells, the highest amount of EBs was present at 72 hpi, decreasing at later time points. The amount of EBs in the supernatant increased from 72 hpi until 120 hpi. This is most likely because of the release of EBs and the detachment of infected cells at later time points.
(7 KB PDF)

Figure S7 Electron Microscopy of the EB Purification EBs were stained by negative contrasting. (A) Gradient-purified *Cpn,* (B) post nuclear supernatant after two washes with sucrose phosphate buffer before gradient purification. White arrows indicate EBs, black arrows show cellular components before gradient purification. Bar = 2 μm.
(91 KB PDF)

Figure S8 Gaussian Kernel Density Distribution and LACK Significance Calculation with Genes Coding for EB mRNA A Gaussian Kernel Density Distribution (http://www.wessa.net) was calculated for genes coding for EB mRNA using the SOM clustering of the 754 genes of the acute infection (Figure 1). It can be seen that genes coding for EB mRNA showed a peak density for “early” and “tardy” clusters. This correlates with the calculation done with the LACK software showing that genes coding for EB mRNA were only significantly linked to clusters 2, 11, and 12 (Table S5).
(23 KB PDF)

Figure S9 Gaussian Kernel Density Distribution and LACK Significance Calculation with Genes Coding for Proteins Present in EB A Gaussian Kernel Density Distribution (http://www.wessa.net) was calculated for genes coding for proteins present in EB using the SOM clustering of the 754 genes of the acute infection (Figure 1). It can be seen that genes coding proteins present in EB showed a peak density for “mid” clusters. This correlates with the calculation done with the LACK software showing that genes coding for proteins present in EBs are only significantly linked to clusters 8 and 9 (Table S6).
(12 KB PDF)

Figure S10 Gaussian Kernel Density Distribution and LACK Significance Calculation with Genes Upregulated in Iron Depletion–Mediated Persistence A Gaussian Kernel Density Distribution (http://www.wessa.net) was calculated for genes coding for genes upregulated in iron depletion–mediated persistence using the SOM clustering of the 754 genes of the acute infection (Figure 1). Genes upregulated during iron depletion–mediated persistence showed peak density clusters at the beginning of the cycle. This correlates with the calculation done with the LACK software showing that genes upregulated in iron depletion–mediated persistence were only significantly linked to clusters 2 and 3 (Table S8).
(17 KB PDF)

Figure S11 Gaussian Kernel Density Distribution and LACK Significance Calculation with Genes Downregulated in Iron Depletion–Mediated Persistence A Gaussian Kernel Density Distribution (http://www.wessa.net) was calculated for genes downregulated during iron depletion–mediated persistence using SOM clustering of the 754 genes of the acute infection (Figure 1). Genes downregulated during iron depletion–mediated persistence showed peak density clusters at the beginning of the cycle. This correlates with the calculation done using the LACK software, showing that genes upregulated during iron depletion–mediated persistence were only significantly linked to clusters 10, 11, and 12 (Table S9).
(16 KB PDF)

Table S1 Modified TRIzol Protocol This RNA extraction protocol based on the original TRIzol protocol led to an increased recovery of *Cpn* RNA compared to the unmodified protocol (see also Figure S1).
(14 KB PDF)

Table S2 One-Class SAM Calculations for the Acute Infection Significant expression profiles during the acute infection were calculated using SAM. Data are displayed in log2 (ratio) values. For further explanation, see Materials and Methods or http://www-tat.stanford.edu/~tibs/SAM.
(136 KB PDF)

Table S3 Genes Clustered in Figure 1B and Figure 4B Using SAM, 754 genes for the acute and 461 genes for the persistent infection were calculated as being significantly regulated. These genes are listed. Data are displayed in log2 values. (A) Genes clustered in Figure 1B and (B) Figure 4B.
(112 KB PDF)

Table S4 EB mRNA Content A list of the 91 mRNA transcripts identified in the *Cpn* EB
(17 KB PDF)

Table S5 LACK Calculations for EB mRNA Content The correlation between genes coding for EB mRNA transcripts and gene classes of the acute infection was calculated using LACK software. Also see Figure S8.
(13 KB PDF)

Table S6 LACK Calculations for EB Proteins The correlation between genes coding for EB proteins and gene classes of the acute infection was calculated using LACK software. Also see Figure S9.
(6 KB PDF)

Table S7 One-Class SAM Calculations for Persistent Infection at 24 hpi (A) Significant expression profiles at 24 hpi during persistent infection were calculated using SAM. Data are displayed in log2 (ratio) values. For further explanation, see Materials and Methods or http://www-tat.stanford.edu/~tibs/SAM.
(B) Significant expression profiles at 48 hpi during persistent infection were calculated using SAM. Data are displayed in log2 (ratio) values. For further explanation, see Materials and Methods or http://www-tat.stanford.edu/~tibs/SAM.
(C) Significant expression profiles at 72 hpi during persistent infection were calculated using SAM. Data are displayed in log2 (ratio) values. For further explanation, see Materials and Methods or http://www-tat.stanford.edu/~tibs/SAM.
(271 KB PDF)

Table S8 LACK Calculations for Upregulated Genes The correlation between genes upregulated during persistent infection and gene classes of the acute infection was calculated using LACK software. Also see Figure S10.
(13 KB PDF)

Table S9 LACK Calculations Downregulated Genes The correlation between genes downregulated during persistent infection and gene classes of the acute infection was calculated using LACK software. Also see Figure S11.
(13 KB PDF)

Table S10 Real-Time PCR Primers Used in This Study (21 KB PDF)

Table S11 Determination and Stability of Housekeeping Gene Candidates together with Randomly Picked Control Genes (25 KB PDF)

Table S12 qRT-PCR Data for Different Time Points of the Acute and Iron Depletion–Mediated Persistence (40 KB PDF)

### Accession Number

The microarray data discussed in this publication have been deposited in the National Center for Biotechnology Information Gene Expression Omnibus (http://www.ncbi.nlm.nih.gov/projects/geo) and are accessible through GEO Series accession number GSE7070.

## Abbreviations

*Cpn*: *Chlamydophila pneumonia*
*Ctr*: *Chlamydia trachomatis*
DAM: deferoxamine-mesylate
EB: elementary body
FDR: false discovery rate
HKI: housekeeping index
hpi: hours post infection
IFN-γ: interferon γ
MOI: multiplicity of infection
Pmp: polymorphic membrane protein
qRT-PCR: quantitative real-time PCR
RB: reticulate body
SAM: Significance Analysis of Microarrays
SOM: self-organizing map
TIISS: type two secretion system
TTSS: type three secretion system
