# Supplementary material for: Gene Expression Profiles of Chlamydophila pneumoniae during the Developmental Cycle and Iron Depletion–Mediated Persistence
Source: PLoS Pathog. 2007 Jun 22;3(6):e83. doi: 10.1371/journal.ppat.0030083 (PMC1894823; doi:10.1371/journal.ppat.0030083)
Supplement: Supplementary file 1 [file ppat.0030083.sg001.pdf]

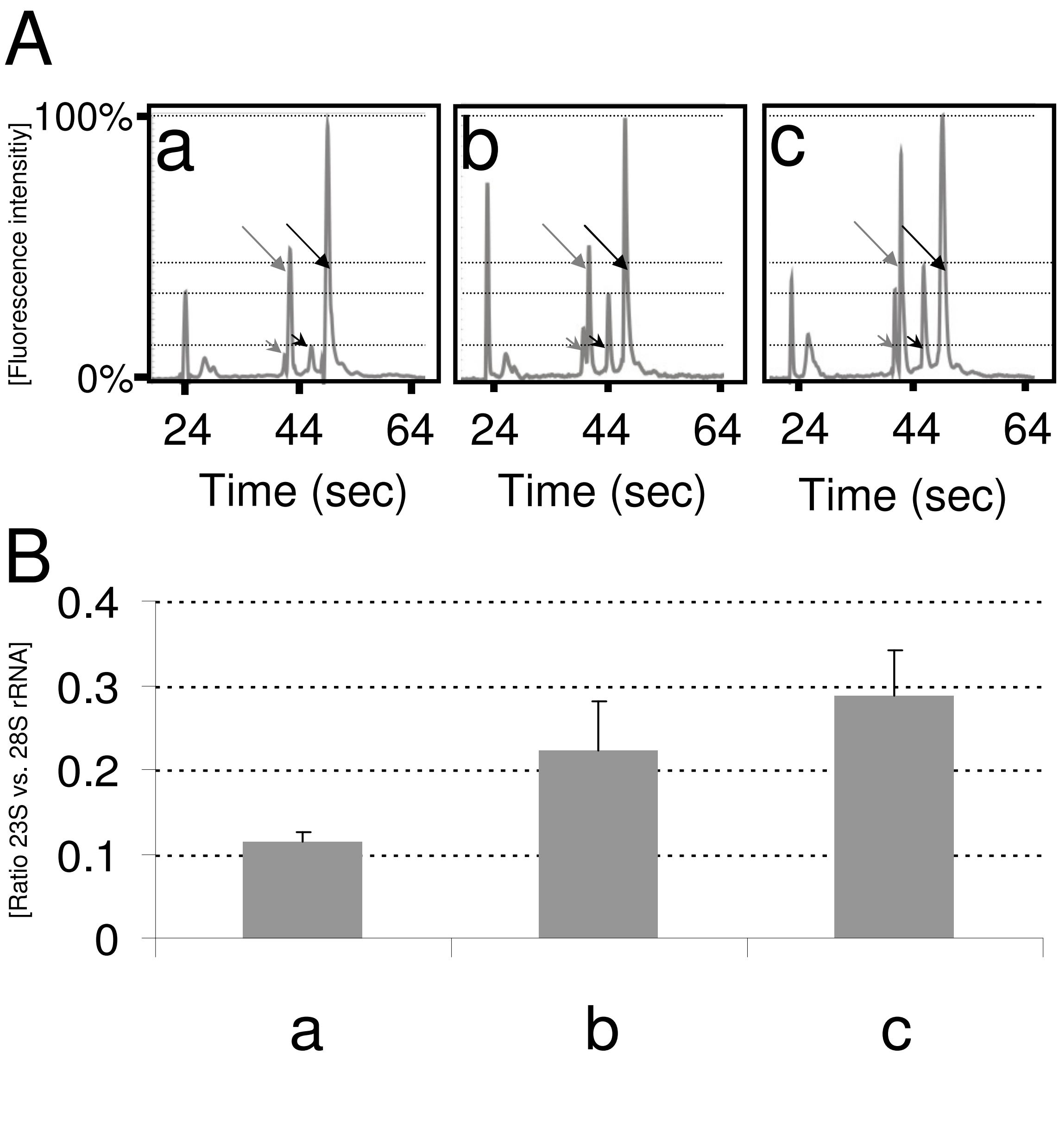

A
a
b
c
100%
0%
[Fluorescence intensitiy]
24
44
64
Time (sec)
B
0.4
0.3
0.2
0.1
0
[Ratio 23S vs. 28S rRNA]
a
b
c

FIGURE S1
