# Supplementary material for: Gene Expression Profiles of Chlamydophila pneumoniae during the Developmental Cycle and Iron Depletion–Mediated Persistence
Source: PLoS Pathog. 2007 Jun 22;3(6):e83. doi: 10.1371/journal.ppat.0030083 (PMC1894823; doi:10.1371/journal.ppat.0030083)
Supplement: Supplementary file 2 [file ppat.0030083.sg002.pdf]

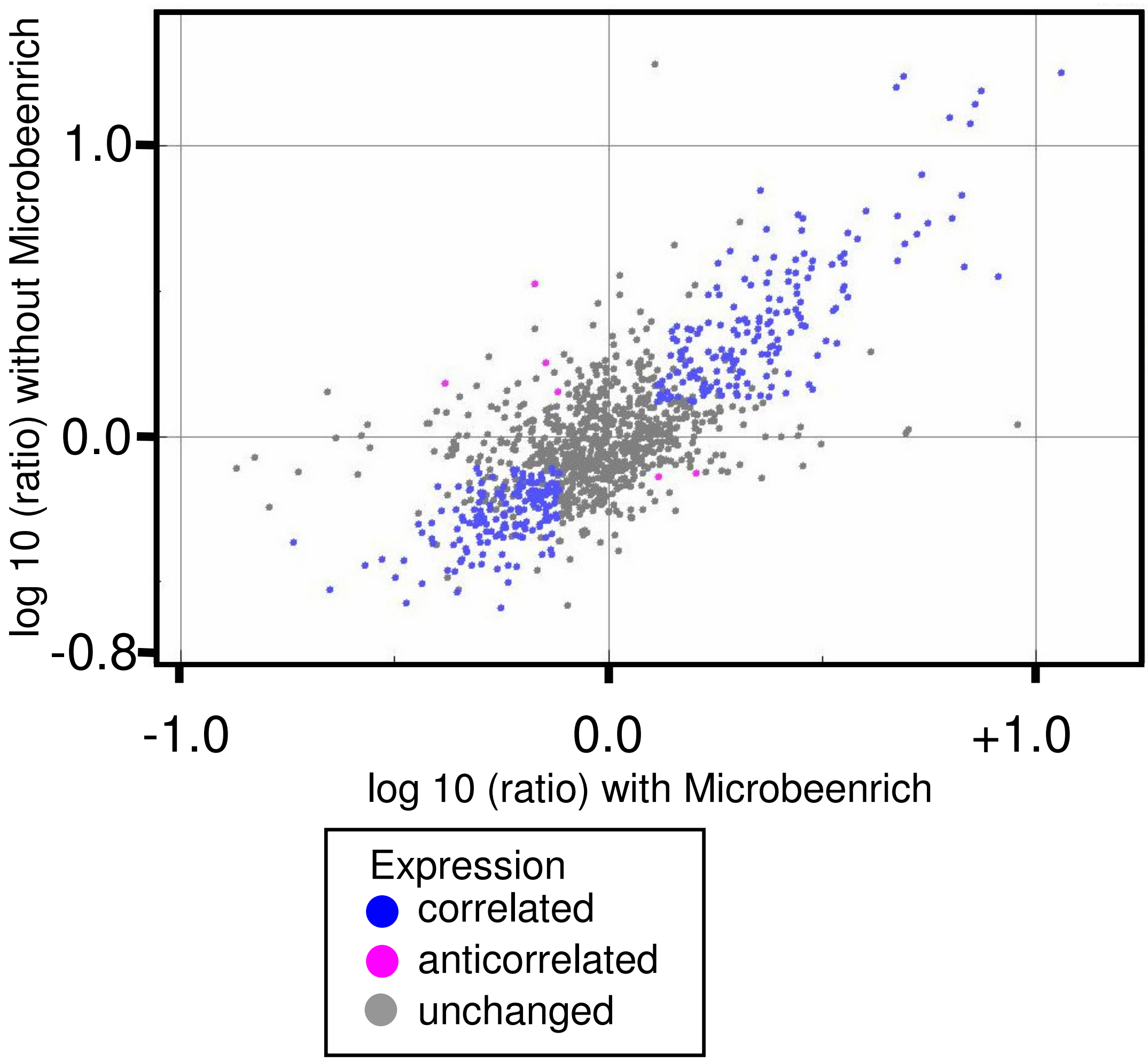
log 10 (ratio) without Microbeenrich
1.0
0.0
-0.8
-1.0
0.0
+1.0
log 10 (ratio) with Microbeenrich
Expression
correlated
anticorrelated
unchanged

FIGURE S2
