# Supplementary material for: Gene Expression Profiles of Chlamydophila pneumoniae during the Developmental Cycle and Iron Depletion–Mediated Persistence
Source: PLoS Pathog. 2007 Jun 22;3(6):e83. doi: 10.1371/journal.ppat.0030083 (PMC1894823; doi:10.1371/journal.ppat.0030083)
Supplement: Supplementary file 3 [file ppat.0030083.sg003.pdf]

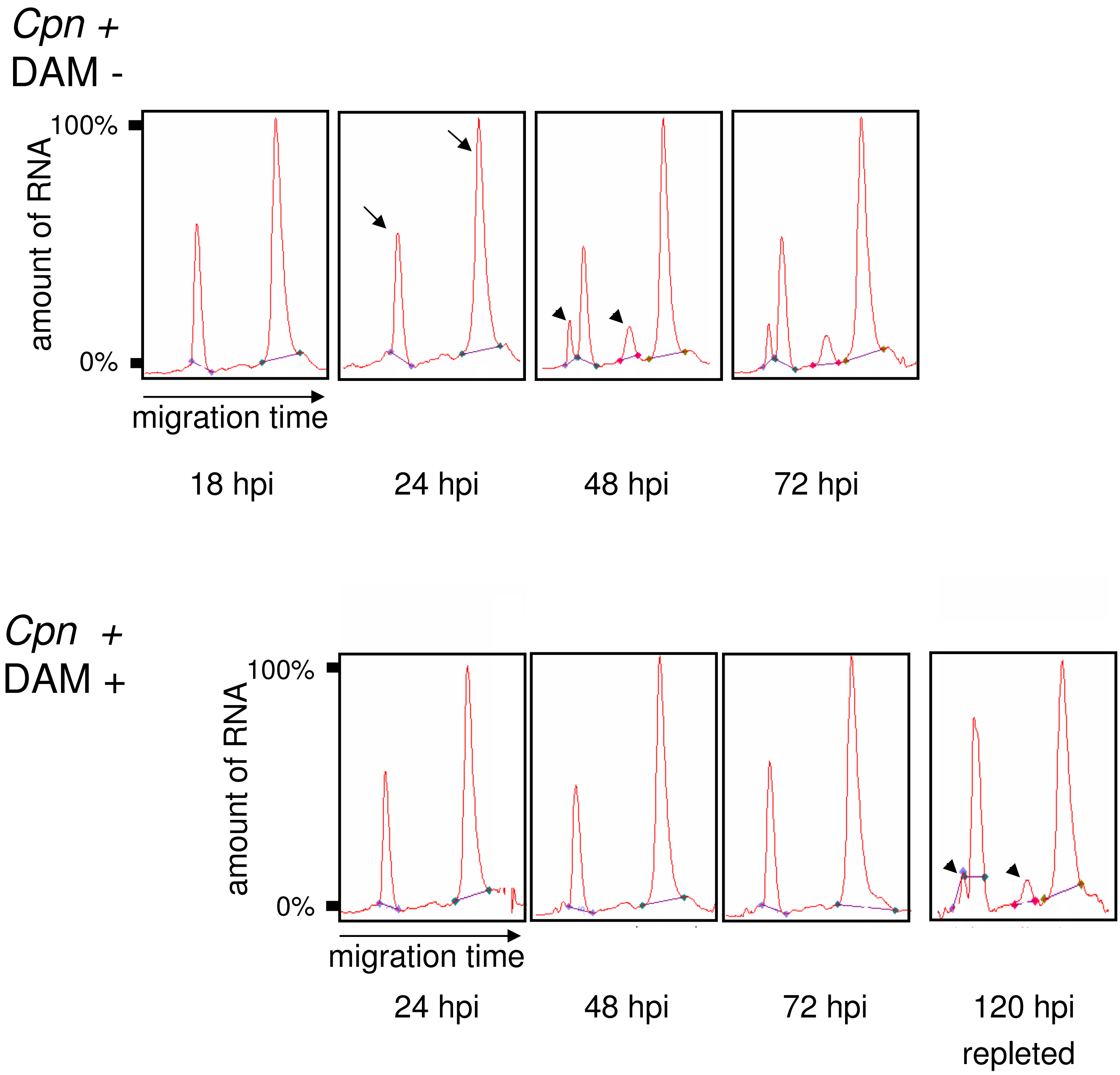
Cpn +
DAM -
100%
0%
amount of RNA
migration time
18 hpi
24 hpi
48 hpi
72 hpi
Cpn +
DAM +
100%
0%
amount of RNA
migration time
24 hpi
48 hpi
72 hpi
120 hpi
repleted

FIGURE S3
