# Supplementary material for: Gene Expression Profiles of Chlamydophila pneumoniae during the Developmental Cycle and Iron Depletion–Mediated Persistence
Source: PLoS Pathog. 2007 Jun 22;3(6):e83. doi: 10.1371/journal.ppat.0030083 (PMC1894823; doi:10.1371/journal.ppat.0030083)
Supplement: Supplementary file 4 [file ppat.0030083.sg004.pdf]

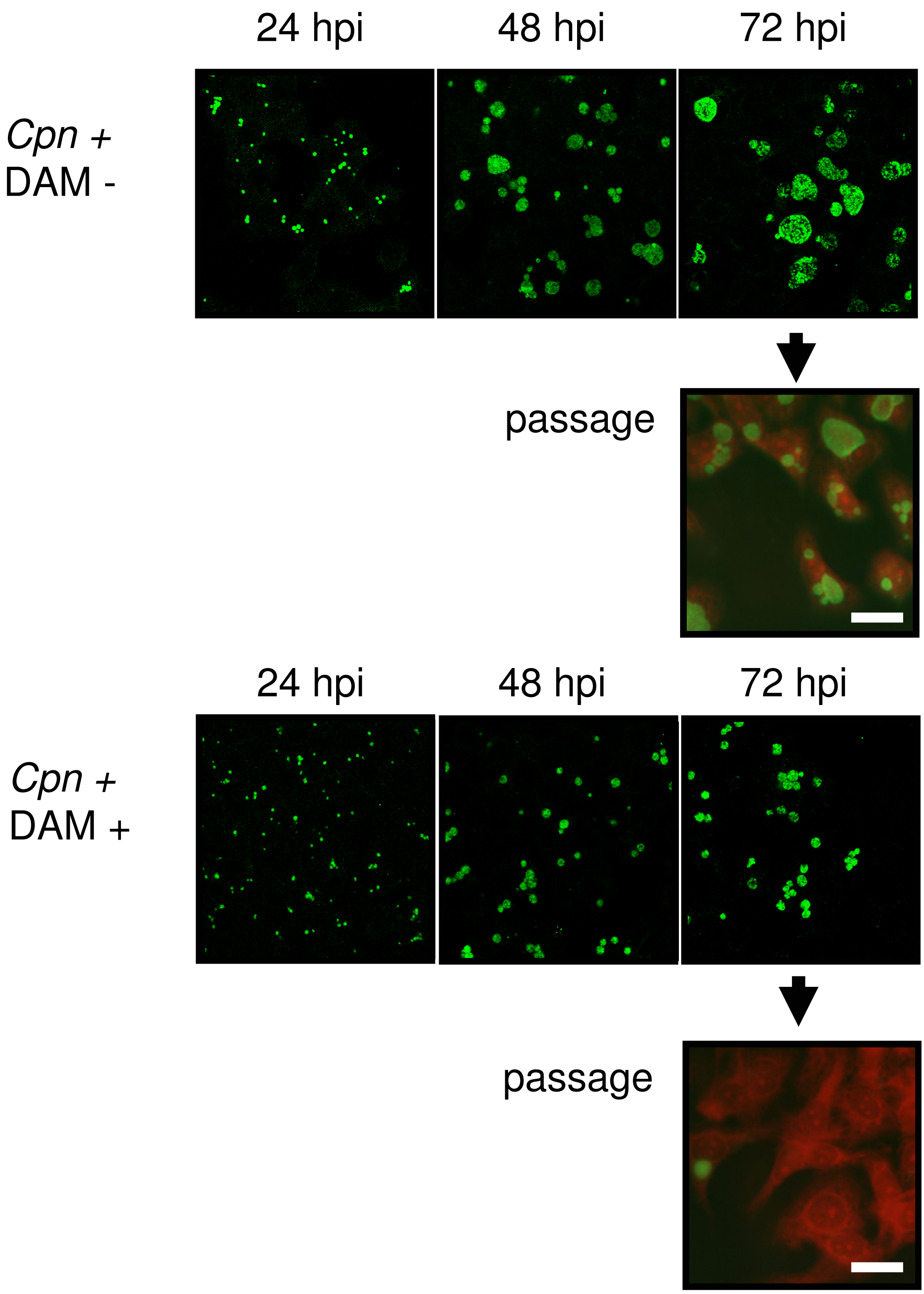
24 hpi
48 hpi
72 hpi
Cpn +
DAM -
passage
24 hpi
48 hpi
72 hpi
Cpn +
DAM +
passage

FIGURE S4
