# Supplementary material for: Gene Expression Profiles of Chlamydophila pneumoniae during the Developmental Cycle and Iron Depletion–Mediated Persistence
Source: PLoS Pathog. 2007 Jun 22;3(6):e83. doi: 10.1371/journal.ppat.0030083 (PMC1894823; doi:10.1371/journal.ppat.0030083)
Supplement: Supplementary file 6 [file ppat.0030083.sg006.pdf]

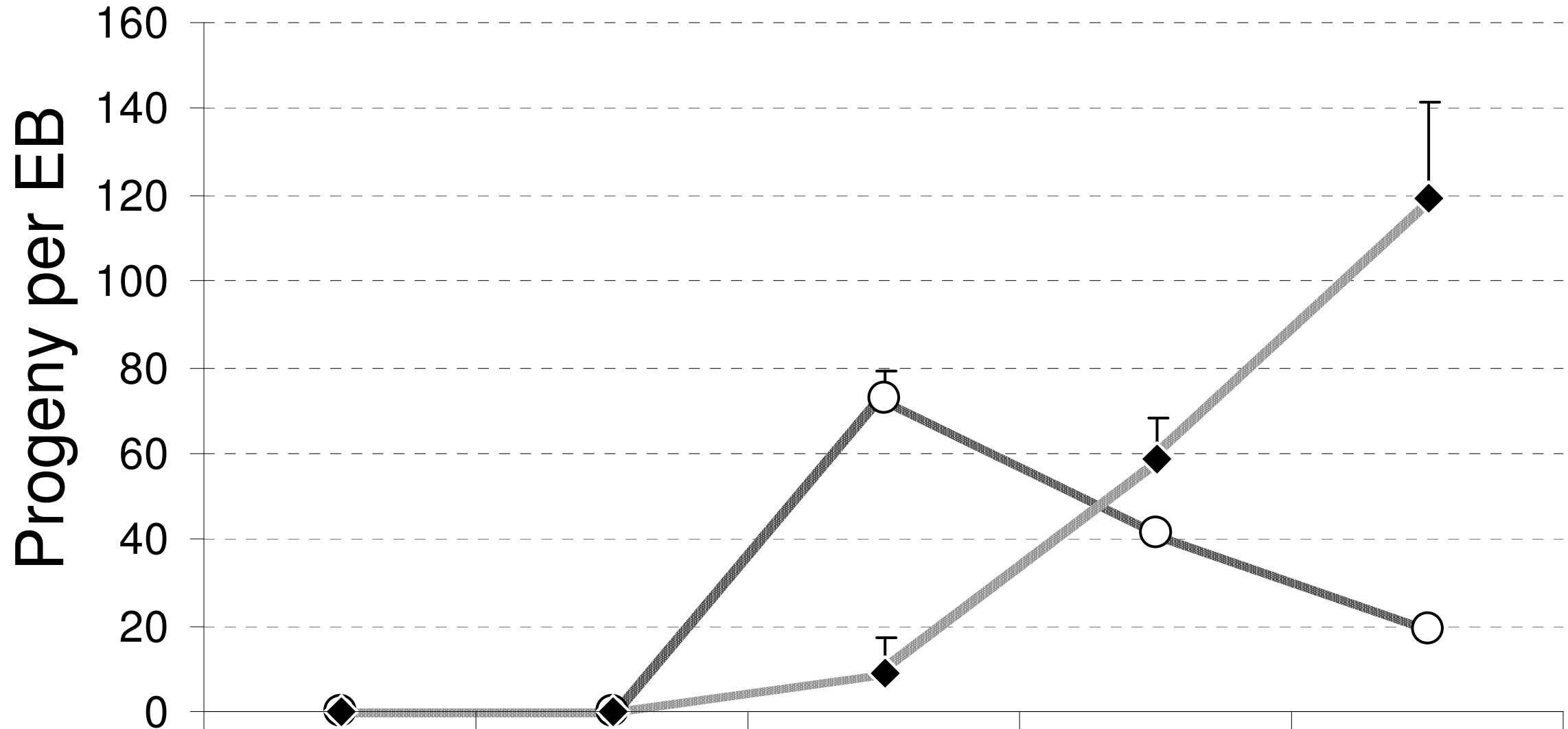

	24 h pi	48 h pi	72 h pi	96 h pi	120 h pi
Cell	0	0	72.84	41.65	19.04
Supernatant	0	0	8.63	58.61	118.90

FIGURE S6
