# Supplementary material for: Gene Expression Profiles of Chlamydophila pneumoniae during the Developmental Cycle and Iron Depletion–Mediated Persistence
Source: PLoS Pathog. 2007 Jun 22;3(6):e83. doi: 10.1371/journal.ppat.0030083 (PMC1894823; doi:10.1371/journal.ppat.0030083)
Supplement: Supplementary file 12 [file ppat.0030083.st001.pdf]

Table S1 – RNA extraction protocol

All materials are to be treated RNAsefree.

(1) Lyse cells with Trizol (1 ml per 1 x 10 ^ 6 cells) and sonicate for 5 to 10 min.
(2) Add 0.2 ml chloroform/ml.
(3) Shake vigorously for 30 s and incubate 5 min at RT.
(4) Centrifuge 15 min at 12000 x g, 4℃.
(5) Transfer aqueous phase into new tube.
(6) Add 0.5 ml isopropyl alcohol, 0.12 ml 5M ammonium acetate,
10 µl of 0.5 mg/µl glycogen. Mix.
(7) Incubate 20 min at -20℃.
(8) Centrifuge 30 min at 12000 x g, 4℃.
(9) Wash pellet with 75% EtOH and resuspend in 85 µl H_2O.
(10) Perform DNAse digest.
(11) Perform phenol/chloroform/isoamyl alcohol (25:24:1) extraction.
(12) Add 0.1 v/v 3M sodium acetate (pH 5.5) and 2.0 v/v EtOH.
(13) Incubate at -20℃ overnight.
(14) Centrifuge 30 min at 12000 x g, 4℃.
(15) Resuspend in appropriate amount of H_2O.
