# Supplementary material for: Gene Expression Profiles of Chlamydophila pneumoniae during the Developmental Cycle and Iron Depletion–Mediated Persistence
Source: PLoS Pathog. 2007 Jun 22;3(6):e83. doi: 10.1371/journal.ppat.0030083 (PMC1894823; doi:10.1371/journal.ppat.0030083)
Supplement: Supplementary file 13 [file ppat.0030083.st002.pdf]

Mäurer et. al 2007

Table S2 – One-Class SAM Calculations for the Acute Infection

Significant: 754

Median number of false positives: 3.5

False Discovery Rate (%): 0.46

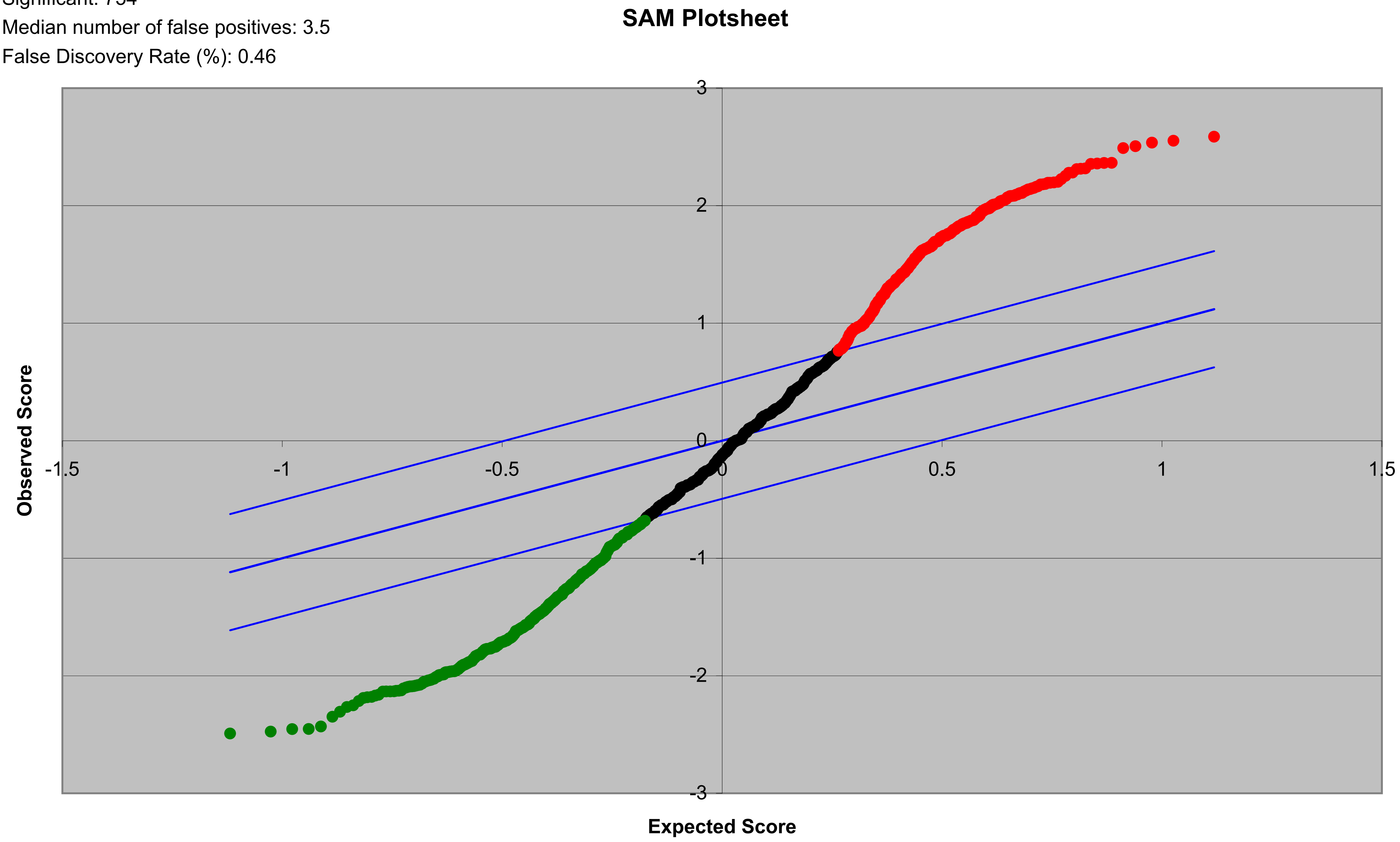

Current settings

Input parameters

**Data type?**	One class timecourse
**Arrays centered?**	FALSCH
**Delta**	0.494663712
**Minimum fold change**	0
**Test statistic**	standard
**Time summary method**	slope
**Are data are log scale?**	FALSCH
**Number of permutations**	100
**Input percentile for exchangeability factor s0**	Automatic choice
**Number of neighbors for KNN**	10
**Seed for Random number generator**	1234567

Computed values

**Estimate of pi0 (proportion of null genes)**	0.218455744
**Exchangibility factor s0**	0.586413417
**s0 percentile**	99.90583804
**False Discovery Rate (%)**	0.463566565

List of Significant Genes for Delta = 0.495

Positive genes (340)

**Gene ID**	**Score(**	**Numerator(r)**	**Denominator(s+s0)**	**q-value(%)**	**localfdr(%)**
CPn0390CP0365ruvB	2.586	1.65489338	0.639934302	0	0.018
CPn0262CP0499surE	2.553	1.68657851	0.660740279	0	0.024
CPn0810CP1061	2.536	1.55924581	0.614894248	0	0.028
CPn0472CP0282	2.506	1.62545122	0.648589655	0	0.033
CPn0669CP0078	2.489	1.51876237	0.610084799	0	0.036
CPn0677CP0070	2.364	1.64370888	0.695170797	0	0.058
CPn0901CP0965murD	2.363	1.54780982	0.655031108	0	0.059
CPn0350CP0409	2.358	1.52072672	0.644953961	0	0.059
CPn0161CP0610	2.355	1.56277386	0.663632903	0	0.06
CPn0384CP0371hctB	2.317	1.68414676	0.727013554	0	0.065
CPn0083CP0692tal	2.314	1.55230794	0.670819635	0	0.065
CPn0497CP0257	2.311	1.51231245	0.654459262	0	0.066
CPn0453CP0299pmp13	2.282	1.53108223	0.67107893	0	0.069
CPn0774CP1098	2.279	1.65448326	0.725855764	0	0.069
CPn0803CP1068	2.253	1.4440507	0.640841097	0	0.071
CPn0711CP0035	2.226	1.41697118	0.636543403	0	0.071
CPn0498CP0256	2.202	1.48722832	0.675410118	0	0.071
CPn0557CP0195omcB	2.198	1.49045093	0.678241582	0	0.071
CPn0852CP1017	2.195	1.71436375	0.78120777	0	0.071
CPn0809CP1062	2.194	1.66928627	0.760761347	0	0.071
CPn0877CP0992ybcL	2.185	1.52017171	0.695887707	0	0.07
CPn0377CP0379sucB1	2.181	1.57097813	0.720138965	0	0.07
CPn0670rsbW	2.18	1.50613045	0.690824153	0	0.07
CPn0705CP0041	2.165	1.42643465	0.658846587	0	0.068
CPn0808CP1063	2.16	1.371096	0.634825607	0	0.068

CPn0322CP0435yscU	2.151	1.44216713	0.670490353	0	0.066
CPn1029CP0823	2.147	1.54613815	0.720051651	0	0.066
CPn0416CP0338himD	2.141	1.35242398	0.631651985	0	0.065
CPn0534CP0218dksA	2.137	1.39175899	0.651214917	0	0.064
CPn0537CP0215	2.128	1.35649072	0.637406347	0	0.062
CPn0389CP0366	2.12	1.60216358	0.755579264	0	0.061
CPn0362CP0395rpsD	2.107	1.38109163	0.655364589	0	0.057
CPn0713CP0033	2.107	1.47344949	0.699275815	0	0.057
CPn0444CP0309pmp6	2.104	1.40590744	0.668350997	0	0.056
CPn0125CP0648	2.095	1.5944161	0.761130951	0	0.053
CPn1023CP0829	2.092	1.45432131	0.695208885	0	0.052
CPn09691yccA1	2.084	1.38165145	0.663062686	0	0.05
CPn0676CP0071	2.083	1.35547348	0.650672139	0	0.049
CPn0781CP1091pal	2.082	1.57099801	0.754454412	0	0.049
CPn0876CP0993dagA2	2.081	1.49790954	0.719815251	0	0.049
CPn0538CP0214	2.074	1.44101823	0.69493856	0	0.046
CPn0581CP0167	2.07	1.37226407	0.663031912	0	0.044
CPn0001CP0775	2.055	1.38740562	0.674976735	0	0.038
CPn0695CP0051ompA	2.049	1.32062754	0.64436656	0	0.035
CPn0822CP1049	2.046	1.39393263	0.681205389	0	0.033
CPn1016CP0837	2.041	1.39484673	0.68331468	0	0.031
CPn0710CP0036	2.041	1.54789794	0.758548788	0	0.03
CPn0421CP0333yabC2	2.036	1.3958635	0.685546201	0	0.028
CPn1047CP0805dapB	2.022	1.4675944	0.725743272	0	0.02
CPn0826CP1045yscL	2.02	1.27703943	0.632318856	0	0.018
CPn0742CP0003	2.015	1.31855125	0.654369193	0	0.015
CPn0604CP0143fliY	2.012	1.39201265	0.691782381	0	0.013
CPn0195CP0572oppA1	2.009	1.66753745	0.830157859	0	0.011
CPn0834CP1035	2.008	1.38988237	0.692242916	0	0.01
CPn0105CP0669	2.004	1.38983937	0.693419395	0	0.008
CPn0355CP0404	1.992	1.32261799	0.664085245	0	0
CPn0796CP1075	1.992	1.26772114	0.636536021	0	0
CPn0828CP1043yscJ	1.978	1.35467346	0.68500406	0	0
Cpn0015_pmp32	1.975	1.31341098	0.664991768	0	0
CPn1033CP0819	1.974	1.28297315	0.649877216	0	0
CPn0700CP0046	1.974	1.24935209	0.633000417	0	0
CPn0702CP0044yscC	1.968	1.38479675	0.703746974	0	0
CPn0445CP0308pmp7	1.965	1.30967124	0.666358813	0	0
CPn0811CP1060lcrH1	1.959	1.48141386	0.756213296	0	0
CPn0193CP0574argR	1.958	1.50655793	0.769365876	0	0
CPn0148CP0625	1.948	1.41048344	0.72422703	0	0
CPn0398CP0357	1.946	1.42263508	0.731227989	0	0
CPn0886CP0980	1.94	1.22611297	0.632075306	0	0
CPn0854CP1015porB	1.918	1.52523723	0.79528802	0	0
Cpn0018_pmp51	1.91	1.3457215	0.704534173	0	0
CPn0863CP1006pgmA	1.908	1.30247468	0.682615266	0	0
CPn0446CP0307pmp8	1.906	1.2221022	0.641242484	0	0
CPn0704fliN	1.905	1.27089671	0.667214173	0	0
CPn1032CP0820	1.891	1.19421145	0.631456172	0	0
CPn0558CP0193	1.885	1.32781203	0.704332863	0	0
CPn0860CP1009fliF	1.877	1.25679561	0.669564905	0	0
CPn0489CP0265	1.876	1.19140154	0.634915786	0	0
CPn0870CP0999serS	1.874	1.31329222	0.700744975	0	0
CPn0608CP0139	1.873	1.33659167	0.713732601	0	0
CPn0985CP0871nrdB	1.87	1.26422607	0.67604743	0	0
CPn0422CP0331	1.867	1.29600233	0.694346444	0	0
CPn1005CP0848	1.865	1.27557314	0.684104318	0	0
CPn0728CP0018	1.861	1.22022822	0.655734357	0	0
CPn0998CP0857ftsH	1.861	1.27763591	0.686612768	0	0
CPn0853CP1016	1.852	1.34769756	0.72750374	0	0
CPn0425CP0328	1.851	1.28910609	0.696328767	0	0
CPn1069CP0781yfgA	1.851	1.29104803	0.697438007	0	0
Cpn0016_pmp41	1.849	1.18562893	0.641172416	0	0

CPn0194CP0573gcp2	1.848	1.31051286	0.709186499	0	0
CPn0540CP0212pmp20	1.842	1.35907057	0.737641737	0	0
CPn0725	1.842	1.33050858	0.72231138	0	0
CPn0139CP0633yqgE	1.838	1.50362794	0.81789213	0	0
CPn0177CP0592	1.835	1.35187787	0.736892112	0	0
CPn0144CP0629clpB	1.829	1.32497867	0.72429354	0	0
CPn0827CP1044	1.828	1.3478518	0.737237772	0	0
Cpn0017_pmp42	1.825	1.30279422	0.713664372	0	0
CPn0709	1.824	1.2726442	0.69771609	0	0
Cpn0014_pmp31	1.818	1.32996109	0.731487746	0	0
CPn0104CP0670	1.814	1.34532478	0.741744008	0	0
CPn0600CP0148	1.81	1.15071681	0.635675819	0	0
CPn0451CP0302pmp11	1.805	1.33869685	0.74170353	0	0
CPn0587CP0161yvyD	1.799	1.36788808	0.76056724	0	0
CPn0204CP0563	1.797	1.43169653	0.7968055	0	0
CPn0707CP0039yscN	1.796	1.27242351	0.70837786	0	0
CPn0365CP0392	1.793	1.24484853	0.694345766	0	0
CPn0661mip	1.783	1.33770321	0.750266007	0	0
CPn0556CP0196crpA	1.781	1.41644531	0.795235064	0	0
CPn0138CP0634hemL	1.771	1.17809457	0.665321066	0	0
CPn0786CP1086dsbD	1.769	1.25590889	0.709830229	0	0
CPn0494CP0260	1.763	1.1437547	0.648775582	0	0
CPn0671CP0076	1.762	1.31400173	0.745930311	0	0
CPn0926CP0940	1.761	1.13235114	0.642974265	0	0
CPn1004CP0849	1.76	1.30229796	0.739907655	0	0
CPn0874CP0995	1.758	1.2760813	0.725796585	0	0
CPn0314CP0444trxB	1.75	1.23900509	0.707844759	0	0
CPn0939CP0922	1.747	1.17912771	0.674995799	0	0
CPn1002CP0851	1.745	1.22937507	0.704378837	0	0
CPn0424CP0329dnaA2	1.744	1.22597014	0.702783446	0	0
CPn0984CP0872nrdA	1.744	1.13531358	0.650933108	0	0
CPn0100CP0674	1.744	1.14764535	0.658232064	0	0
CPn0179CP0589	1.743	1.20154311	0.689382317	0	0
CPn0933CP0928	1.74	1.13044704	0.649824847	0	0
CPn0896CP0970	1.737	1.26555956	0.728389439	0	0
CPn0062CP0713	1.73	1.35631734	0.784138738	0	0
CPn0564CP0186secD/secF	1.728	1.16067696	0.671734964	0	0
CPn0956CP0904	1.728	1.28797707	0.745486819	0	0
CPn0594CP0154pheT	1.727	1.2397078	0.717941315	0	0
CPn0520CP0233clpP1	1.717	1.24130183	0.723128283	0	0
CPn0325CP0432sycE	1.709	1.34456415	0.786634589	0	0
CPn0750CP1122tctD	1.703	1.25212425	0.735351813	0	0
CPn0301CP0457	1.697	1.1614204	0.684509756	0	0
CPn0708CP0038	1.697	1.28435272	0.756990582	0	0
CPn1013CP0840fumC	1.695	1.22474878	0.722365012	0	0
CPn0112CP0661rl31	1.694	1.18055996	0.697007913	0	0
CPn0761CP1111	1.693	1.18468913	0.699574112	0	0
CPn0971CP0888yccA2	1.693	1.14998504	0.679456946	0	0
CPn0535CP0217lspA	1.692	1.27580912	0.754041245	0	0
CPn0423CP0330	1.686	1.23586516	0.733162023	0	0
CPn0872CP0997ribA/ribB	1.681	1.11183014	0.661497022	0	0
CPn1025CP0827pgi	1.679	1.1932681	0.710708346	0	0
CPn0720CP0026	1.674	1.23460662	0.737684456	0	0
CPn0227CP0537dsbB	1.661	1.1879499	0.715386598	0	0
CPn0577CP0171	1.659	1.142119	0.688239982	0	0
CPn0333CP0425	1.659	1.15619531	0.696971631	0	0
CPn0340CP0417	1.651	1.26828031	0.768258789	0	0
CPn0065CP0709	1.65	1.14561424	0.694268686	0	0
CPn0624CP0123gapA	1.649	1.13966149	0.691047529	0	0
CPn0712CP0034	1.646	1.17570253	0.714286218	0	0
CPn0206CP0561	1.642	1.18113999	0.719304661	0	0
CPn0963CP0897pmp21	1.638	1.19566898	0.729915984	0	0
CPn0727CP0019	1.637	1.05740628	0.646040385	0	0

CPn0171CP0599guaA	1.635	1.0288011	0.629144947	0	0
CPn0136CP0636pepF	1.633	1.14670641	0.702021706	0	0
CPn0533CP0219	1.633	1.24281021	0.761018248	0	0
CPn0959CP0901cafE	1.631	1.32995427	0.815484018	0	0
CPn0307CP0451glgP	1.627	1.03251973	0.634771015	0	0
CPn0106CP0668phoH	1.626	1.26263401	0.776550166	0	0
CPn0547CP0205ygbB	1.621	1.25685577	0.775369846	0	0
CPn0703CP0043	1.621	1.44266761	0.8900106	0	0
CPn0930CP0935	1.62	1.10572689	0.682506605	0	0
CPn0140CP0632yqdE	1.619	1.17150231	0.723665687	0	0
CPn0553CP0199	1.612	1.0886319	0.675215249	0	0
CPn0840CP1029	1.612	1.32298629	0.820883294	0	0
CPn0684CP0062parB	1.606	1.1447155	0.712782323	0	0
CPn0894CP0972amn	1.601	1.35184102	0.844211597	0	0
CPn0330CP0427	1.59	1.00507456	0.632279022	0	0
CPn0798CP1073	1.588	1.12971152	0.711274351	0	0
CPn0735CP0011	1.588	1.06299632	0.669481261	0	0
CPn0524CP0229	1.583	1.09517916	0.691963295	0	0
CPn0276CP0483	1.581	1.10836038	0.701166967	0	0
CPn0231CP0533tauB	1.566	1.02017245	0.651282929	0	0
CPn0994CP0861	1.562	1.05340632	0.674198712	0	0
CPn0825CP1046yscR	1.562	1.08120703	0.692191484	0	0
CPn0129CP0642	1.556	0.98490741	0.633108118	0	0
CPn0250CP0511rl33	1.553	1.20298724	0.7746504	0	0
CPn1000CP0854rs15	1.552	1.05909849	0.682358923	0	0
CPn0584CP0164atoS	1.54	1.00771039	0.654494187	0	0
CPn0185CP0583rpe	1.532	1.16359432	0.759574271	0	0
CPn0973CP0886sucC	1.529	1.04308799	0.682137073	0	0
CPn0621CP0126ruvC	1.523	1.22104755	0.801556536	0	0
CPn0546CP0206rl21	1.522	1.09072849	0.716608289	0	0
CPn0324CP0433lcrE	1.519	0.96014122	0.632152015	0	0
CPn0789CP1083sdhA	1.515	1.06247383	0.701115953	0	0
CPn0668	1.501	1.07812207	0.718370388	0	0
CPn0434CP0319	1.5	1.24073033	0.827337945	0	0
CPn0067CP0707	1.498	1.18502005	0.791156343	0	0
CPn0846CP1023clpX	1.49	0.95500193	0.641076352	0	0
CPn0433CP0320gcsH	1.486	1.17863793	0.793021003	0	0
CPn0806CP1065thrS	1.48	0.97273356	0.657445786	0	0
CPn0085CP0690	1.477	1.08787127	0.736689262	0	0
CPn0794CP1077	1.467	1.01652315	0.693129172	0	0
CPn0127CP0645ytfF	1.465	1.05721498	0.721673788	0	0
CPn0667CP0080	1.464	1.07473213	0.734122465	0	0
CPn1028CP0824mdhC	1.461	1.1605074	0.794121107	0	0
CPn0909CP0957rsbV2	1.456	1.01315586	0.695617457	0	0
CPn0094CP0680valS	1.45	1.08184371	0.746224142	0	0
CPn0132CP0640	1.449	1.35863473	0.937374429	0	0
CPn0188CP0579	1.441	1.00107665	0.69450531	0	0
CPn0970CP0889tyrP2	1.433	1.04298932	0.727832547	0	0
CPn0559CP0191	1.432	0.90407748	0.631375261	0	0
CPn0793CP1079rbsU	1.43	1.07046033	0.748682185	0	0
CPn0134CP0638groEL1	1.429	1.14764028	0.803093845	0	0
CPn0369CP0388	1.425	0.96710292	0.678892332	0	0
CPn0652CP0095lpxC	1.421	1.13402494	0.798013488	0	0
CPn0693	1.421	1.06843845	0.751978387	0	0
CPn0103CP0671cydB	1.42	1.07311482	0.755620939	0	0
CPn0467CP0285pmp16	1.415	0.99957937	0.706228887	0	0
CPn0884CP0982	1.408	1.11317741	0.790656122	0	0
CPn0824CP1047yscS	1.406	1.03483839	0.736080525	0	0
CPn0488CP0266ycfF	1.399	1.11493967	0.797032105	0	0
CPn0724CP0022	1.39	1.12086807	0.806113221	0	0
CPn0479CP0275phnP	1.389	1.12853266	0.812200705	0	0
CPn0777CP1095groEL2	1.385	1.00642207	0.726436735	0	0
CPn0595CP0153	1.379	0.93122677	0.67537189	0	0

CPn0057CP0718sodM	1.378	1.01118097	0.733738112	0	0
CPn0399CP0356	1.378	0.94211591	0.683799628	0	0
CPn0912CP0954	1.375	0.89822053	0.653270914	0	0
CPn0047	1.372	1.00700052	0.733940324	0	0
CPn0101CP0673ybbP	1.371	1.10016738	0.802352402	0	0
CPn0769CP1103topA	1.359	1.04435778	0.768685566	0	0
CPn0096CP0678uvrA	1.353	1.03218826	0.762981692	0	0
CPn0847CP1022clpP2	1.351	1.03249077	0.764074273	0	0
CPn0111CP0662	1.342	1.06097896	0.790404917	0	0
CPn0234CP0529	1.342	0.91993967	0.68561205	0	0
CPn0906CP0960	1.338	0.88033102	0.657876026	0	0
CPn0059CP0716dut	1.335	1.06091971	0.794588599	0	0
CPn0437CP0316clpC	1.333	0.99470367	0.746299602	0	0
CPn0447CP0306pmp9	1.333	1.07561362	0.807177196	0	0
CPn0135CP0637groES	1.326	0.95819069	0.722777304	0	0
CPn0965CP0895lpxB	1.322	0.99509753	0.752787936	0	0
CPn0521CP0232glyA	1.322	1.02515092	0.77572237	0	0
CPn0228CP0536dsbG	1.321	1.1416085	0.863982685	0	0
CPn0782CP1090tolB	1.31	1.05384355	0.804375399	0	0
CPn0717CP0029	1.31	1.34277624	1.025045499	0	0
CPn0850CP1019mreB	1.301	1.02629828	0.788874396	0	0
CPn0865CP1004	1.3	0.95633891	0.735575896	0	0
CPn0323CP0434lcrD	1.299	1.08887031	0.837956346	0	0
CPn0183CP0585accB	1.299	0.99749392	0.76818853	0	0
CPn0905CP0961murC/ddlA	1.295	0.92495135	0.714454549	0	0
CPn0415CP0339	1.279	1.06342204	0.831381511	0	0
CPn1034CP0818	1.277	1.04844291	0.821292026	0	0
CPn0616CP0131dnaB	1.274	1.02520565	0.804525647	0	0
CPn0678	1.264	0.90115703	0.712791971	0	0
CPn0012CP0762	1.26	0.91794237	0.728253165	0	0
CPn0493CP0261	1.246	0.85362015	0.684948388	0	0
CPn1001CP0852yfhC	1.244	1.06742451	0.857910028	0	0
CPn0295CP0463	1.242	0.98507921	0.792844761	0	0
CPn0126CP0646	1.242	0.98452981	0.792843585	0	0
CPn0778CP1094ahpC	1.236	0.92607148	0.749331592	0	0
CPn0940CP0921uvrC	1.233	0.87347589	0.708432167	0	0
CPn0263CP0498yqfU	1.227	0.84058594	0.685181044	0	0
CPn0209CP0557	1.226	0.89742508	0.73186443	0	0
CPn0974CP0885sucD	1.217	0.86623005	0.711558009	0	0
CPn0260CP0501secA1	1.202	1.04391854	0.868161169	0	0
CPn0978CP0878	1.194	0.93637883	0.78433774	0	0
CPn0726	1.194	0.82933084	0.69471643	0	0
CPn0056CP0719mrsA	1.189	0.79176664	0.665667167	0	0
CPn0490CP0264	1.186	0.99247057	0.83651978	0	0
CPn0762CP1110	1.184	0.91505074	0.7728651	0	0
CPn0849CP1020	1.166	0.86948955	0.745624182	0.035292	0
CPn1049CP0803lysC	1.162	0.77950675	0.670952283	0.035292	0
CPn0832CP1038lipA	1.161	0.86370816	0.743896653	0.035292	0
CPn0040CP0735dnaX1	1.16	0.91402841	0.78778705	0.035292	0
CPn0659CP0088	1.152	1.04551753	0.907434977	0.035292	0
CPn0913CP0953	1.141	0.88444578	0.77500594	0.035292	0
CPn0189CP0578	1.126	0.77122233	0.684674169	0.035292	0
CPn0178CP0590	1.115	0.72435066	0.649811858	0.035292	0
CPn0586CP0162atoC	1.11	0.88106432	0.793694076	0.035292	0
CPn0164CP0607	1.099	0.82935191	0.754720603	0.035292	0.066
CPn0402CP0353mutY	1.097	0.82730106	0.754145279	0.035292	0.1
CPn0304CP0454pdhA	1.092	0.85714123	0.784703768	0.035292	0.186
CPn0473CP0281	1.084	0.86597941	0.799223396	0.035292	0.354
CPn0419CP0335pbp3	1.08	0.87236377	0.807619379	0.035292	0.421
CPn0004CP0771gatB	1.074	0.76064942	0.708539755	0.035292	0.558
CPn0701CP0045karG	1.071	0.90894038	0.848976526	0.035292	0.62
CPn0143CP0630yxjG1	1.054	0.94832343	0.900011202	0.035292	1.004
CPn0256CP0505	1.05	0.78323394	0.745706837	0.035292	1.085

CPn0622CP0125	1.045	0.72416971	0.693119877	0.035292	1.222
CPn0216CP0549	1.041	0.92184472	0.885864241	0.035292	1.329
CPn0409CP0345	1.036	0.84222007	0.81306949	0.035292	1.453
CPn0873CP0996ribE	1.029	0.81167325	0.788775706	0.035292	1.638
CPn0349CP0411ytgA	1.028	0.71256642	0.693494458	0.035292	1.68
CPn0936CP0925rl36	1.027	0.91379901	0.889920766	0.035292	1.699
CPn0200CP0567oppC1	1.022	0.81257163	0.795315367	0.068589	1.845
CPn0275CP0484gyrB1	1.014	0.77738756	0.766551533	0.068589	2.067
CPn0448CP0305yxjG2	1.012	0.76481709	0.756041707	0.068589	2.143
CPn0153CP0618leuS	0.995	0.74894534	0.752431955	0.068589	2.658
CPn0770CP1102	0.995	0.78771843	0.791393035	0.068589	2.659
CPn0441CP0312	0.993	0.84113417	0.846783683	0.068589	2.726
CPn0190CP0577	0.992	0.7711097	0.777245414	0.068589	2.767
CPn0857CP1012	0.992	0.82192803	0.828932585	0.068589	2.786
CPn0184CP0584efp1	0.977	0.84179838	0.861391415	0.068589	3.287
CPn0413CP0341msbA	0.976	0.76786925	0.787035369	0.068589	3.345
CPn0833CP1037	0.975	0.8985576	0.922004399	0.068589	3.385
CPn0160CP0611	0.974	0.96231238	0.988014119	0.068589	3.406
CPn0338CP0419dnaN	0.972	0.73349519	0.754898693	0.068589	3.493
CPn0337CP0421smpB	0.971	0.93298389	0.960983876	0.068589	3.522
CPn1031CP0821arcD	0.97	0.67650261	0.697205416	0.068589	3.543
CPn0232CP0532	0.969	0.67811675	0.699482576	0.068589	3.575
CPn0902CP0964nlpD	0.967	0.79929484	0.826604913	0.082623	3.669
CPn0110CP0664lepB	0.965	0.80886916	0.838318266	0.082623	3.75
CPn0207CP0560ybhI	0.958	0.75740906	0.790453384	0.082623	4.011
CPn0048CP0726yqfF	0.958	0.79433165	0.829027071	0.082623	4.013
CPn1006CP0847	0.957	0.70703304	0.738944023	0.082623	4.066
CPn0300CP0458yaeT	0.953	0.74237878	0.778727391	0.082623	4.206
CPn0800CP1071eno	0.953	0.65839719	0.690905736	0.082623	4.222
CPn0332	0.953	0.78894548	0.827942597	0.082623	4.224
CPn0791CP1081	0.947	0.69020711	0.728712066	0.082623	4.461
CPn0427CP0326nqr2	0.943	0.84809488	0.899690831	0.082623	4.651
Cpn0061_CP0714	0.938	0.6850735	0.730379984	0.129264	4.853
CPn0754CP1118rs20	0.936	0.72554941	0.775421048	0.129264	4.953
CPn0172guaB	0.931	0.66873262	0.718315897	0.129264	5.162
CPn0297CP0461fabD	0.93	0.81879412	0.88032642	0.129264	5.201
CPn0387CP0368	0.92	0.68092196	0.740169415	0.129264	5.669
CPn0797CP1074	0.913	0.90007744	0.985516624	0.143931	5.987
CPn0899CP0967murF	0.908	0.79203748	0.871945335	0.143931	6.229
CPn0721kdsA	0.905	0.65841661	0.727253573	0.143931	6.379
CPn0466CP0286pmp15	0.9	0.72173119	0.801578472	0.143931	6.63
CPn0607CP0140glgC	0.877	0.64626579	0.737096203	0.188867	7.895
CPn0525CP0228	0.877	0.71206117	0.812333606	0.188867	7.906
CPn0286CP0472mgtE	0.857	0.81325001	0.949292819	0.217215	9.064
CPn0687CP0059	0.847	0.71938871	0.849439937	0.28824	9.667
CPn0248CP0513ycfV	0.842	0.71653429	0.850508622	0.28824	9.945
CPn0142	0.84	0.68305792	0.813260231	0.28824	10.11
CPn0133CP0639	0.839	0.76775159	0.914879388	0.28824	10.16
CPn1070CP0780	0.825	0.79936892	0.968853195	0.358124	11.09
CPn0309CP0449dnaA1	0.818	0.71912143	0.879624193	0.358124	11.6
CPn0921CP0945	0.812	0.60077218	0.739441989	0.358124	11.96
CPn0785CP1087exbB	0.812	0.79732093	0.98201177	0.358124	11.99
CPn0229CP0535	0.806	0.70080194	0.869103024	0.358124	12.39
CPn0383CP0372	0.796	0.72199754	0.906755832	0.426901	13.13
CPn0282CP0476xasA	0.796	0.76278299	0.958762825	0.426901	13.18
CPn0889CP0977hemN2	0.786	0.54544019	0.694094066	0.426901	13.91
CPn0154CP0617gseA	0.782	0.68579789	0.876697856	0.426901	14.19
CPn0254CP0507	0.782	0.74879355	0.957949381	0.426901	14.23
CPn0495CP0259aspC	0.78	0.69377625	0.889912863	0.426901	14.4
CPn0401CP0354	0.779	0.58135903	0.746320076	0.426901	14.44
CPn0044CP0729	0.775	0.6686049	0.863228246	0.463567	14.79
CPn0162CP0609	0.773	0.66365006	0.858393276	0.463567	14.91
CPn0571CP0178murA	0.765	0.70738776	0.924613449	0.463567	15.56

	Negative genes (414)				
**Gene ID**	**Score(**	**Numerator(r)**	**Denominator(s+s0)**	**q-value(%)**	**localfdr(%)**
CPj0954Cpn0954	-2.49	-1.57642247	0.632844958	0	0.037
CPn0682CP0065dppD	-2.48	-1.64467432	0.664507932	0	0.041
CPn0439CP0314	-2.45	-1.57604914	0.642245088	0	0.045
CPn0046CP0728	-2.45	-1.59712579	0.651067946	0	0.045
CPn0477CP0277yqeV	-2.43	-1.50588736	0.61914363	0	0.049
CPn0372CP0385	-2.35	-1.6249558	0.691921025	0	0.065
CPn0617CP0130gidA	-2.31	-1.56592411	0.678889094	0	0.072
CPn0396CP0359yhfO	-2.27	-1.46077081	0.644628615	0	0.078
CPn0119CP0654rnhB1	-2.25	-1.50927856	0.670127124	0	0.08
CPn0509CP0245	-2.22	-1.5017047	0.677813007	0	0.084
CPn0117CP0656trmD	-2.19	-1.38416222	0.632375998	0	0.085
CPn0544CP0208yhbZ	-2.18	-1.56624116	0.717518415	0	0.085
CPn1045CP0807	-2.18	-1.44599476	0.663401478	0	0.085
CPn0261CP0500ydaO	-2.17	-1.53714842	0.708632468	0	0.085
CPn0543CP0209	-2.16	-1.42075636	0.657082672	0	0.085
CPn0730CP0016mviN	-2.13	-1.34560753	0.630479622	0	0.083
Cpn0548_CP0204	-2.13	-1.31737426	0.617367445	0	0.083
CPn0881CP0986	-2.13	-1.41149559	0.661537197	0	0.083
CPn0579CP0169yacM	-2.13	-1.44766556	0.678803663	0	0.083
CPn0550fusA	-2.13	-1.39327416	0.654614166	0	0.083
CPn0689CP0057yfhO1	-2.13	-1.83132893	0.860772647	0	0.083
CPn0527CP0225sucB2	-2.12	-1.45925695	0.686786097	0	0.082
CPn0536CP0216dagA1	-2.11	-1.43442381	0.680444377	0	0.08
CPn0066CP0708	-2.1	-1.33483	0.635651492	0	0.079
CPn0580CP0168truA	-2.1	-1.41535417	0.675515128	0	0.078
CPn0268CP0492	-2.09	-1.38722888	0.663452765	0	0.077
CPn0961CP0899rl32	-2.09	-1.54854155	0.740860439	0	0.077
CPn0585CP0163	-2.09	-1.33145435	0.637557848	0	0.076
CPn0766CP1106	-2.08	-1.38619151	0.665108651	0	0.076
CPn0240CP0522	-2.08	-1.30946213	0.629596801	0	0.074
CPn0597CP0151oppC2	-2.08	-1.35513619	0.652467743	0	0.074
CPn0648CP0099	-2.07	-1.45109938	0.702042286	0	0.071
CPn0024CP0752xerC	-2.05	-1.37044087	0.667602733	0	0.066
CPn0596CP0152ada	-2.05	-1.47830457	0.721015517	0	0.066
CPn0554CP0198	-2.05	-1.40429201	0.686367702	0	0.064
CPn0113CP0660pfrA	-2.04	-1.56733582	0.767925956	0	0.062
CPn1010CP0843	-2.04	-1.47289804	0.722912063	0	0.061
CPn0960CP0900	-2.03	-1.41613477	0.696023581	0	0.059
CPn0028CP0748	-2.03	-1.34348489	0.662261573	0	0.057
CPn0035CP0741	-2.03	-1.4037391	0.69308821	0	0.055
CPn0759CP1113folA	-2.01	-1.29837971	0.644493223	0	0.05
CPn0492CP0262	-2.01	-1.34632694	0.670370692	0	0.047
CPn0465CP0287	-2.01	-1.41681681	0.706149021	0	0.046
CPn1059CP0791kgsA	-2	-1.29132081	0.647092204	0	0.039
CPn0266CP0495	-1.99	-1.32387012	0.66423247	0	0.038
CPn0958CP0902plsB	-1.99	-1.40529031	0.705453329	0	0.037
CPn0515CP0238ubiE	-1.99	-1.35243246	0.679769028	0	0.036
CPn0348CP0412ytgB1	-1.99	-1.42110023	0.714594219	0	0.035
CPn1027CP0825	-1.97	-1.35073617	0.684333534	0	0.025
CPn0464CP0288	-1.97	-1.37461933	0.697732286	0	0.022
CPn0614CP0133adt2	-1.97	-1.48484403	0.753908862	0	0.022
CPn0397CP0358	-1.97	-1.37915797	0.700637298	0	0.021
CPn0476CP0278	-1.97	-1.21826428	0.619476166	0	0.02
CPn0025CP0751atsA	-1.96	-1.33626907	0.68041908	0	0.018
CPn0284CP0474	-1.96	-1.34388013	0.684490808	0	0.017
CPn0522CP0231	-1.96	-1.34561854	0.685992955	0	0.016
CPn0237CP0525yggF	-1.96	-1.59712129	0.81431584	0	0.016
CPn0356CP0402	-1.96	-1.29094897	0.658218748	0	0.016
CPn0629CP0118secY	-1.96	-1.42148766	0.726413673	0	0.012

CPn0102CP0672cydA	-1.95	-1.36274453	0.697798008	0	0.009
CPn0560CP0190gltX	-1.95	-1.24837073	0.641106524	0	0.004
CPn0223CP0542	-1.94	-1.42972552	0.73578552	0	1E-04
CPn0790CP1082sdhB	-1.93	-1.27368997	0.658487592	0	0
CPn1011	-1.93	-1.21033594	0.627642899	0	0
CPn0816CP1055gspE	-1.92	-1.46558914	0.762095165	0	0
CPn0868CP1001zntA	-1.91	-1.43717706	0.750568921	0	0
CPn0023CP0753yjjK	-1.91	-1.35546395	0.70864049	0	0
CPn0003CP0772gatA	-1.91	-1.22179191	0.640887979	0	0
CPn0848CP1021tigA	-1.91	-1.17689642	0.617398974	0	0
CPn0031CP0745rs21	-1.9	-1.18422965	0.622219212	0	0
CPn0632CP0115rl18	-1.9	-1.2460381	0.656417722	0	0
CPn0149CP0624dnlJ	-1.89	-1.31709236	0.695266522	0	0
CPn0081CP0694rpoB	-1.89	-1.19721348	0.632890444	0	0
CPn0120CP0653gmk	-1.89	-1.34455241	0.712885612	0	0
Cpn0090_CP0684	-1.88	-1.45499478	0.772131285	0	0
CPn1063CP0786tpiS	-1.88	-1.28531251	0.683841556	0	0
CPn0882CP0984	-1.88	-1.26819095	0.675938529	0	0
CPn0442CP0311	-1.87	-1.35646524	0.723606307	0	0
CPn0115CP0658ffh	-1.86	-1.20268223	0.644983123	0	0
CPn0763CP1109ygfA	-1.86	-1.13415292	0.61109126	0	0
CPn0801CP1070	-1.85	-1.25162377	0.676312756	0	0
CPn0618CP0129lplA2	-1.85	-1.19862643	0.649065578	0	0
CPn0593CP0155	-1.83	-1.34409854	0.733000872	0	0
CPn0598CP0150oppB2	-1.83	-1.36599396	0.746416547	0	0
CPn0486CP0268	-1.83	-1.33936462	0.732528696	0	0
CPn0418CP0336murE	-1.82	-1.21885018	0.668684298	0	0
CPn0036CP0739	-1.82	-1.31826942	0.723849461	0	0
CPn0838CP1031thdF	-1.82	-1.51514436	0.83216753	0	0
CPn0267CP0493	-1.82	-1.40274711	0.770566013	0	0
CPn0734yceA	-1.81	-1.14574441	0.632100804	0	0
CPn0839CP1030psdD	-1.81	-1.28927372	0.713067407	0	0
CPn0030CP0746gcp1	-1.8	-1.22121518	0.677485513	0	0
CPn0662CP0085aspS	-1.8	-1.31400052	0.73177378	0	0
CPn0367CP0390	-1.79	-1.21219856	0.676189269	0	0
CPn0431CP0322	-1.79	-1.25616401	0.702165984	0	0
CPn0690CP0056	-1.78	-1.2789929	0.718652372	0	0
CPn0633CP0114rl6	-1.78	-1.36801556	0.770363693	0	0
CPn0674CP0073fmu	-1.77	-1.15121023	0.648641902	0	0
CPn0410CP0344dnaQ1	-1.77	-1.2736709	0.718697046	0	0
CPn0788CP1084sdhC	-1.77	-1.22195099	0.689873722	0	0
CPn0380CP0376hemN1	-1.77	-1.19864283	0.676715006	0	0
CPn0787CP1085yabD	-1.77	-1.25767099	0.710093023	0	0
CPn0480CP0274	-1.77	-1.15736852	0.653950867	0	0
CPn0505CP0248	-1.77	-1.33709562	0.755955398	0	0
CPn0285CP0473	-1.77	-1.30250655	0.736440865	0	0
CPn0862CP1007yfhO2	-1.76	-1.15297382	0.65440064	0	0
CPn0765CP1107	-1.76	-1.15949612	0.658466364	0	0
CPn0272CP0487dnaX2	-1.76	-1.18658909	0.674828822	0	0
CPn0928CP0938	-1.76	-1.16996304	0.665582453	0	0
CPn0976CP0880	-1.75	-1.14548437	0.65271968	0	0
CPn0079CP0696rl10	-1.75	-1.1218163	0.639254217	0	0
CPn0238CP0524zwf	-1.75	-1.1589877	0.660994648	0	0
CPn0715CP0031gyrB2	-1.75	-1.32044714	0.753198142	0	0
CPn0992CP0863	-1.75	-1.27581816	0.730797937	0	0
CPn0146CP0627	-1.74	-1.21807365	0.698831546	0	0
CPn0087CP0687	-1.74	-1.15582546	0.66372446	0	0
CPn0236CP0526pyrG	-1.74	-1.23697692	0.711864078	0	0
CPn0871CP0998ribD	-1.73	-1.17197685	0.678073784	0	0
CPn0741CP0004	-1.73	-1.23387925	0.714168043	0	0
CPn0230CP0534	-1.72	-1.17499713	0.681407271	0	0
CPn0440CP0313	-1.72	-1.45746646	0.84869985	0	0
CPn0251CP0510	-1.72	-1.36916195	0.797427256	0	0

CPn0032CP0744dnaJ	-1.72	-1.12846096	0.657874726	0	0
CPn0218CP0547	-1.71	-1.24748472	0.728228528	0	0
CPn0649CP0098fmt	-1.71	-1.4276794	0.833427682	0	0
CPn0783CP1089	-1.71	-1.37319565	0.802540895	0	0
CPn0116CP0657rs16	-1.71	-1.1439805	0.668986684	0	0
CPn0620CP0127ruvA	-1.71	-1.29301027	0.756985061	0	0
CPn0091CP0683atpI	-1.7	-1.11544031	0.654967002	0	0
CPn0076CP0699nusG	-1.7	-1.37929511	0.810055531	0	0
CPn1018CP0835	-1.7	-1.10742261	0.650868056	0	0
CPn0831CP1039	-1.7	-1.0826341	0.636327577	0	0
CPn0947CP0912pgsA2	-1.7	-1.24351038	0.731856924	0	0
CPn0058CP0717accD	-1.69	-1.15976128	0.685479004	0	0
CPn0644CP0103rl2	-1.69	-1.14209886	0.675585002	0	0
CPn0523CP0230	-1.69	-1.28660855	0.762948675	0	0
CPn0631CP0116rs5	-1.68	-1.08612931	0.646045424	0	0
CPn0837CP1032nth	-1.68	-1.13762654	0.676840661	0	0
CPn0080CP0695rl7	-1.68	-1.19043662	0.708624153	0	0
CPn0655CP0092dnaQ2	-1.68	-1.08887901	0.64949828	0	0
CPn0086_CP0688	-1.67	-1.21475748	0.725885121	0	0
CPn0615CP0132pgsA1	-1.67	-1.12704676	0.674093201	0	0
CPn0187CP0580	-1.66	-1.11799903	0.671644601	0	0
CPn1040CP0812	-1.66	-1.16831532	0.702639188	0	0
CPn1073CP0776	-1.65	-1.21611122	0.73545204	0	0
CPn0910CP0956miaA	-1.65	-1.19967664	0.726732915	0	0
CPn0772CP1100uvrD	-1.64	-1.1442508	0.696189459	0	0
CPn0298CP0460fabH	-1.63	-1.07393907	0.657814675	0	0
CPn0026CP0750	-1.62	-1.16269294	0.717901335	0	0
CPn0456	-1.62	-1.10244847	0.680845439	0	0
CPn0006	-1.62	-1.32841819	0.821033667	0	0
CPn0088CP0686atpA	-1.61	-1.12724016	0.698039682	0	0
CPn0412CP0342	-1.61	-1.28632695	0.796859644	0	0
CPn0168CP0603	-1.61	-1.22915097	0.762541725	0	0
CPn0627CP0120rs11	-1.61	-1.19597279	0.744250247	0	0
CPn0020CP0756	-1.61	-1.26330005	0.786887914	0	0
CPn0318CP0439rbfA	-1.6	-1.35094767	0.843113286	0	0
CPn0679CP0068	-1.6	-1.03046665	0.644698951	0	0
CPn0429CP0324nqr4	-1.6	-1.05608073	0.661152051	0	0
CPn0542CP0210	-1.6	-1.26191261	0.791103652	0	0
CPn0118CP0655rl19	-1.59	-1.40961144	0.885816559	0	0
CPn0281CP0477dhnA	-1.59	-1.1824822	0.743212324	0	0
CPn0966CP0894pcnB2	-1.59	-1.29809043	0.816112821	0	0
CPn0514CP0239	-1.59	-1.21640082	0.76617996	0	0
CPn0320CP0437ribF	-1.58	-1.35825949	0.858669794	0	0
CPn0152CP0620	-1.58	-1.15027279	0.727549149	0	0
CPn0043CP0730	-1.58	-1.31771394	0.834572782	0	0
CPn1053CP0799	-1.57	-1.13693399	0.723157243	0	0
CPn0381CP0375	-1.57	-0.99111877	0.632154641	0	0
CPn0844CP1025yphC	-1.57	-1.31026258	0.836105686	0	0
CPn0835CP1034	-1.57	-1.21934684	0.778501432	0	0
CPn0953CP0906rl9	-1.56	-1.23162317	0.787346826	0	0
CPn0654CP0093yciA	-1.56	-1.0978016	0.701825336	0	0
CPn0241CP0521	-1.56	-1.05753442	0.676353806	0	0
CPn0198CP0569oppA4	-1.56	-1.13204888	0.726827732	0	0
CPn0432CP0321	-1.55	-1.21580276	0.781935374	0	0
CPn0529CP0223ycaH	-1.54	-1.14475752	0.743022529	0	0
CPn0551_rs7	-1.54	-1.13703708	0.739954909	0	0
CPn0182CP0586accC	-1.53	-1.0603418	0.691057277	0	0
CPn0719CP0027sfhB	-1.53	-1.06437703	0.695369191	0	0
CPn0075CP0700secE	-1.53	-1.02453462	0.670778773	0	0
CPn0452CP0301pmp12	-1.53	-1.19261434	0.781201933	0	0
CPn0927CP0939	-1.52	-1.19895591	0.787580973	0	0
CPn0541CP0211	-1.52	-1.002417	0.659731031	0	0
CPn0924CP0942priA	-1.52	-1.31702454	0.866838917	0	0

CPn0563CP0187recJ	-1.51	-1.03572334	0.683685198	0	0
CPn0050	-1.51	-1.01877612	0.673355445	0	0
CPn0836CP1033brnQ	-1.5	-0.98608585	0.655961983	0	0
CPn0400CP0355	-1.5	-1.1082318	0.739545056	0	0
CPn0628CP0119rs13	-1.5	-1.18447832	0.791071267	0	0
CPn1012CP0841yzeB	-1.49	-1.01072668	0.677270307	0	0
CPN03681_CP03891	-1.49	-1.19486941	0.801145382	0	0
CPn0499CP0255	-1.49	-1.01955766	0.685978075	0	0
CPn0328CP0429	-1.48	-1.01603707	0.684471412	0	0
CPn0993CP0862pheS	-1.48	-1.12053836	0.755762386	0	0
CPn0929CP0937	-1.48	-1.0869104	0.73448804	0	0
CPn0972CP0887ftsY	-1.48	-1.08441001	0.733038634	0	0
CPn0878CP0991	-1.47	-1.00241853	0.680015214	0	0
CPn0944CP0915	-1.47	-1.03963087	0.705309543	0	0
CPn0549CP0203rs10	-1.47	-1.08302075	0.734796331	0	0
CPn0673CP0074	-1.47	-1.20150827	0.818581833	0	0
CPn0312CP0446	-1.47	-1.02047466	0.695967601	0	0
CPn0077CP0698rl11	-1.46	-1.06301182	0.72696286	0	0
CPn0646CP0101rl4	-1.46	-1.10290508	0.754925162	0	0
CPn0625CP0122rl17	-1.46	-0.99992542	0.686218671	0	0
CPn0212CP0553	-1.46	-1.0131934	0.695722112	0	0
CPn0957CP0903ide	-1.45	-1.04526343	0.720278805	0	0
CPn1021CP0832lcrH2	-1.45	-1.01667971	0.700869542	0	0
CPn0491CP0263	-1.45	-1.10554354	0.763201332	0	0
CPn0008CP0766	-1.44	-1.2607451	0.872826186	0	0
CPn0336CP0422yojL	-1.44	-0.96296563	0.668000233	0	0
CPn0089CP0685atpB	-1.44	-1.08093735	0.752668532	0	0
CPn0739CP0006	-1.43	-1.00546756	0.703895555	0	0
CPn0641CP0106rs3	-1.43	-1.0416166	0.729964011	0	0
CPn0038CP0737ptsI	-1.42	-0.97847361	0.686835086	0	0
CPn0359CP0399lepA	-1.42	-1.19256563	0.837908876	0	0
CPn0950CP0909pth	-1.42	-1.2097365	0.854470114	0	0
CPn0574CP0175	-1.41	-0.97532846	0.689504129	0	0
CPn0991CP0864rl35	-1.41	-1.04089097	0.740578271	0	0
CPn0403yceC	-1.4	-1.06948378	0.76167378	0	0
CPn0723yhbG	-1.4	-0.98497335	0.702285315	0	0
CPn0093CP0681	-1.39	-0.91727661	0.659572762	0	0
CPn0952CP0907rs18	-1.39	-0.95049807	0.683866469	0	0
CPn0605CP0142yhhF	-1.39	-1.00160551	0.722494741	0	0
CPn0983CP0873	-1.38	-0.86897861	0.628170618	0	0
CPn0843CP1026	-1.38	-1.10853147	0.801779285	0	0
CPn0915CP0951ybeB	-1.38	-1.0867657	0.786156282	0	0
CPn0635CP0112rl5	-1.38	-1.04404522	0.758494212	0	0
CPn0518CP0235	-1.37	-0.94749426	0.690499215	0	0
CPn0539CP0213pmp19	-1.37	-1.04453548	0.762564789	0	0
CPn0145CP0628	-1.37	-1.11047043	0.811821597	0	0
CPn0588CP0160	-1.37	-0.90896206	0.664855809	0	0
CPn0121CP0652	-1.36	-0.93773826	0.689761658	0	0
CPn0737CP0008recC	-1.36	-1.05423315	0.776422576	0	0
CPn0327CP0430rl28	-1.36	-1.11319629	0.820698943	0	0
CPj0339Cpn0339	-1.35	-1.1304998	0.839067705	0	0
CPn0634CP0113rs8	-1.35	-1.11059203	0.824663705	0	0
CPn0353CP0406	-1.34	-0.91507182	0.681341541	0	0
CPn1060CP0790tktB2	-1.34	-0.88521986	0.662754922	0	0
CPn0379CP0377	-1.33	-1.10152079	0.827638049	0	0
CPn1051CP0801	-1.33	-1.04104562	0.782586079	0	0
CPn0287CP0471	-1.33	-0.91737362	0.690345271	0	0
CPn0784CP1088exbD	-1.33	-0.96236415	0.725556688	0	0
CPn0078CP0697rl1	-1.32	-0.99209437	0.749164934	0	0
CPn0150CP0623	-1.32	-1.07133653	0.810263992	0	0
CPn0916CP0950fabF	-1.32	-1.03137777	0.781315899	0	0
CPn0288CP0470	-1.32	-1.11220653	0.84563701	0	0
CPn0817CP1054gspF	-1.31	-1.02861384	0.7837816	0	0

CPn0055CP0720	-1.31	-1.19291023	0.909474313	0	0
CPn0945CP0914	-1.31	-0.90359323	0.69018776	0	0
CPn0211CP0554	-1.31	-1.01473407	0.7753404	0	0
CPn0569CP0180plsC	-1.29	-0.99843503	0.771585911	0	0
CPn0692CP0054	-1.29	-0.88348843	0.684334846	0	0
CPn0650CP0097lpxA	-1.28	-0.90124549	0.704196013	0	0
CPn0815CP1056gspD	-1.28	-0.95765518	0.748306593	0	0
CPn0890CP0976hemE	-1.28	-0.96857923	0.758456562	0	0
CPn0516CP0237	-1.27	-0.92211631	0.723711397	0	0
CPn0406CP0349	-1.27	-1.02339635	0.807348252	0	0
CPn0201CP0566oppD	-1.26	-1.04408486	0.826851799	0	0
CPn0481	-1.26	-0.84236034	0.667125482	0	0
CPn0613CP0134sohB	-1.26	-0.83003247	0.659905263	0	0
CPn0997CP0858mesJ	-1.26	-0.96087948	0.764016234	0	0
CPn0880CP0989ftsK	-1.26	-0.89902417	0.714993007	0	0
CPn0565CP0185	-1.26	-0.91624097	0.72872834	0	0
CPn0980CP0876	-1.25	-0.9888713	0.789558176	0	0
CPn0892CP0974alaS	-1.25	-0.86014732	0.688606748	0	0
CPn0049CP0725	-1.24	-0.94394455	0.76182642	0	0
CPn0289CP0469	-1.23	-0.8178022	0.662598011	0	0
CPn1022CP0831	-1.23	-0.90961678	0.741033885	0	0
CPn0821CP1050	-1.22	-0.98945108	0.808044858	0	0
CPn0346CP0414ytgD	-1.22	-0.85606375	0.702573924	0	0
CPn0526CP0226kpsF	-1.22	-1.04093751	0.854771154	0	0
CPn0570CP0179	-1.22	-0.97451104	0.801203912	0	0
CPn0572CP0177	-1.22	-0.82644692	0.6797658	0	0
CPn0962CP0898plsX	-1.21	-0.89213401	0.735350665	0	0
CPn0461CP0291	-1.21	-0.87930854	0.727220509	0	0
CPn1035CP0817aroE	-1.2	-0.93179495	0.778207881	0	0
CPn0975CP0882	-1.2	-0.95077805	0.794118875	0	0
CPn0504CP0249vacB	-1.19	-0.93977334	0.790490768	0	0
CPn0411CP0343	-1.19	-0.87890565	0.740529249	0	0
CPn0823CP1048yscT	-1.18	-1.02990775	0.870129036	0	0
CPn0095CP0679	-1.18	-0.87681875	0.741735704	0	0
CPn0917CP0949	-1.18	-0.79755095	0.677850116	0	0
CPn0562CP0188	-1.17	-0.91519823	0.779909417	0	0
CPn0436CP0317	-1.17	-0.85838472	0.731901813	0	0
CPn0716CP0030	-1.17	-0.87187595	0.745674112	0	0
CPn0470pmp173	-1.16	-0.99721297	0.858536961	0	0
CPn1072CP0777	-1.16	-0.83072408	0.716355185	0	0
CPn0645CP0102rl23	-1.15	-0.92084492	0.797501759	0	0
CPn0219CP0546tgt	-1.15	-0.8413938	0.733732451	0	0
CPn0351CP0408adt1	-1.14	-0.73886274	0.649725282	0	0
CPn0197CP0570oppA3	-1.14	-1.03658032	0.912422864	0	0
CPn0054CP0721rnc	-1.13	-0.97150988	0.857861308	0	0
CPn1054CP0764	-1.13	-0.79850981	0.705333514	0	0
CPn0643CP0104rs19	-1.13	-0.81681045	0.721833929	0	0
CPn0688CP0058	-1.13	-0.99098862	0.875787617	0	0
CPj0820CPn0820	-1.13	-0.95091499	0.841707589	0	0
CPn0986CP0869yggH	-1.12	-0.91431102	0.814925059	0	0
CPn0098htrB	-1.12	-0.84108433	0.751612813	0	0
CPn0317CP0440infB	-1.11	-0.9106625	0.818405814	0.038192	0
CPn0483CP0271	-1.11	-0.8267037	0.744564994	0.038192	0
CPN0368_CP0389	-1.11	-0.86001415	0.774629078	0.038192	0
CPn0530CP0222spoU1	-1.11	-0.90879587	0.819017014	0.038192	0
CPn0590CP0158	-1.11	-0.85097512	0.768470743	0.038192	0
CPn0404CP0351	-1.1	-0.92396097	0.837988775	0.038192	0
CPn0034CP0742	-1.1	-0.87571359	0.795813594	0.038192	0
CPn0829CP1042	-1.1	-0.91321996	0.832020373	0.038192	0
CPn0611CP0136yacE	-1.1	-0.97306912	0.88808084	0.038192	0
CPn0612CP0135polA	-1.09	-0.81051702	0.743604244	0.038192	0
CPn0167CP0604	-1.09	-0.9317053	0.85517955	0.038192	0
CPn0733CP0013rs4	-1.08	-0.74476546	0.688431663	0.038192	0

CPn0364CP0393fer4	-1.08	-0.85491205	0.791470477	0.038192	0
CPn0601CP0146	-1.07	-0.84024982	0.78253242	0.038192	0
CPn0903CP0963ftsW	-1.07	-0.7713261	0.719167257	0.038192	0
CPn0630CP0117rl15	-1.07	-0.88233068	0.8244878	0.038192	0
CPn0366CP0391	-1.06	-0.87548834	0.823622533	0.038192	0
CPn0647CP0100rl3	-1.06	-0.87835391	0.832225218	0.038192	0
CPn0764CP1108	-1.05	-0.78895473	0.749416555	0.038192	0
CPn0753CP1119	-1.04	-0.86274934	0.826744093	0.038192	0.171
CPn0626CP0121rpoA	-1.04	-1.10404787	1.059233505	0.038192	0.198
CPn0435CP0318	-1.04	-0.71768189	0.691018698	0.038192	0.281
CPn0037CP0738ptsH	-1.04	-0.84754316	0.816623224	0.038192	0.297
CPn0290CP0468	-1.04	-0.90270078	0.870830928	0.038192	0.326
CPn0942CP0919dnaG	-1.03	-0.85938941	0.832589165	0.038192	0.428
CPn0484CP0270aroG	-1.03	-1.00620648	0.97801036	0.038192	0.508
CPn0321CP0436ychF	-1.03	-0.79844066	0.776964742	0.038192	0.536
CPn0041CP0733	-1.03	-0.78968205	0.769915072	0.038192	0.584
CPn0247CP0515rl13	-1.02	-0.74768393	0.733331741	0.038192	0.737
CPn0225CP0539	-1.02	-0.82363661	0.808130575	0.038192	0.747
CPn0914CP0952	-1.02	-0.80509143	0.790389294	0.038192	0.762
CPn0943CP0917	-1.02	-0.76159153	0.748055448	0.038192	0.775
CPn0292CP0466incC	-1.01	-0.71479849	0.70629944	0.038192	0.934
CPn0313CP0445acpS	-1.01	-0.91202671	0.904045369	0.038192	1.021
CPn0758CP1114folP	-1	-0.77678277	0.775449261	0.038192	1.219
CPn1015CP0838	-1	-0.88924243	0.889310106	0.038192	1.271
CPn0316CP0441nusA	-1	-0.9497764	0.950224163	0.038192	1.282
Cpn0019_pmp52	-0.99	-0.78172943	0.790648447	0.038192	1.605
CPn0989CP0866	-0.99	-0.85384435	0.866638018	0.038192	1.714
CPn0867CP1002rodA	-0.99	-0.76240153	0.773863219	0.038192	1.715
CPn0561CP0189euo	-0.98	-0.86001959	0.874276177	0.038192	1.762
CPn0819CP1052	-0.96	-0.7537287	0.784006649	0.069572	2.517
CPn0639CP0108rl29	-0.95	-0.8563841	0.899941298	0.069572	2.879
CPn0898CP0968groEL3	-0.94	-0.76419175	0.810796067	0.069572	3.231
CPn0463CP0289	-0.94	-0.73976246	0.787542109	0.069572	3.359
CPn0638CP0109rs17	-0.93	-0.77877437	0.840501988	0.069572	3.892
CPn0051	-0.93	-0.74990956	0.810078423	0.069572	3.928
CPn1003CP0850	-0.91	-0.75251723	0.823253689	0.069572	4.447
CPn0147CP0626	-0.9	-0.61785467	0.683392064	0.082623	4.914
CPn0358CP0400	-0.9	-0.68471947	0.757567695	0.082623	4.927
CPn0729CP0017	-0.9	-0.67028695	0.743801526	0.082623	5.056
CPn0830CP1041	-0.9	-0.68772462	0.764411629	0.082623	5.129
CPn0186CP0581	-0.9	-0.71322409	0.794626678	0.082623	5.233
CPn0114CP0659hemK	-0.9	-0.89180931	0.994240906	0.082623	5.262
CPn0319CP0438truB	-0.89	-0.77245196	0.863729705	0.082623	5.394
CPn0990CP0865infC	-0.89	-0.70699736	0.794952216	0.082623	5.646
CPn0196CP0571oppA2	-0.89	-0.77752257	0.875999422	0.082623	5.738
CPn1052CP0800	-0.89	-0.64707941	0.73024299	0.082623	5.814
CPn0532CP0220ribC	-0.89	-0.746285	0.842263784	0.082623	5.818
CPn0354CP0405	-0.89	-0.64540818	0.728794953	0.082623	5.842
CPn0122CP0651metG	-0.88	-0.78953377	0.899828824	0.129264	6.275
CPn0686CP0060	-0.87	-0.70398597	0.804618508	0.129264	6.411
CPn0071CP0704	-0.87	-0.74305327	0.856053416	0.129264	6.796
CPn0226CP0538	-0.87	-0.70414037	0.812680289	0.129264	6.884
CPn1068CP0782rnhB2	-0.85	-0.65330432	0.764262732	0.143931	7.56
CPn0619CP0128ndk	-0.85	-0.62133819	0.731389226	0.143931	7.879
CPn0640CP0107rl16	-0.84	-0.73646146	0.877532115	0.143931	8.519
CPn0109CP0665ileS	-0.83	-0.64898071	0.778967127	0.188867	8.911
CPn0738CP0007	-0.83	-0.66488754	0.798741223	0.188867	8.958
CPn0680CP0067ygo4	-0.83	-0.72468346	0.872113562	0.188867	9.054
CPn0656CP0091	-0.83	-0.68213899	0.822508634	0.188867	9.16
CPn0756CP1116rpoD	-0.83	-0.75105556	0.908089791	0.188867	9.31
CPn1043CP0809bioF2	-0.83	-0.69838733	0.845742798	0.188867	9.397
CPn0009CP0765	-0.83	-0.72980852	0.884194403	0.188867	9.422
CPn0352CP0407	-0.82	-0.74785582	0.9094182	0.188867	9.627

CPn0802CP1069trpS	-0.81	-0.65356843	0.808125569	0.217215	10.57
CPn0283	-0.81	-0.79293621	0.984117996	0.217215	10.79
CPn0736CP0009ygeD	-0.81	-0.72416734	0.899029872	0.217215	10.8
CPn0968CP0892	-0.8	-0.67970728	0.844560556	0.217215	10.85
CPn0344CP0416yaeL	-0.8	-0.64355122	0.800824077	0.217215	10.94
CPn0191CP0576glnQ	-0.8	-0.57931125	0.725184764	0.217215	11.29
CPn0315CP0442	-0.8	-0.61572406	0.771053227	0.217215	11.31
CPn0264CP0497ubiD	-0.8	-0.7033478	0.881102618	0.217215	11.33
CPn0814CP1057	-0.8	-0.65187696	0.81680962	0.217215	11.35
CPn0746CP1126	-0.78	-0.65221832	0.837693126	0.217215	12.84
CPn0900CP0966mraY	-0.78	-0.70849608	0.913631744	0.217215	13.08
CPn0575aprfB	-0.77	-0.63000267	0.813039014	0.217215	13.13
CPn0658	-0.77	-0.62156543	0.804349155	0.28824	13.3
CPn0934CP0927rnpA	-0.77	-0.61779248	0.799886395	0.28824	13.33
CPn0092CP0682atpK	-0.77	-0.65068703	0.843976162	0.28824	13.44
CPn1044CP0808bioB	-0.77	-0.52204374	0.681444442	0.28824	13.84
CPn0052CP0723hemC	-0.77	-0.63999134	0.836192301	0.28824	13.9
CPj05762CPn0576	-0.76	-0.63696147	0.835437099	0.28824	14.14
CPn0891CP0975mfd	-0.76	-0.65431388	0.865217367	0.28824	14.66
CPn0068CP0706	-0.75	-0.58726214	0.778755033	0.28824	14.85
CPn0279CP0479	-0.75	-0.5669078	0.756088433	0.28824	15.22
CPn0731CP0015	-0.75	-0.5929222	0.794611153	0.28824	15.53
CPn0224CP0541	-0.74	-0.63820377	0.857312547	0.28824	15.68
CPn0813CP1058pepP	-0.74	-0.55496229	0.751742551	0.28824	16.23
CPn0575CP0174yhhY	-0.74	-0.63734095	0.864332075	0.28824	16.31
CPn0163CP0608	-0.74	-0.5725008	0.778637255	0.358124	16.5
CPn0740tyrB	-0.73	-0.68801156	0.941303444	0.358124	16.89
CPn0235CP0527kdsB	-0.73	-0.65407041	0.902096599	0.358124	17.43
CPn1017CP0836lytB	-0.72	-0.5539151	0.765883042	0.358124	17.6
CPn0578CP0170yaeI	-0.72	-0.61340696	0.853477893	0.358124	18.03
CPn0475CP0279glgB	-0.72	-0.56531644	0.787261725	0.358124	18.09
CPn0751CP1121	-0.71	-0.53392977	0.74979346	0.358124	18.66
CPn0487CP0267	-0.71	-0.63380585	0.890275995	0.358124	18.67
CPn1065	-0.71	-0.63126047	0.894048809	0.426901	19.24
CPn0603CP0144hemZ	-0.7	-0.62890973	0.902779755	0.426901	20.17
CPn0589CP0159	-0.69	-0.65123019	0.939106859	0.426901	20.49
CPn0979CP0877htrA	-0.69	-0.53806675	0.778453391	0.426901	20.72
CPn0242CP0520	-0.69	-0.50115274	0.729197232	0.463567	21.12
CPn1042CP0810bioD	-0.68	-0.54661156	0.799217185	0.463567	21.46
CPn0428CP0325nqr3	-0.68	-0.62367782	0.915885453	0.463567	21.77

Estimated Miss rates for Delta=0.494663712458557

Quantiles	Cutpoints	Miss Rate(%)
0 -> 0.05	-0.666 -> -0.603	77.7
0.05 -> 0.1	-0.603 -> -0.534	69.17
0.1 -> 0.15	-0.534 -> -0.48	68.06
0.15 -> 0.2	-0.48 -> -0.394	23.18
0.2 -> 0.25	-0.394 -> -0.349	50.42
0.25 -> 0.75	-0.349 -> 0.44	2.57
0.75 -> 0.8	0.44 -> 0.52	41.04
0.8 -> 0.85	0.52 -> 0.589	66.55
0.85 -> 0.9	0.589 -> 0.635	84.45
0.9 -> 0.95	0.635 -> 0.699	78.15
0.95 -> 1	0.699 -> 0.758	82.82
