# Supplementary material for: Gene Expression Profiles of Chlamydophila pneumoniae during the Developmental Cycle and Iron Depletion–Mediated Persistence
Source: PLoS Pathog. 2007 Jun 22;3(6):e83. doi: 10.1371/journal.ppat.0030083 (PMC1894823; doi:10.1371/journal.ppat.0030083)
Supplement: Supplementary file 14 [file ppat.0030083.st003.pdf]

Mäurer et. al 2007

Table S3 –
Genes Clustered in Figure 1B and Figure 4B

Maeurer *et al.*
Expression Profiles for all significant genes from the
(A) acute
(B) persistent infection
of *Cpn*

(A) acute infection

NAME	Cluster	Persistency	in EB	6h	12h	18h	24h	36h	48h	60h	72h
CPn0369CP0388	12	DOWN		0.88	-0.66	-1.2	-0.51	-1.01	0	1.01	1.57
CPn0607CP0140glgC	12	DOWN		-0.06	0.24	-0.42	0.08	-0.41	0	-0.12	0.41
CPn0172guaB	12			0.25	-0.37	-0.24	0.24	-0.21	0	0.31	0.77
CPn0494CP0260	12	DOWN		0.12	-0.4	-0.95	-0.63	-0.54	0	0.96	1.73
CPn0178CP0590	12			0.61	-0.4	-0.43	-0.87	-0.35	0	0.42	0.66
CPn0537CP0215	12	DOWN		0.06	0.57	-0.64	-0.77	-1.47	0	1.42	1.94
CPn0493CP0261	12			0.84	0.24	0.2	0.46	-0.5	0	0.78	1.36
CPn0254CP0507	12			0.31	-0.14	0.11	0.05	-0.12	0	0.45	0.52
CPn1025CP0827pgi	12	DOWN		-0.18	-0.26	-0.39	-0.07	-0.21	0	0.4	1.09
CPn0065CP0709	12	DOWN		0.2	-0.04	-1.07	-0.73	-1.31	0	0.51	1.67
CPn1006CP0847	12	DOWN		1.42	-0.38	-0.98	-0.97	-1.87	0	0.98	1.75
CPn0333CP0425	12			0.59	-1.02	-2.16	-2.24	-3.39	0	2.17	2.12
CPn0728CP0018	12	DOWN	Yes	-0.63	-0.89	-0.99	-0.83	-1.37	0	0.84	1.88
CPn0162CP0609	12			0.35	-0.05	-0.28	-0.43	-0.08	0	0.16	0.69
CPn0143CP0630yxjG1	12	UP	Yes	0.14	0.18	-0.15	0.02	-0.17	0	0.1	0.64
CPn0142	12	DOWN		0.46	-0.55	-0.56	-0.56	-0.18	0	0.42	0.55
CPn0559CP0191	12			1.39	0.21	0.16	0.29	-0.59	0	0.99	2.23
CPn0040CP0735dnaX1	12			0.16	-0.93	-0.43	-0.1	0.23	0	0.48	0.36
CPn0332	12			1.73	0.31	0.79	-0.33	-1.59	0	2.29	3.37
CPn0930CP0935	12	DOWN		0.15	-0.12	-0.4	-0.2	-0.45	0	0.06	1.05
CPn0330CP0427	12	DOWN		-0.16	-0.33	-1.34	-0.57	-1.18	0	0.58	1.89
CPn0586CP0162atoC	12			0.38	-0.62	-0.02	-0.22	0.04	0	0.81	0.48
CPn0340CP0417	12	DOWN		0.23	-0.68	-0.06	-0.11	-0.7	0	0.68	1.08
CPn1023CP0829	12	DOWN		0.19	-0.37	-0.77	-0.3	-1.29	0	0.74	1.51
CPn0524CP0229	12	DOWN		0.68	-0.92	-0.99	-0.52	-1.54	0	0.74	1.5
CPn0189CP0578	12	DOWN		1.04	-0.74	-1.24	-0.41	-0.65	0	0.38	0.57
CPn0667CP0080	12			0.27	-0.91	-0.68	-0.48	-1	0	0.93	1.01
CPn0726	12			0.51	0.12	-0.47	-0.52	0.71	0	0.17	1.42
CPn0129CP0642	12			0.32	0.15	-0.56	-0.41	-0.13	0	0.29	0.44
CPn0190CP0577	12	UP		0.66	-0.25	0.6	0.28	-0.57	0	0.82	1.2
CPn0720CP0026	12	DOWN	Yes	-0.26	-0.18	-0.99	-0.48	-0.43	0	0.33	1.43
CPn0209CP0557	12			0.39	-0.35	-0.88	-0.35	-0.15	0	0.88	0.4
CPn0671CP0076	12			-0.2	-0.56	-0.69	-0.42	0.2	0	0.57	0.96
CPn0717CP0029	12			-0.15	-0.61	-0.29	-0.86	-0.27	0	0.55	0.78
CPn0724CP0022	12	DOWN		0.01	-0.2	-0.58	-0.29	-0.88	0	0.23	0.75
CPn0676CP0071	12	DOWN		-0.05	-0.94	-1.43	-1.26	-2.21	0	1.27	2.36
CPn0365CP0392	12			0.12	-0.39	-0.01	-0.67	-0.5	0	0.51	1.11
CPn0232CP0532	12			0.01	0.1	-0.74	-0.49	-0.14	0	0.73	0.34
CPn0234CP0529	12	DOWN		0.51	-0.06	0.28	0.17	-0.8	0	0.52	1.6
CPn0473CP0281	12	UP		0.49	-0.27	0.83	0.71	-0.27	0	0.52	1.7
CPn0184CP0584efp1	12		Yes	-0.23	-0.01	-0.07	0.04	0.02	0	0.18	0.33
CPn0678	12			0.84	-0.58	-0.15	-0.16	-1.57	0	1.31	2.56
CPn0974CP0885sucD	11		Yes	-0.29	-0.57	-0.66	-1.17	-1.19	0	0.1	0.06
CPn0096CP0678uvrA	11			-0.08	-0.18	-0.23	-0.45	-0.03	0	0.03	0.24
CPn0852CP1017	11	DOWN		-2.43	-2.23	-2.58	-2.25	-2.72	0	0.4	1.14
CPn0362CP0395rpsD	11	DOWN		-1.02	-1.38	-1.31	-1.54	-2.06	0	0.24	1.57
CPn0973CP0886sucC	11		Yes	-0.67	-1.76	-1.8	-1.06	-1.27	0	0.23	-0.04

CPn0809CP1062	11	DOWN		-0.73	-1.99	-3	-1.85	-2.32	0	0.88	2.25
CPn0377CP0379sucB1	11			-0.67	-1.45	-1.24	-0.75	-1.03	0	0.1	0.21
CPn0791CP1081	11	DOWN		-0.07	-1.26	-1.16	-0.98	-0.9	0	0.31	0.22
CPn0794CP1077	11	DOWN		-1.27	-1	-2.77	-2.18	-1.27	0	0.25	0.36
CPn0978CP0878	11			-0.38	-0.39	-1.03	-0.35	-0.68	0	-0.14	0.09
CPn0810CP1061	11	DOWN		-2.1	-3.09	-3.55	-2.6	-3.53	0	0.89	2.28
CPn0808CP1063	11	DOWN	Yes	-0.94	-1.73	-2.61	-1.75	-2.53	0	0.53	2.15
Cpn0016_pmp41	11	DOWN		-0.97	-0.81	-3.61	-3.02	-1.13	0	-0.13	0.28
Cpn0017_pmp42	11	DOWN		-0.08	-0.78	-0.5	-0.33	-0.53	0	0.11	0.2
CPn0811CP1060lcrH1	11	DOWN		-2.65	-2.97	-4.28	-2.41	-3.72	0	1.22	2.25
CPn0466CP0286pmp15	11			-0.01	-0.92	-0.62	-0.91	-0.33	0	-0.16	0.18
CPn0538CP0214	11	DOWN		-0.68	-1.07	-1.79	-0.64	-1.79	0	0.85	2.08
CPn0853CP1016	11	DOWN		-0.66	-1.43	-1.46	-2.03	-1.58	0	0.53	0.82
CPn0587CP0161yvyD	11			-0.2	-1.26	-1.22	-1.22	-0.86	0	0.42	1.02
CPn0324CP0433lcrE	11	DOWN	Yes	-0.49	-1.27	-1.53	-1.56	-1.27	0	-0.04	0.25
CPn0727CP0019	11	DOWN		-0.12	-0.84	-1.77	-1.54	-3.07	0	0.56	1.32
CPn0622CP0125	11			-0.37	-0.91	-1.34	-1.53	-1.2	0	-0.35	-0.25
CPn0256CP0505	11			-0.18	-0.21	-0.3	-0.44	-0.6	0	-0.29	0.02
CPn0384CP0371hctB	11	DOWN		-2.39	-3.86	-4.24	-3.26	-4.11	0	0.66	1.05
CPn0127CP0645ytfF	11			0.07	-0.37	-0.98	-0.49	-0.31	0	0.37	0.53
CPn0188CP0579	11	DOWN		-0.22	-0.71	-1.13	-0.9	-1.31	0	0.12	0.64
CPn0725	11	DOWN		-0.42	-1.31	-1.45	-1.17	-1.76	0	0.79	1.2
CPn0445CP0308pmp7	11	DOWN	Yes	-0.35	-1.87	-1.97	-1.25	-1.24	0	0.21	0.26
CPn0204CP0563	11	DOWN		-0.3	-0.7	-1.38	-0.6	-0.3	0	0.66	0.71
CPn0677CP0070	11	DOWN	Yes	-0.81	-1.76	-2.35	-1.59	-2.71	0	1.08	2.38
CPn0693	11	DOWN		-0.06	-0.8	-1.4	-0.84	-1.56	0	-0.01	0.79
CPn0700CP0046	11	DOWN		-0.32	-1.4	-2.08	-1.63	-1.19	0	0.25	0.38
CPn0179CP0589	11			-0.38	-0.7	-1.28	-1.3	-0.36	0	0.68	0.66
CPn0742CP0003	11	DOWN	Yes	-0.08	-1.68	-2.03	-2.08	-1.96	0	0.93	2.01
CPn0419CP0335pbp3	11			0.04	-0.52	-0.74	-0.39	-0.57	0	0.86	0.04
CPn0994CP0861	11	DOWN		-0.07	-0.4	-0.99	-0.33	-1.04	0	0.06	1.02
CPn0557CP0195omcB	11	DOWN	Yes	-2	-2.98	-3.42	-2.96	-2.74	0	0.52	1.59
CPn0558CP0193	11	DOWN		-0.98	-2.91	-3	-3.4	-2.16	0	1.09	0.72
CPn0307CP0451glgP	11	DOWN	Yes	-0.1	-0.89	-1.72	-0.38	-1.36	0	0.52	0.4
CPn0383CP0372	11			-0.06	-0.28	-0.12	-0.07	-0.31	0	-0.05	-0.01
CPn0998CP0857ftsH	11		Yes	-0.41	-0.58	-0.9	-0.5	-0.33	0	-0.09	0.46
CPn0164CP0607	11			-0.11	-0.41	-0.65	-0.85	-0.63	0	-0.01	0.73
CPn1005CP0848	11	DOWN		0.14	-1.37	-2.19	-1.81	-2.39	0	0.72	1.89
CPn0584CP0164atoS	11	DOWN		-0.09	-1	-1.59	-0.91	-1.01	0	0.11	0.1
CPn0472CP0282	11	DOWN		-0.46	-0.99	-1.32	-0.7	-1.14	0	0.51	1.78
CPn0085CP0690	11			-0.13	-0.13	-0.44	-0.66	-0.23	0	-0.01	0.24
CPn1004CP0849	11	DOWN		0.23	-1.28	-2.47	-0.62	-2.85	0	0.52	1.37
CPn0447CP0306pmp9	11			-0.21	-0.83	-0.86	-0.66	-0.48	0	0.2	0.04
CPn0194CP0573gcp2	10			-1.98	-1.84	-1.66	-1.4	-0.77	0	0.58	0.71
CPn0467CP0285pmp16	10	DOWN		-0.76	-1.82	-1.63	-1.13	-0.34	0	0.27	0.18
CPn0860CP1009fliF	10	DOWN		-2.46	-2.59	-3.21	-2.37	-1.02	0	0.64	0.4
CPn0444CP0309pmp6	10	DOWN	Yes	-1.26	-1.73	-1.64	-1.52	-0.97	0	-0.18	0.32
CPn0446CP0307pmp8	10	DOWN	Yes	-1.32	-2.98	-2.25	-1.74	-0.84	0	-0.04	0.13
CPn0854CP1015porB	10			-1.49	-1.79	-2.17	-1.18	-0.84	0	-0.04	0.43
CPn0857CP1012	10	DOWN		-0.5	-0.87	-1.65	-1.76	-0.4	0	-0.17	-0.52
CPn0125CP0648	10			-1.49	-1.23	-1.16	-1.26	-0.43	0	0.12	0.06
CPn0451CP0302pmp11	10		Yes	-0.5	-1.08	-1.83	-1.13	-0.34	0	0.42	0.21
CPn0453CP0299pmp13	10	DOWN	Yes	-2.53	-2.52	-2.26	-2.12	-0.6	0	0.16	0.38
CPn0497CP0257	10			-0.84	-1.53	-0.79	-0.63	-0.17	0	0.31	0.37
CPn0786CP1086dsbD	10			-0.59	-1	-0.96	-0.69	-0.5	0	0.03	0.31
CPn0498CP0256	10			-1.98	-2.41	-1.94	-1.26	-0.27	0	0.52	0.56

CPn0062CP0713	10			-2.11	-2.05	-1.85	-1.58	-0.37	0	0.09	0.06
CPn0769CP1103topA	10	DOWN		-0.52	-0.83	-0.6	-0.12	-0.07	0	0.08	0
CPn0056CP0719mrsA	10			-0.26	-0.76	-1.13	-0.82	-0.46	0	-0.24	-0.26
CPn0750CP1122tctD	10	DOWN	Yes	-2.31	-2.18	-2.13	-1.99	-1.27	0	0.21	-0.01
CPn0731CP0015	10			0.01	-0.01	-0.07	-0.35	-0.04	0	0.22	-0.24
CPn0777CP1095groEL2	10	DOWN		-0.59	-1.11	-0.61	-0.63	-0.62	0	0.22	-0.12
CPn0195CP0572oppA1	10	DOWN		-1.87	-2.07	-1.67	-1.5	-0.81	0	0.45	0.56
CPn0669CP0078	10	DOWN		-2.58	-1.99	-2.05	-2.08	-0.8	0	0.32	0.38
CPn0703CP0043	10			-1.67	-1.44	-1.63	-1.74	-1.05	0	0.39	0.08
CPn0774CP1098	10			-1.35	-1.61	-1.2	-1.24	-0.35	0	0.32	0.09
CPn0437CP0316clpC	10		Yes	-0.38	-0.5	-0.82	-0.91	-0.1	0	-0.18	-0.08
CPn0556CP0196crpA	10			-1.97	-1.69	-3.07	-2.05	-1.31	0	-0.41	0.38
CPn0106CP0668phoH	10		Yes	-1.16	-0.92	-1.03	-1.02	-0.12	0	0.47	0.08
CPn0103CP0671cydB	10			-1.17	-0.76	-1.54	-0.87	-0.35	0	-0.13	-0.34
CPn0105CP0669	10			-1.27	-1.54	-1.71	-1.32	-0.33	0	0.57	0.67
CPn0824CP1047yscS	10			-0.59	-0.82	-0.66	-0.59	-0.55	0	-0.33	-0.27
CPn0822CP1049	10			-1.56	-2.1	-2.8	-2.03	-0.64	0	0.36	0.52
CPn0534CP0218dksA	10		Yes	-1.96	-1.69	-1.57	-1.04	-0.71	0	0.12	-0.12
CPn0540CP0212pmp20	10		Yes	-1.7	-1.6	-1.6	-1.37	-0.47	0	-0.13	-0.11
CPn0797CP1074	10		Yes	-0.77	-1.8	-2.34	-1.29	0.08	0	-0.16	-0.36
CPn0796CP1075	10			-1.48	-3.04	-3.52	-1.74	0.77	0	-0.45	0.35
CPn0793CP1079rbsU	10			-0.73	-1.76	-1.26	-0.86	-0.18	0	-0.15	-0.63
CPn0877CP0992ybcL	10			-1.31	-1.46	-0.98	-1.01	0.01	0	0.24	0.14
CPn0701CP0045karG	10	DOWN		-0.15	-1.45	-1.87	-2.01	-0.56	0	-0.28	-0.28
CPn0971CP0888yccA2	10			-0.98	-1.4	-1.59	-1.15	-0.31	0	0.49	0.45
CPn1013CP0840fumC	10		Yes	-1.24	-1.37	-1.89	-1.13	-0.64	0	0	-0.08
CPn0933CP0928	10		Yes	-2.03	-2.55	-3.13	-2.22	-1.04	0	0.32	0.38
CPn0926CP0940	10		Yes	-1.3	-1.49	-1.67	-1.17	-0.27	0	0.23	0.18
CPn0177CP0592	10			-1.19	-2.14	-1.77	-0.77	0.09	0	0.24	0.43
CPn1016CP0837	10			-1.78	-2.26	-2.8	-1.9	-0.66	0	-0.41	0.16
CPn0355CP0404	10			-1.77	-2.08	-2.14	-1.75	-0.14	0	0.05	-0.19
CPn0133CP0639	10			-0.02	-0.24	-0.47	0	0.15	0	0.12	-0.09
CPn0399CP0356	10			-0.59	-0.8	-1.03	-0.52	-0.43	0	0.12	-0.01
CPn0001CP0775	10			-1.75	-2.93	-2.39	-2.22	0.51	0	0.33	1.14
CPn1002CP0851	10			-0.34	-1.02	-0.43	-0.52	0.02	0	0.51	0.49
CPn0160CP0611	10			-0.43	-0.79	-0.7	-0.28	-0.33	0	-0.57	-0.36
CPn0161CP0610	10			-1.1	-1.19	-1.03	-0.72	-0.46	0	-0.16	0.09
CPn1032CP0820	10	DOWN	Yes	-1.87	-2.71	-2.96	-1.71	-1.24	0	0	0.02
Cpn0015_pmp32	10			-1.36	-1.17	-1.44	-1.24	-0.39	0	-0.13	0.01
CPn09691yccA1	10	UP		-0.79	-1.86	-1.47	-0.59	0.05	0	0.19	0.7
CPn0325CP0432sycE	10	DOWN		-2.24	-1.8	-2.39	-2.21	-1.07	0	-0.29	-0.18
Cpn0014_pmp31	10	DOWN		-2.77	-2.8	-2.76	-2.09	-0.38	0	0.24	0.29
CPn0323CP0434lcrD	10			-0.66	-0.72	-1.13	-0.97	-0.52	0	0.08	0.18
Cpn0018_pmp51	10	DOWN		-1.4	-1.54	-2.55	-1.55	-1.15	0	0.47	0.4
CPn0956CP0904	10	DOWN		-1.73	-1.79	-1.46	-2.05	-1.01	0	-0.23	0.16
CPn0349CP0411ytgA	10			0.02	-0.39	-0.88	-0.24	0.01	0	0.14	-0.15
CPn0350CP0409	10			-0.48	-0.22	-0.65	-0.76	0.13	0	0.16	0.29
CPn1028CP0824mdhC	10		Yes	-1.33	-1.63	-1.42	-0.98	0.06	0	-0.11	-0.16
CPn0398CP0357	10			-1.95	-2.28	-2.63	-1.95	-0.5	0	0.37	0.41
CPn1033CP0819	10	DOWN		-2.22	-3	-3.51	-3.58	-1.1	0	0.14	0.39
CPn0140CP0632yqdE	10	DOWN		-2.08	-1.24	-1.55	-1.76	-0.48	0	0.32	0.31
CPn0216CP0549	10			-0.58	-1.27	-1.8	-0.98	-0.56	0	0.02	0.08
CPn0227CP0537dsbB	10	DOWN		-1.54	-1.29	-2.49	-1.61	-1.05	0	-0.27	-0.06
CPn0139CP0633yqgE	10	DOWN		-1.83	-1.4	-1.7	-1.15	-0.57	0	0.02	0.1
CPn0894CP0972amn	10	DOWN	Yes	-1.34	-1.52	-1.52	-1.12	-0.64	0	0.21	-0.27
CPn0200CP0567oppC1	10			-0.17	-0.72	-0.44	-0.27	-0.1	0	0.09	0.12

CPn0207CP0560ybhI	10			-0.13	-1.16	-0.8	-0.24	-0.28	0	-0.1	-0.12
CPn0421CP0333yabC2	10			-0.78	-0.51	-0.64	-0.66	-0.3	0	0.21	0.14
CPn1069CP0781yfgA	10			-0.55	-1.17	-0.84	-0.73	0.2	0	0.3	0.27
CPn0886CP0980	10			-2.39	-4.06	-4.94	-3.15	-1.29	0	0.84	1
CPn0433CP0320gcsH	10			-0.65	-0.39	-0.75	-0.37	-0.28	0	-0.1	-0.11
CPn0228CP0536dsbG	10	DOWN		-2.12	-2.21	-2.9	-3.11	-1.18	0	0.03	-0.17
CPn0970CP0889tyrP2	10			-1	-1.9	-1.14	-0.67	-0.08	0	-0.21	0.1
CPn0229CP0535	10			-0.6	-0.94	-1.09	-0.23	-0.85	0	-0.16	-0.22
CPn0416CP0338himD	10	DOWN		-1.9	-3.33	-3.13	-2.45	-1.14	0	0.03	0.45
CPn0138CP0634hemL	10			-0.1	-0.26	-0.43	-0.32	0.23	0	0.04	0.15
CPn1034CP0818	10			-0.15	-0.49	-1.08	-0.27	0.25	0	0.55	0.1
CPn0735CP0011	9			-2.42	-1.61	-1.18	-0.82	0.62	0	-0.24	-0.6
CPn0687CP0059	9			-0.41	-0.43	-0.25	-0.16	0.37	0	0.18	-0.14
CPn0782CP1090tolB	9			-1.16	-0.97	-0.2	-0.26	0.22	0	0.03	0.06
CPn0785CP1087exbB	9			-1.32	-1.19	-0.35	-0.03	0.12	0	-0.21	-0.27
CPn1070CP0780	9			-0.09	-0.57	-0.1	0.34	0.07	0	0.16	-0.04
CPn0553CP0199	9			-1.57	-1.42	-0.52	-0.18	0.24	0	0.17	0.02
CPn0624CP0123gapA	9		Yes	-1.14	-1.48	-0.68	-0.76	0.23	0	-0.28	0.15
CPn0789CP1083sdhA	9			-1.47	-1.46	-0.78	-0.26	0.17	0	-0.48	-0.35
CPn0695CP0051ompA	9		Yes	-1.6	-1.85	-0.99	-0.76	0.22	0	-0.22	0.96
Cpn0061_CP0714	9		Yes	-0.62	-0.64	-0.27	-0.2	0.54	0	-0.28	-0.29
CPn0206CP0561	9			-0.76	-0.77	-0.61	-0.7	0.28	0	0.07	0.2
CPn0193CP0574argR	9			-0.57	-0.53	-0.48	-0.09	0.04	0	0.22	0.22
CPn0652CP0095lpxC	9		Yes	-0.98	-0.47	-0.25	-0.36	0.11	0	0.4	-0.16
CPn0604CP0143fliY	9			-1.17	-1.5	-0.95	-0.85	0.1	0	0.16	-0.19
CPn0754CP1118rs20	9			-0.31	-0.7	0.05	0.18	0.35	0	-0.12	0.01
CPn0594CP0154pheT	9			-1.92	-0.79	-0.01	-0.8	-0.02	0	0.52	0.41
CPn0608CP0139	9		Yes	-1.1	-0.68	-0.89	-0.53	0.42	0	0.19	0.2
CPn0262CP0499surE	9			-0.87	-0.77	-0.63	-0.09	-0.04	0	0.25	0.15
CPn0231CP0533tauB	9	UP		-0.27	-0.11	-0.22	-0.33	0.6	0	-0.09	0.18
CPn0581CP0167	9			-1.32	-1.61	-0.88	-0.48	0.37	0	0.13	-0.09
CPn0577CP0171	9			-1.71	-1.44	-0.45	-0.31	0.35	0	0.08	-0.15
CPn0286CP0472mgtE	9			-0.51	-0.2	-0.2	0.01	0.18	0	0.13	0.3
CPn1001CP0852yfhC	9	UP	Yes	-0.34	-0.42	-0.1	0.09	0.31	0	0.3	0.25
CPn0132CP0640	9			-1.4	-1.14	-0.44	-0.6	0.14	0	-0.07	-0.07
CPn0876CP0993dagA2	9			-1.41	-1.68	-1.1	-1.03	0.04	0	0.78	0.7
CPn0902CP0964nlpD	9	UP		-1.32	-1.28	0.08	0.27	0.18	0	0.2	0.15
CPn0144CP0629clpB	9		Yes	-1.79	-1.71	-0.33	-0.59	-0.08	0	0.1	0.39
CPn0901CP0965murD	9			-2.39	-1.68	-0.7	-0.42	0.09	0	0.3	0.05
CPn0936CP0925rl36	9			-0.43	-0.15	-0.47	-0.38	0.38	0	0.14	0.06
CPn0110CP0664lepB	9			-0.84	-0.35	-0.25	-0.29	0.23	0	0.05	0.02
CPn0111CP0662	9			-0.65	-0.34	-0.28	-0.26	0.25	0	0.21	-0.15
CPn0495CP0259aspC	9		Yes	-0.55	-0.61	0.05	-0.2	0.17	0	0.33	0.4
CPn0048CP0726yqfF	9			-0.86	-0.84	-0.15	-0.23	0.51	0	0.02	-0.29
CPn0963CP0897pmp21	9		Yes	-2.41	-3.27	-0.99	-0.43	0.23	0	0.04	-0.4
CPn0913CP0953	9			-1.23	-1.02	-0.16	-0.15	0.53	0	-0.14	-0.21
CPn0833CP1037	9		Yes	-0.57	-0.92	-0.59	-0.4	0.27	0	0.09	-0.15
CPn0479CP0275phnP	9		Yes	-1.46	-0.96	-0.43	0.05	0.04	0	-0.12	-0.06
CPn0112CP0661rl31	9	UP		-0.68	-1.13	-0.82	-0.7	0.52	0	-0.18	-0.13
CPn0047	9	UP		-1.06	-0.78	-0.32	0	-0.02	0	0.1	-0.33
CPn0427CP0326nqr2	9			-0.42	-0.63	-0.25	-0.31	0.21	0	0.24	-0.22
CPn0865CP1004	9			-0.71	-0.42	-0.36	-0.64	0.3	0	0.17	-0.11
CPn0337CP0421smpB	9	UP		-0.33	-0.1	-0.21	-0.25	0.19	0	0.49	0.23
CPn0872CP0997ribA/ribB	9			-1.88	-1.22	-0.39	-0.27	0.21	0	0	0.04
CPn0798CP1073	9	UP		-1.48	-1.41	-0.87	-0.93	-0.06	0	-0.06	0.09
CPn0800CP1071eno	9		Yes	-0.82	-1.11	0	-0.21	0.18	0	-0.11	-0.4

CPn0441CP0312	9			-1.51	-1.39	-0.33	-0.16	0.37	0	-0.23	-0.7
CPn0873CP0996ribE	9		Yes	-0.74	-1.06	-0.55	-0.24	0.56	0	0.09	-0.14
CPn0959CP0901cafE	9		Yes	-0.61	-0.67	-0.4	-0.38	0.07	0	0.2	0.22
CPn0490CP0264	9			-1.86	-1.51	-0.52	-0.54	-0.01	0	0.04	-0.43
CPn0135CP0637groES	9		Yes	-0.77	-0.67	-0.21	0.46	0.69	0	0.1	0.84
CPn0422CP0331	8		Yes	-1.45	-0.94	-0.82	-0.58	-0.35	0	0.27	0.11
CPn0248CP0513ycfV	8			-0.75	-0.43	-0.41	-0.54	-0.1	0	-0.11	-0.11
CPn0434CP0319	8			-0.6	-0.44	-0.24	-0.36	-0.13	0	-0.09	0.26
CPn0136CP0636pepF	8			-0.92	-0.3	-0.64	-0.33	0.18	0	-0.1	-0.07
CPn0250CP0511rl33	8			-0.98	-0.41	-0.41	-0.18	-0.2	0	-0.24	-0.16
CPn0423CP0330	8			-1.94	-1.01	-0.89	-1.07	-0.16	0	-0.02	-0.19
CPn0067CP0707	8			-1.17	-0.64	-1.08	-0.45	-0.15	0	-0.49	-0.34
CPn0659CP0088	8		Yes	-0.71	-0.47	-0.56	-0.44	-0.19	0	-0.07	-0.31
CPn0413CP0341msbA	8			-0.88	-0.61	-0.75	-0.26	0.14	0	-0.53	-0.38
CPn0415CP0339	8	DOWN		-1.51	-1.18	-1.08	-0.51	-0.37	0	-0.51	-0.24
CPn0661mip	8			-2.22	-2.04	-1.51	-1.01	-0.23	0	-0.2	0
CPn0134CP0638groEL1	8		Yes	-1.31	-0.89	-0.71	-1.19	-0.31	0	-0.41	0.11
CPn0670rsbW	8	DOWN	Yes	-2.17	-1.43	-1.23	-1.24	-0.53	0	0.05	0.23
CPn0684CP0062parB	8		Yes	-1.7	-0.95	-0.74	-0.48	-0.11	0	-0.05	-0.17
CPn0083CP0692tal	8			-1.13	-0.75	-0.85	-0.59	-0.5	0	-0.29	-0.15
CPn0488CP0266ycfF	8		Yes	-1.55	-0.98	-0.42	-0.72	-0.58	0	-0.74	-0.3
CPn0489CP0265	8		Yes	-1.26	-0.86	-1	-0.44	-0.13	0	-0.11	-0.14
CPn0314CP0444trxB	8		Yes	-1.41	-0.7	-0.65	-0.42	-0.35	0	-0.18	-0.27
CPn0564CP0186secD/secF	8			-1.16	-0.69	-0.73	-0.22	-0.42	0	-0.55	-0.19
CPn0301CP0457	8		Yes	-2.51	-1	-0.7	-0.66	0.17	0	-0.04	-0.03
CPn0100CP0674	8			-1.6	-0.93	-0.35	-0.24	0.06	0	-0.22	0.04
CPn0322CP0435yscU	8			-1.63	-0.81	-1	-1.05	-0.35	0	-0.23	0.08
CPn0533CP0219	8			-1.61	-0.46	-0.82	-0.5	0.06	0	-0.39	-0.32
CPn0571CP0178murA	8			-1.23	-0.62	-0.44	-0.35	-0.11	0	-0.53	-0.48
CPn0535CP0217lspA	8			-1.77	-1.42	-1.02	-0.75	-0.18	0	-0.36	-0.28
CPn0153CP0618leuS	8			-0.91	-0.25	-0.45	-0.37	0.06	0	-0.52	-0.28
CPn0546CP0206rl21	8			-0.93	-0.27	-0.29	-0.25	-0.17	0	-0.03	-0.01
CPn0295CP0463	8			-1.77	-0.56	-0.78	-0.31	-0.16	0	-0.13	-0.12
CPn0525CP0228	8		Yes	-0.32	0.23	-0.43	-0.21	0.12	0	-0.32	-0.15
CPn0260CP0501secA1	8	UP		-1.48	-0.29	-0.83	-0.48	0.14	0	-0.47	-0.26
CPn0389CP0366	8			-2.19	-0.43	-0.81	-0.88	-0.13	0	-0.34	-0.31
CPn0621CP0126ruvC	8			-1.4	-1.44	-0.8	-0.63	-0.08	0	-0.19	-0.32
CPn0616CP0131dnaB	8		Yes	-1.26	-0.59	-0.2	-0.94	-0.08	0	0.07	0.06
CPn0390CP0365ruvB	8			-1.72	-0.5	-0.68	-0.74	-0.22	0	0.4	0.6
CPn0171CP0599guaA	8			-0.9	-0.67	-0.86	-0.48	-0.32	0	-0.37	0.23
CPn0387CP0368	8			-1.06	-0.27	-0.75	-0.3	-0.49	0	0	-0.38
CPn0282CP0476xasA	8			-1.6	-0.64	-0.78	-0.75	-0.62	0	-0.87	-0.88
CPn0595CP0153	8			-1.4	-0.55	-0.24	-0.46	-0.27	0	-0.03	-0.15
CPn0104CP0670	8		Yes	-1.73	-0.72	-1.25	-0.61	0.07	0	0.19	0.07
CPn0148CP0625	8			-0.71	-0.59	-0.53	-0.28	0	0	-0.22	-0.02
CPn0154CP0617gseA	8			-1.36	-0.36	-0.82	-0.68	-0.16	0	-0.73	-0.32
CPn0547CP0205ygbB	8			-1.14	-1.34	-1.16	-0.48	0.06	0	-0.31	-0.4
CPn0702CP0044yscC	8	DOWN	Yes	-2.36	-0.98	-1.43	-1.51	-0.73	0	-0.56	-0.23
CPn0828CP1043yscJ	8			-2.02	-1	-1.01	-1.17	-1.09	0	-0.03	0.16
CPn0827CP1044	8			-2.17	-0.76	-0.86	-1.07	-0.37	0	0.08	-0.2
CPn0840CP1029	8			-2.12	-1.82	-1.13	-0.94	-0.26	0	-0.12	-0.4
CPn0834CP1035	8			-1.62	-1.11	-0.96	-0.74	-0.3	0	0.06	-0.08
CPn0825CP1046yscR	8			-1.48	-0.8	-0.81	-0.56	-0.21	0	-0.26	-0.05
CPn0846CP1023clpX	8			-1.92	-0.79	-0.47	-0.47	-0.3	0	-0.54	-0.45
CPn0826CP1045yscL	8	DOWN	Yes	-1.44	-0.92	-1.1	-1.01	-0.29	0	-0.27	-0.15
CPn0044CP0729	8			-0.85	-0.59	-0.38	-0.05	-0.51	0	0.28	-0.09

CPn0849CP1020	8	DOWN		-1.52	-1.44	-1.2	-0.89	-0.11	0	-0.17	-0.56
CPn0770CP1102	8			-1.02	-0.83	-0.6	-0.32	-0.25	0	0.02	-0.32
CPn0057CP0718sodM	8		Yes	-1.52	-0.44	-0.61	-0.5	0.26	0	-0.47	-0.4
CPn0781CP1091pal	8			-1.71	-1.29	-0.83	-0.59	-0.41	0	-0.01	0.12
CPn0778CP1094ahpC	8	UP	Yes	-1.41	-0.67	-0.36	-0.83	0.28	0	-0.62	-0.57
CPn0806CP1065thrS	8	UP	Yes	-1.29	-1.26	-0.47	-0.32	-0.45	0	0.1	-0.3
CPn0803CP1068	8		Yes	-2.65	-1.34	-0.74	-0.5	0.03	0	-0.32	0
CPn0704fliN	8			-1.8	-0.79	-0.85	-0.89	-0.73	0	-0.13	-0.2
CPn0850CP1019mreB	8			-1.59	-0.91	-0.57	-0.34	-0.21	0	-0.53	-0.39
CPn0761CP1111	8			-2.49	-1.16	-0.65	-0.56	0.03	0	-0.64	-0.38
CPn0012CP0762	8			-2.34	-1.76	-0.86	-0.45	0.12	0	-0.77	-0.52
CPn0965CP0895lpxB	8			-0.97	-1.03	-0.69	-0.52	0.04	0	-0.09	-0.43
CPn1029CP0823	8			-2.28	-1.95	-1.59	-0.95	0.17	0	-0.24	-0.15
CPn1000CP0854rs15	8			-0.53	-0.19	-0.49	-0.59	-0.24	0	-0.43	-0.13
CPn0921CP0945	8			-1.89	-0.41	-0.53	-0.94	-0.21	0	-0.72	-0.76
CPn1047CP0805dapB	8			-1.13	-0.98	-0.77	-0.52	-0.34	0	0.26	-0.2
CPn0939CP0922	8		Yes	-2.64	-1.88	-1.09	-1.11	-0.1	0	-0.41	0.22
CPn0909CP0957rsbV2	8	UP		-2.3	-1.1	-1.07	-0.73	0.36	0	-0.11	-0.23
CPn0863CP1006pgmA	8		Yes	-2.53	-1.48	-1.3	-1.02	-0.02	0	-0.11	-0.12
CPn0874CP0995	8			-0.76	-0.53	-0.49	-0.22	-0.21	0	0.24	0.02
CPn0870CP0999serS	8	DOWN	Yes	-1.9	-0.89	-0.83	-0.61	-0.36	0	-0.08	0.02
CPn0896CP0970	8			-1.35	-0.78	-0.47	-0.61	-0.31	0	0.24	0.2
CPn0884CP0982	8	DOWN		-1.69	-0.83	-0.79	-0.38	-0.04	0	0	-0.24
CPn0905CP0961murC/ddlA	8		Yes	-1.61	-1.1	-0.7	-0.26	0.23	0	-0.37	-0.43
CPn0899CP0967murF	8			-1.33	-0.56	-0.3	-0.46	-0.51	0	-0.01	-0.53
CPn0059CP0716dut	8			-0.76	-0.59	-0.65	-0.32	-0.25	0	-0.28	-0.2
CPn0847CP1022clpP2	8		Yes	-1.29	-0.5	-0.58	-0.21	0.01	0	-0.11	-0.31
CPn0708CP0038	8	DOWN		-1.88	-0.99	-0.87	-1.12	0.05	0	-0.24	-0.2
CPn0709	8			-2.74	-1.72	-1.77	-1.69	-0.7	0	0.02	0.37
CPn0710CP0036	8		Yes	-2.85	-1.97	-1.62	-1.71	-1.12	0	-0.65	0.12
CPn0711CP0035	8	DOWN	Yes	-3.39	-1.28	-1.39	-1.47	-0.87	0	-0.14	0.16
CPn0705CP0041	8	DOWN	Yes	-2.35	-1.1	-1.04	-1.12	-0.86	0	-0.73	-0.14
CPn0712CP0034	8	DOWN		-2.17	-0.34	-0.51	-1.15	-0.59	0	-0.5	-0.1
CPn0707CP0039yscN	8	DOWN	Yes	-2.28	-0.76	-0.56	-1.02	-0.64	0	-0.33	-0.17
CPn0713CP0033	8		Yes	-2.08	-0.92	-0.94	-1.34	-0.18	0	0.45	0.15
CPn0004CP0771gatB	7	UP	Yes	-1.34	-0.66	-0.05	0.1	0.4	0	-0.33	-0.24
CPn0263CP0498yqfU	7	UP		-1.05	-0.25	-0.48	-0.11	0.38	0	-0.19	-0.25
CPn0912CP0954	7		Yes	-1.96	-1.1	-0.28	0.11	0.45	0	-0.11	-0.38
CPn0600CP0148	7			-1.15	-0.37	-0.45	-0.3	0.2	0	-0.21	-0.12
CPn0402CP0353mutY	7			-0.69	-0.03	-0.22	-0.36	-0.01	0	-0.12	0.03
CPn0101CP0673ybbP	7			-0.74	0	0.19	-0.23	-0.01	0	0.15	0.38
CPn0668	7			-1.64	-0.25	0	-0.45	0.48	0	0.2	-0.03
CPn0401CP0354	7			-0.72	-0.04	0.22	-0.07	0.04	0	-0.42	0.12
CPn0762CP1110	7		Yes	-2.25	-0.23	-0.48	-0.26	0.18	0	-0.62	-0.28
CPn0275CP0484gyrB1	7			-1.4	0.19	-0.13	-0.31	-0.32	0	-0.5	-0.29
CPn0276CP0483	7			-1.48	0	-0.34	-0.24	0.1	0	0.01	0.23
CPn0304CP0454pdhA	7		Yes	-0.96	-0.21	-0.06	-0.05	0.37	0	0.05	-0.37
CPn0985CP0871nrdB	7	DOWN		-1.8	-0.45	-0.44	-0.84	0.13	0	0.09	0.14
CPn0309CP0449dnaA1	7			-0.4	0.03	-0.12	-0.19	-0.04	0	0.01	-0.34
CPn0338CP0419dnaN	7		Yes	-1.53	-0.07	-0.01	-0.22	0.28	0	0	-0.09
CPn0300CP0458yaeT	7		Yes	-2.11	-0.59	-0.56	-0.15	0.14	0	-0.13	-0.43
CPn0940CP0921uvrC	7			-0.75	-0.19	-0.14	-0.49	0.73	0	-0.05	0.1
CPn0297CP0461fabD	7		Yes	-1.5	-0.63	-0.29	-0.11	0.47	0	-0.39	-0.31
CPn0521CP0232glyA	7			-1.86	-0.23	-0.08	-0.04	0.45	0	-0.09	0
CPn0409CP0345	7			-1.03	-0.39	-0.08	-0.18	0.06	0	-0.33	-0.21
CPn0094CP0680valS	7			-0.78	0.02	-0.14	0.03	-0.21	0	-0.02	-0.02

CPn0520CP0233clpP1	7		Yes	-2.04	-0.41	-0.23	-0.39	-0.04	0	-0.47	-0.14
CPn0185CP0583rpe	7			-0.95	0.08	-0.21	-0.09	-0.36	0	0.26	0.26
CPn0832CP1038lipA	7			-1.42	-0.48	-0.46	-0.42	0.31	0	-0.26	-0.41
CPn0889CP0977hemN2	7			-0.97	-0.15	0.12	0.08	0.45	0	-0.09	-0.22
CPn1049CP0803lysC	7			-1.36	-0.4	-0.09	-0.25	0.06	0	-0.13	-0.35
CPn0424CP0329dnaA2	7			-1.95	-0.37	-0.32	-0.47	-0.23	0	-0.29	-0.25
CPn0448CP0305yxjG2	7			-0.47	-0.27	0.25	0.03	-0.09	0	-0.21	0.02
CPn0126CP0646	7			-0.59	-0.23	0.11	-0.07	0.36	0	-0.09	0.07
CPn0425CP0328	7			-1.93	-0.56	-0.42	-0.37	0.11	0	-0.29	-0.09
CPn0984CP0872nrdA	7	DOWN		-2.7	-0.19	-0.33	-0.66	-0.48	0	0.13	0.44
CPn0721kdsA	7			-0.87	0.04	0.02	-0.26	0.23	0	-0.04	0.09
CPn0906CP0960	7			-2.14	-0.18	-0.39	-0.45	0.24	0	-0.65	-0.23
CPn0183CP0585accB	7		Yes	-1.17	-0.15	-0.32	-0.33	0.07	0	-0.25	0.07
CPn1031CP0821arcD	7	DOWN		-3.43	-0.18	0.29	1.9	-2.31	0	-0.58	0.18
CPn0716CP0030	6	DOWN		-0.67	0.54	0.32	0.17	0.32	0	-0.51	-0.48
CPn0549CP0203rs10	6			-0.29	0.41	0.07	-0.07	0.49	0	-0.59	-0.64
CPn0551_rs7	6			-0.2	0.24	0.08	-0.17	0.31	0	-0.76	-0.65
CPn0914CP0952	6			-0.64	0.22	0.01	0.33	0.08	0	-0.12	-0.53
CPn0052CP0723hemC	6			-0.21	-0.07	-0.12	0.15	0.26	0	-0.28	-0.5
CPn0411CP0343	6			-0.28	0.41	0.61	0.76	-0.27	0	-0.8	-1
CPn0968CP0892	6			-0.66	0.26	0.01	0.23	0.13	0	-0.68	-0.65
CPn0650CP0097lpxA	6			-0.54	0.35	0.2	0.27	-0.25	0	-0.8	-0.76
CPn0658	6		Yes	-0.46	0.22	0.12	-0.21	0.51	0	-0.28	-0.65
CPn0880CP0989ftsK	6			-0.3	-0.12	0.16	0.09	0.08	0	-0.12	-0.38
CPn0487CP0267	6	DOWN		-0.54	0.22	0.23	0.05	-0.06	0	-0.36	-0.43
CPn0518CP0235	6		Yes	-0.73	1.02	0.21	0.1	0.32	0	-0.35	-0.7
CPn0077CP0698rl11	6			-0.04	0.34	-0.09	-0.15	0.23	0	-0.59	-0.46
CPn0900CP0966mraY	6			-0.08	-0.01	0.25	0.1	0.03	0	-0.17	-0.05
CPn0917CP0949	6		Yes	-0.84	0.46	0.42	0.17	0.56	0	-0.63	-0.57
CPn0891CP0975mfd	6			-0.21	0.29	0.45	0.11	-0.01	0	0.04	-0.63
CPn0532CP0220ribC	6			-0.82	0.28	-0.2	0.1	0.01	0	-0.49	-0.7
CPn0945CP0914	6			0.08	0.35	-0.17	0.03	-0.05	0	-0.86	-0.35
CPn0903CP0963ftsW	6			-0.1	-0.09	-0.08	0.42	-0.3	0	-0.15	-0.62
CPn0952CP0907rs18	6			-0.26	0.16	0.01	0.05	0.41	0	-1.07	-0.73
CPn0802CP1069trpS	6			-0.23	0.12	0.52	0.17	0.08	0	-0.21	-0.67
CPn0639CP0108rl29	6			-0.24	0.56	0.19	0.11	0.45	0	-0.76	-0.32
CPn0563CP0187recJ	6			0	-0.11	0.01	0.25	0.25	0	0.04	-0.27
CPn0612CP0135polA	6			-1.03	0.11	0.3	0.21	0.44	0	-0.87	-0.91
CPn0247CP0515rl13	6			-0.31	0.54	0	-0.13	-0.15	0	-1.06	-0.67
CPn0738CP0007	6			-0.04	-0.04	-0.2	0.02	0.17	0	-0.31	-0.46
CPn0613CP0134sohB	6			-0.48	-0.03	0.4	0.23	0.43	0	-0.2	-0.54
CPn0740tyrB	6			-0.37	0.63	0.19	0.22	0.06	0	-0.84	-0.57
CPn1022CP0831	6			-0.78	0.44	0.98	0.57	0.63	0	-0.21	-0.5
CPn0736CP0009ygeD	6			-0.03	0.03	0.17	0.17	0.22	0	-0.45	-0.78
CPn1021CP0832lcrH2	6			-0.55	0.47	1.19	0.52	0.13	0	-0.87	-0.8
CPn1017CP0836lytB	6			-0.8	-0.17	-0.02	0.09	0.34	0	-0.69	-0.74
CPn0603CP0144hemZ	6			-0.19	-0.18	0.42	0.26	-0.28	0	-0.77	-0.86
CPn0619CP0128ndk	6		Yes	-0.83	0.49	0.48	0.52	0.14	0	-0.62	-0.72
CPn0733CP0013rs4	6			-0.36	0.83	-0.21	-0.2	0.37	0	-0.74	-0.66
CPn0756CP1116rpoD	6			-0.41	0.43	0.54	0.37	-0.08	0	-0.27	-0.86
CPn0638CP0109rs17	6			-0.17	0.3	0.08	0.27	0.13	0	-0.32	-0.46
CPn1068CP0782rnhB2	6			-0.15	0.28	0.16	0.06	0.07	0	-0.66	-0.43
CPn0640CP0107rl16	6			-0.31	0.04	0.23	0.17	0.36	0	-0.54	-0.37
CPn0729CP0017	6			-1.15	0.59	0.81	0.68	0.73	0	-0.48	-0.74
CPn0186CP0581	6			-0.31	-0.35	0.27	0.21	0.4	0	-0.73	0.08
CPn1060CP0790tktB2	6			-0.18	0.07	0.4	0.07	0.36	0	-0.52	-0.37

CPn0626CP0121rpoA	6		Yes	-0.18	0.56	0.34	0.04	-0.14	0	-0.85	-0.59
CPn0631CP0116rs5	6	DOWN		0.12	-0.02	0.29	0.21	0.12	0	-0.55	-0.56
CPn0692CP0054	6			-0.15	0.15	0.44	0.25	0.09	0	-0.21	-0.47
CPn1042CP0810bioD	6			-1.02	0.65	0.83	0.52	0.26	0	-0.35	-0.5
CPn0753CP1119	6	UP		-0.06	-0.04	0.37	0.03	0.38	0	-0.62	-0.37
CPn0605CP0142yhhF	6			-0.62	0.41	0.72	0.24	0.11	0	-0.56	-0.82
CPn0990CP0865infC	6	UP		-0.33	0.33	0.14	0.05	0.26	0	-0.21	-0.4
CPn0569CP0180plsC	6			-0.56	0.55	-0.01	0.18	0.42	0	-0.51	-0.61
CPn0298CP0460fabH	6		Yes	-0.15	-0.06	0.04	0	0.1	0	-0.14	-0.22
CPn0991CP0864rl35	6			-0.5	0.65	0.02	0.07	0.29	0	-0.67	-0.68
CPn0574CP0175	6			-0.63	0.12	0.69	0.24	0.4	0	-0.47	-0.51
CPn0758CP1114folP	6		Yes	-1	0.21	0.29	0.45	0.56	0	-0.66	-0.85
CPn0316CP0441nusA	6		Yes	-0.02	0.23	-0.1	0.11	-0.18	0	-0.67	-0.49
CPn0327CP0430rl28	5			-0.02	0.41	0.18	0.12	0.45	0	-0.06	-0.43
CPn0317CP0440infB	5	DOWN		-0.09	0.86	0.18	0.32	0.46	0	-0.73	-0.57
CPn0428CP0325nqr3	5			0.27	0.02	0.14	0.28	-0.23	0	-0.27	-0.29
CPn0848CP1021tigA	5		Yes	0.15	1.01	0.87	1.12	0.45	0	-0.68	-0.59
CPn0966CP0894pcnB2	5			0.04	0.61	0.49	0.06	0.06	0	-0.55	-0.7
CPn0890CP0976hemE	5			-0.23	0.31	0.55	0.46	0.35	0	0	-0.63
CPn1044CP0808bioB	5			-0.58	0.78	0.75	0.72	0.52	0	-0.14	-0.26
CPn1043CP0809bioF2	5			-0.26	0.63	0.97	0.62	0.07	0	-0.1	-0.6
CPn0440CP0313	5			0.64	1.74	0.93	1.57	-0.13	0	-0.84	-0.88
CPn0321CP0436ychF	5		Yes	-0.39	0.79	0.04	0.29	0.15	0	-0.5	-0.45
CPn0312CP0446	5			0.31	0.49	-0.11	0.09	0.26	0	-0.33	-0.18
CPn0892CP0974alaS	5			-0.17	0.21	0.74	0.6	0.34	0	-0.23	-0.32
CPn0688CP0058	5			0.26	0.16	0.21	0.29	-0.23	0	-0.46	-0.68
CPn0643CP0104rs19	5			0.1	0.61	0.44	0.47	0.46	0	-0.64	-0.15
CPn0916CP0950fabF	5		Yes	0.19	0.11	0.03	0.43	0.33	0	-0.17	-0.4
CPn0989CP0866	5			0.54	0.46	0.46	0.38	-0.07	0	-0.7	-0.41
CPn0235CP0527kdsB	5		Yes	0.1	0.06	0	0.23	0.05	0	-0.37	0.05
CPn0283	5			0.21	0.45	0.09	-0.03	0.02	0	-0.42	-0.22
CPn0993CP0862pheS	5			0.02	1.08	0.75	0.65	-0.21	0	-0.27	-0.53
CPn0279CP0479	5	UP		-0.38	0.65	0.46	0.43	0.35	0	-0.1	-0.07
CPn1015CP0838	5	UP		-0.21	0.5	0.18	0.26	-0.2	0	-0.21	-0.47
CPn0992CP0863	5			0.12	1.01	0.4	0.17	0.54	0	-0.44	-0.67
CPn0915CP0951ybeB	5			0.18	0.07	0.18	0.25	-0.21	0	-0.52	-0.47
CPn0318CP0439rbfA	5	DOWN		-0.07	1.53	0.5	0.39	0.5	0	-0.78	-0.77
CPn0289CP0469	5			-0.42	0.77	0.57	0.42	0.41	0	-0.34	-0.29
CPn0364CP0393fer4	5			-0.24	0.45	0.51	0.54	0.15	0	-0.15	-0.24
CPn0238CP0524zwf	5			0.13	0.32	0.23	0.17	0.53	0	-0.3	-0.31
CPn0346CP0414ytgD	5			0.42	0.67	0.34	0.12	0.23	0	-0.58	-0.26
CPn0953CP0906rl9	5		Yes	0.29	0.8	0.39	0.35	0.63	0	-0.62	-0.56
CPn0251CP0510	5			-0.3	1.29	0.34	0.58	0.27	0	-0.38	-0.72
CPn0690CP0056	5		Yes	0.02	0.45	0.56	0.26	0.39	0	-0.34	-0.61
CPn0352CP0407	5			0.21	-0.12	0.27	0.37	0.18	0	-0.29	-0.39
CPn0950CP0909pth	5			0.4	1.48	0.72	0.58	-0.09	0	-0.82	-0.38
CPn0121CP0652	5		Yes	-0.22	0.99	0.37	0.15	0.51	0	-0.43	-0.35
CPn0644CP0103rl2	5			0.27	0.38	0.61	0.09	0.49	0	-0.72	-0.28
CPn0091CP0683atpI	5			0.18	0.91	0.61	0.63	0.32	0	-0.54	-0.34
CPn0081CP0694rpoB	5	DOWN		0.36	1.27	1.09	0.61	0.94	0	-0.9	-0.72
CPn0115CP0658ffh	5		Yes	0.38	1.13	0.25	0.36	0.29	0	-0.78	-0.41
CPn0550fusA	5		Yes	-0.02	1.09	0.36	0.64	0.54	0	-0.76	-0.66
CPn0079CP0696rl10	5		Yes	0.53	0.48	0.09	0.26	-0.08	0	-0.7	-0.42
CPn0575CP0174yhhY	5			-0.32	0.67	0.43	0.53	-0.3	0	-0.54	-0.29
CPn0647CP0100rl3	5			-0.07	0.5	0.39	0.22	-0.02	0	-0.37	0.04
CPn0645CP0102rl23	5			-0.07	0.25	0.23	0.21	0.52	0	-0.31	-0.17

Cpn0548_CP0204	5			0.37	0.82	0.71	0.54	0.7	0	-0.6	-0.82
CPn0611CP0136yacE	5		Yes	0.08	0.45	0.35	0.52	-0.02	0	-0.16	-0.34
CPn0759CP1113folA	5			0.14	0.62	0.83	0.63	0.38	0	-0.46	-0.77
CPn0504CP0249vacB	5			0.56	0.68	0.08	0.26	0.14	0	-0.54	-0.62
CPn0088CP0686atpA	5			-0.05	0.92	0.38	0.45	0.41	0	-0.47	-0.19
CPn0075CP0700secE	5			-0.03	1.06	0.08	0.52	-0.01	0	-0.26	-0.67
CPn0734yceA	5			0.05	0.45	0.36	0.38	0.53	0	-0.32	-0.49
CPn0076CP0699nusG	5		Yes	0.1	0.92	0.45	0.31	0.25	0	-0.64	-0.31
CPn0539CP0213pmp19	5	UP		-0.32	0.76	0.81	0.53	0.05	0	-0.03	-0.45
CPn0632CP0115rl18	5	DOWN		0.05	0.26	0.33	0.29	0.62	0	-0.6	-0.6
CPn0635CP0112rl5	5			-0.14	0.32	0.22	0.28	0.3	0	-0.57	-0.26
CPn0050	5			0.51	0.88	0.04	-0.46	-0.07	0	-0.67	-0.6
CPn0575aprfB	5			-0.35	0.74	0.57	0.39	-0.16	0	-0.37	-0.38
CPn0634CP0113rs8	5	DOWN		0.04	0.47	0.34	0.27	0.14	0	-0.72	-0.38
CPn0633CP0114rl6	5	DOWN		0.13	0.97	0.45	0.44	0.26	0	-0.7	-0.51
CPn0630CP0117rl15	5			0.02	0.58	0.2	0.67	0.04	0	-0.69	-0.55
CPn0625CP0122rl17	5			-0.02	0.61	0.55	0.16	0.1	0	-0.68	-0.52
CPn0646CP0101rl4	5			-0.1	0.6	0.27	0.42	-0.1	0	-0.63	-0.43
CPn0543CP0209	5			-0.01	1.32	0.69	0.36	0.29	0	-0.56	-0.77
CPn0116CP0657rs16	5			0.26	0.61	0.38	0.17	0.44	0	-0.72	-0.62
CPn0628CP0119rs13	5		Yes	0.07	0.7	0.54	0.17	0.45	0	-0.74	-0.45
CPn0092CP0682atpK	5			0.04	0.32	0.47	0.47	0.47	0	-0.4	-0.26
CPn0570CP0179	4			0.1	0.26	0.72	0.58	0.59	0	0.04	-0.61
CPn0972CP0887ftsY	4		Yes	0.07	0.24	0.79	0.14	0.6	0	-0.04	-0.54
CPn0093CP0681	4			-0.05	0.61	0.52	0.32	0.37	0	-0.13	-0.22
CPn0344CP0416yaeL	4			-0.06	0.36	0.2	-0.02	-0.18	0	0.15	-0.22
CPn0351CP0408adt1	4			0.57	1.09	0.08	-0.1	0.26	0	0.07	-0.11
CPn0542CP0210	4			0.86	0.84	0.59	0.71	0.06	0	-0.2	-0.33
CPn0544CP0208yhbZ	4			0.62	0.97	0.37	0.14	0.42	0	0	-0.23
CPn0983CP0873	4			0.14	0.25	0.35	0.09	0.25	0	0.18	-0.48
CPn0649CP0098fmt	4	DOWN		0.51	1.32	1.06	0.58	0.23	0	-0.02	-0.4
CPn0078CP0697rl1	4			0.21	0.4	0.3	0.28	0.46	0	-0.16	-0.07
CPn0055CP0720	4			0.39	0.47	0.5	0.37	0.09	0	-0.43	-0.29
CPn0560CP0190gltX	4			0.6	1.09	1.11	0.68	0.39	0	-0.08	-0.39
CPn0319CP0438truB	4			0.12	0.27	0.37	-0.03	0.54	0	0	-0.07
CPn0120CP0653gmk	4		Yes	0.29	1.06	0.4	0.32	0.59	0	-0.49	-0.15
CPn0689CP0057yfhO1	4			0.43	0.98	0.93	0.75	0.58	0	-0.14	-0.3
CPn0058CP0717accD	4			0.58	0.8	0.08	0.15	0.49	0	-0.32	-0.34
CPn0578CP0170yaeI	4			-0.09	0.17	0.67	0.5	0.62	0	0.12	-0.38
CPn1040CP0812	4		Yes	0.81	0.94	0.96	1.02	0.29	0	-0.14	-0.49
CPn0237CP0525yggF	4			0.46	0.82	0.51	0.57	0.41	0	-0.48	-0.27
CPn0236CP0526pyrG	4			0.76	0.53	0.42	0.56	0.41	0	-0.5	-0.19
CPn0629CP0118secY	4	DOWN		0.41	0.77	0.75	0.52	0.17	0	-0.25	-0.33
CPn0230CP0534	4			0.21	0.4	0.24	0.23	0.42	0	-0.16	-0.11
CPn0080CP0695rl7	4		Yes	0.89	0.84	0.16	0.16	0.21	0	-0.36	-0.58
CPn1051CP0801	4			0.22	0.34	0.39	0.6	0.09	0	-0.15	-0.51
CPn1059CP0791kgsA	4			0.36	0.86	0.53	0.31	-0.09	0	-0.11	-0.82
CPj0954Cpn0954	4			0.81	0.74	0.87	0.65	0.75	0	-0.23	-0.22
CPn0739CP0006	4			0.89	1	0.78	0.23	0.4	0	0.36	-0.34
CPn0617CP0130gidA	4			1.06	1.37	1.1	0.73	0.84	0	-0.21	-0.4
Cpn0090_CP0684	4			0.22	0.77	0.93	0.85	0.22	0	-0.5	-0.27
CPn0087CP0687	4			0.58	1.11	0.71	0.72	0.36	0	-0.18	-0.18
CPn0290CP0468	4			0.05	1.07	0.91	0.68	0.5	0	0.25	-0.04
CPn0089CP0685atpB	4		Yes	0.26	1.18	0.77	0.49	0.37	0	-0.18	-0.33
CPn0598CP0150oppB2	4			0.31	0.36	0.13	-0.06	0.59	0	-0.11	-0.48
CPn0597CP0151oppC2	4			0.36	0.52	0.64	0.64	0.26	0	-0.41	-0.32

CPn0741CP0004	4			0.43	1.36	0.62	0.71	0.28	0	-0.54	-0.59
CPn0601CP0146	4			0.17	0.43	0.86	0.45	0.66	0	-0.17	-0.24
CPn0003CP0772gatA	4		Yes	0.18	0.65	0.67	0.78	0.37	0	-0.29	-0.33
CPn0359CP0399lepA	4			0.58	0.64	0.67	0.47	0.24	0	-0.53	-0.3
CPn0961CP0899rl32	4	UP		0.56	1.35	0.75	0.49	0.7	0	-0.11	-0.46
CPn0674CP0073fmu	4			0.64	1.65	1.56	0.78	0.16	0	-0.31	-0.29
CPn0109CP0665ileS	4			0.01	0.31	0.38	0.14	0.37	0	-0.21	0
CPn0119CP0654rnhB1	4			0.63	0.87	0.49	0.43	0.45	0	-0.35	-0.33
CPn0942CP0919dnaG	4			0.13	0.07	0.23	0.32	0.36	0	0.19	-0.35
CPn0122CP0651metG	4		Yes	0.51	0.83	0.36	0.53	0.15	0	-0.41	-0.41
CPn0412CP0342	4			0.84	0.81	0.59	0.49	0.35	0	-0.42	-0.53
CPn0476CP0278	4			0.78	0.78	0.8	0.44	0.54	0	-0.15	-0.29
CPn0410CP0344dnaQ1	4			0.21	0.32	0.26	0.21	0.25	0	-0.15	-0.29
CPn0910CP0956miaA	4			0.13	0.35	0.5	0.38	0.45	0	0	-0.17
CPn0719CP0027sfhB	4			0.27	0.39	0.65	0.32	0.41	0	-0.2	-0.17
CPn0829CP1042	4	UP		0.35	0.41	0.14	0.33	-0.06	0	0.17	-0.5
CPn0118CP0655rl19	4			0.45	1.25	0.59	0.64	0.35	0	-0.47	-0.22
CPn0397CP0358	4			0.33	0.86	0.76	0.33	0.49	0	-0.17	-0.11
CPn0839CP1030psdD	4			0.4	0.93	0.55	0.47	0.23	0	-0.15	-0.39
CPn0114CP0659hemK	4			0.54	0.81	0.55	0.58	-0.03	0	-0.18	-0.08
CPn0071CP0704	4			0.09	0.52	0.2	0.38	-0.28	0	0.1	-0.13
CPn0037CP0738ptsH	4			0.16	0.39	0.48	0.19	0.2	0	-0.16	-0.04
CPn0032CP0744dnaJ	4		Yes	0.95	1.29	0.44	0.3	0.3	0	-0.17	-0.22
CPn0117CP0656trmD	4			0.57	1.27	0.83	0.69	0.65	0	-0.41	-0.48
CPn0836CP1033brnQ	4			0.17	0.55	0.45	0.3	0.13	0	-0.15	-0.17
CPn0835CP1034	4			0.74	1.72	0.97	0.78	0.46	0	-0.28	-0.82
CPn0527CP0225sucB2	4		Yes	0.56	0.64	0.37	0.55	0.13	0	-0.27	-0.45
CPn0655CP0092dnaQ2	4	DOWN		0.54	0.99	0.85	0.45	-0.04	0	-0.25	-0.35
CPn0801CP1070	4			0.38	0.63	0.58	0.66	0.62	0	-0.08	-0.4
CPn0023CP0753yjjK	4		Yes	0.71	1.01	0.64	0.62	0.1	0	-0.42	-0.32
CPn0927CP0939	3			1.5	1.22	1.51	1.02	-0.07	0	-0.03	-0.22
CPn1003CP0850	3			0.15	0.18	0.48	0.55	-0.1	0	0.5	-0.46
CPn0145CP0628	3			0.56	0.38	0.67	0.31	0.1	0	0.27	0.13
CPj05762CPn0576	3			0.41	-0.02	1.55	0.79	0.08	0	0.12	0.28
CPn0686CP0060	3			0.7	0.39	0.58	0.8	0.41	0	0.16	0.01
CPn0541CP0211	3			0.54	0.71	0.78	0.36	0.39	0	0.12	0.06
CPn1063CP0786tpiS	3	UP	Yes	0.76	0.7	0.68	0.46	0.38	0	0.1	0.04
CPn0589CP0159	3	DOWN		0.09	0.37	0.58	0.56	-0.21	0	0.06	0.01
CPn0456	3			1.13	0.42	0.79	0.68	0.39	0	0.39	-0.06
CPn0025CP0751atsA	3	UP		1.2	1.57	1.44	1.11	0.32	0	0.39	-0.05
CPn0264CP0497ubiD	3	UP		0.59	0.56	1.15	0.69	0.19	0	0.55	0.19
CPn0746CP1126	3		Yes	0.28	0.07	0.44	0.16	0.23	0	0.32	-0.46
CPn0150CP0623	3			1.63	0.31	1.78	1.06	0.05	0	0.18	0.21
CPn0934CP0927rnpA	3			0.53	0.31	0.73	0.16	0.06	0	0.25	0.06
CPn0358CP0400	3	UP		0.68	0.06	0.95	-0.14	0.63	0	0.16	-0.37
CPn0871CP0998ribD	3	UP		0.57	0.25	0.75	0.56	0.33	0	0.4	-0.08
CPn0641CP0106rs3	3			0.49	0.35	0.34	0.45	0.58	0	0.38	0.17
CPn0147CP0626	3			0.05	0.52	1.05	0.58	0.27	0	-0.22	0.21
CPn0627CP0120rs11	3			0.23	0.31	0.43	0.36	-0.01	0	-0.3	0.15
CPn0881CP0986	3			1.54	1.14	1.93	1.73	0.24	0	0.21	-0.5
CPn1065	3	UP		0.64	-0.12	0.63	0.9	-0.38	0	0.36	-0.05
CPn0026CP0750	3			1.86	1.65	2.01	1.2	0.74	0	0.36	0.06
CPn0356CP0402	3			0.32	0.03	0.96	0.6	0.28	0	0.24	-0.08
CPn0146CP0627	3			1.16	1.2	1.26	0.95	0.04	0	0.04	0.14
CPn0491CP0263	3			0.73	0.27	0.68	0.49	-0.04	0	0.22	0.01
CPn0068CP0706	3			1.79	3.21	3.31	1.54	-0.12	0	0.42	1.83

CPn0463CP0289	3			1.41	0.25	1.47	0.85	0.33	0	1.05	0.06
CPn0379CP0377	3			0.38	1.2	1.09	0.77	0.05	0	-0.03	0.6
CPn0418CP0336murE	3	UP		1.82	1.9	2.13	1.36	0.21	0	0.18	0.28
CPn0554CP0198	3	UP		1.12	0.54	1.04	0.63	0.55	0	0.08	0
CPn0191CP0576glnQ	3			0.17	0.7	0.93	0.79	0.42	0	0.26	0.2
CPn0315CP0442	3		Yes	0.51	0.04	0	1.52	0	0	-0.03	-0.05
CPn0530CP0222spoU1	3			0.47	0.4	0.77	0.73	-0.15	0	0.42	-0.2
CPn0396CP0359yhfO	3			1.19	1.02	1.25	0.78	0.46	0	0	0.13
CPn0211CP0554	3	UP		1.44	0.61	1.87	0.84	0.21	0	0.23	0.44
CPn0054CP0721rnc	3		Yes	0.46	0.32	0.51	0.51	0.48	0	0.12	-0.04
CPn0813CP1058pepP	3		Yes	0.1	-0.34	0.65	0.38	0.5	0	0.26	0.15
CPn0723yhbG	3	UP		0.69	0.89	1.14	0.62	0.29	0	-0.11	0.36
CPn1054CP0764	3		Yes	0.28	-0.31	0.52	0.55	0.36	0	-0.21	-0.23
CPn0986CP0869yggH	3	UP		0.22	0.37	1.02	0.67	0.1	0	0.15	-0.22
CPn1053CP0799	3			0.66	1.34	1.37	0.66	0.31	0	-0.05	0.02
CPn0979CP0877htrA	3	UP	Yes	-0.12	-0.18	0.88	0.03	0.45	0	-0.13	0.27
CPn0292CP0466incC	3			-0.03	0.24	0.61	0.67	0.33	0	-0.16	-0.01
CPn0051	3			0.47	0.14	0.74	-0.05	-0.13	0	0.03	0.01
CPn0680CP0067ygo4	3			0.9	0.61	0.92	0.4	0.18	0	0.46	-0.22
CPn0787CP1085yabD	3	UP		1.03	0.89	1.42	1.03	0.29	0	0.28	0.09
CPn0962CP0898plsX	3			0.2	0.75	0.81	0.49	0.09	0	0.17	0.27
CPn0975CP0882	3			0.37	0.32	0.51	0.39	-0.38	0	-0.14	0.3
CPn0867CP1002rodA	3	UP		0.88	0.21	1.03	0.7	0.28	0	0.41	0.55
CPn0043CP0730	3	UP		1.28	0.87	1.37	0.76	0.27	0	0.38	0.29
CPn1052CP0800	3	UP		0.19	4.02	3.33	1.8	-0.45	0	0.45	0.76
CPn0403yceC	3	UP		1.15	0.72	1.31	0.65	0.63	0	0.5	0.17
CPn0924CP0942priA	3			1.2	0.96	1.31	0.63	0.23	0	0.31	-0.15
CPn0035CP0741	2	UP		3.28	2.31	1.74	1.15	0.08	0	0.53	0.52
CPn0066CP0708	2	UP		2.49	1.92	1.74	1.24	0.06	0	0.74	0.81
CPn0187CP0580	2			2.28	1.52	1.2	0.88	0.47	0	0.67	0.65
CPn0020CP0756	2	UP		1.82	0.63	1.17	1.06	0.18	0	0.09	0.19
CPn0030CP0746gcp1	2			2.87	1.52	0.76	0.29	0.31	0	0.19	0.24
CPn0024CP0752xerC	2	UP		2.88	1.28	1.3	1.2	0.17	0	0.49	0.45
CPn0049CP0725	2			0.84	0.86	-0.22	0.16	0.08	0	-0.27	-0.07
CPn0095CP0679	2			0.64	0.3	0.6	0.24	0.02	0	0.02	0.19
CPn0098htrB	2			2.9	1.74	0.62	0.68	-0.17	0	0.64	0.6
CPn0034CP0742	2	UP		0.63	0.24	0.45	0.34	0.41	0	0.3	0.25
CPn0102CP0672cydA	2	UP		2.12	1.23	1.09	0.8	0.44	0	0.45	0.49
CPn0152CP0620	2			0.95	0.89	0.01	0.51	0.36	0	-0.1	0.06
CPn0149CP0624dnlJ	2	UP		3.14	2.44	1.23	1.34	0.41	0	0.88	0.86
Cpn0019_pmp52	2			1.53	0.89	1.13	0.93	-0.41	0	0.9	0.15
CPn0046CP0728	2	UP		1.57	0.98	1.11	0.75	0.19	0	0.01	0.08
CPn0163CP0608	2	UP		1.69	0.32	0.52	0.62	0.03	0	0.46	0.75
CPn0041CP0733	2			1.36	0.33	0.23	0.61	-0.24	0	0.21	0.3
CPn0113CP0660pfrA	2		Yes	1.11	1.07	1.01	0.39	0.62	0	0.16	-0.15
CPn0006	2	UP		2.22	1.25	1.31	0.67	0.31	0	1.01	0.56
CPn0182CP0586accC	2			1.2	0.93	0.44	0.73	0.21	0	-0.08	0.04
CPn0036CP0739	2			1.04	0.9	0.89	0.69	0.23	0	0.15	0.15
CPn0038CP0737ptsI	2	UP		2.45	1.15	1.76	0.51	0.13	0	0.05	0.82
CPn0008CP0766	2	UP		1.26	0.71	0.72	0.24	0.71	0	0.02	0.19
CPn0009CP0765	2	UP		1.33	0.89	1.38	0.39	0.92	0	0.6	0.58
CPn0086_CP0688	2	UP		2.11	1.68	1.18	1	0.35	0	0.17	0.3
CPn0168CP0603	2	UP		2.45	1.03	1.37	0.68	0.01	0	0.15	0.68
CPn0167CP0604	2			1.56	0.63	0.63	0.3	-0.23	0	0.2	0.51
CPn0028CP0748	2	UP		1.89	1.05	1.02	0.66	0.15	0	0.29	0.27
CPn1073CP0776	2	UP		2.45	1.25	1.13	0.74	0.17	0	0.29	0.44

CPn0196CP0571oppA2	2			1.3	1.19	0.61	0.17	-0.36	0	0.63	0.24
CPn0197CP0570oppA3	2			1.03	0.93	0.45	0.39	0.04	0	0.23	0.27
CPn0618CP0129lplA2	2	UP		2.29	1.69	1.48	1.2	0.18	0	0.79	0.29
CPn0593CP0155	2			1.57	0.6	0.75	0.35	0.14	0	0.46	0.33
CPn0596CP0152ada	2			2.35	1.16	1.41	1.03	0.14	0	0.64	0.2
CPn0614CP0133adt2	2			1.96	1.53	1.38	0.93	0.16	0	-0.24	0.16
CPn0615CP0132pgsA1	2			1.57	1.09	0.42	0.33	0.04	0	0.34	0.08
CPn0620CP0127ruvA	2	UP		1.67	0.61	1.05	0.64	0.29	0	0.48	0.33
CPn0588CP0160	2	UP		2.76	1.21	1.31	0.75	-0.14	0	0.71	1.05
CPn0648CP0099	2			2.39	1.79	1.4	0.93	0.68	0	-0.07	0.4
CPn0656CP0091	2			0.8	0.64	0.19	-0.41	-0.08	0	-0.27	0.07
CPn0673CP0074	2			2.4	1.42	1.58	0.68	0	0	0.43	0.16
CPn0679CP0068	2	UP	Yes	2.02	1.47	1.84	0.94	0.7	0	0.4	0.5
CPn0590CP0158	2			0.66	0.72	0.34	0.02	0.05	0	0.44	0.1
CPn0580CP0168truA	2	UP		1.84	1.23	1.42	0.73	0.49	0	0.06	0.29
CPn0585CP0163	2			1.64	1.46	1.22	0.46	0.18	0	-0.11	0.11
CPn0715CP0031gyrB2	2			2.39	2.08	1.15	0.93	0.21	0	0.47	0.12
CPn0529CP0223ycaH	2			0.75	0.69	0.32	0.49	-0.08	0	0.24	0.07
CPn0509CP0245	2			0.91	0.86	0.59	0.62	0.15	0	0.05	-0.06
CPn0515CP0238ubiE	2			1.56	0.56	1.09	0.97	0	0	0.14	0.06
CPn0522CP0231	2	UP		2.65	1.4	1.45	1.02	0.31	0	0.67	0.38
CPn0523CP0230	2			2.78	1.74	1.39	0.42	-0.12	0	0.24	0.37
CPn0536CP0216dagA1	2			1.16	0.45	0.81	0.53	0.33	0	0.11	-0.13
CPn0579CP0169yacM	2			2.91	1.98	1.56	0.98	0.08	0	-0.2	0.27
CPn0198CP0569oppA4	2			1.9	0.99	0.72	0.62	0.45	0	0.39	0.4
CPn0562CP0188	2			1.71	1.3	0.68	0.52	0.22	0	0.3	0.47
CPn0565CP0185	2			2.42	1.18	0.95	0.82	-0.07	0	1.02	0.74
CPn0572CP0177	2		Yes	2.65	1.43	0.79	0.91	-0.17	0	0.65	0.88
CPn0682CP0065dppD	2	UP		3.07	1.69	1.45	1.1	0.64	0	0.54	0.18
CPn0766CP1106	2			2.65	1.3	1.27	1.01	0.68	0	0.31	0.2
CPn0730CP0016mviN	2	UP		1.5	0.9	0.76	0.91	0.17	0	0.5	0.13
CPn0499CP0255	2	UP		2.47	1.69	1.19	1.22	0.31	0	0.75	1.13
CPn1011	2			1.54	0.73	0.72	0.42	0.46	0	-0.18	0.22
CPn0960CP0900	2	UP		2.53	1.73	1.42	0.96	0.35	0	0.38	0.47
CPn0976CP0880	2	UP		2.97	1.94	2.48	1.44	0.42	0	0.92	0.84
CPn0997CP0858mesJ	2	UP		0.96	0.9	0.91	0.38	-0.14	0	0.5	0.03
CPn1010CP0843	2			1.21	1.07	1.17	0.61	0.18	0	0.15	-0.12
CPn1012CP0841yzeB	2			1.64	0.82	0.72	0.58	-0.12	0	-0.03	-0.19
CPn0944CP0915	2			2.76	2.8	1.85	0.97	0.22	0	0.33	1.06
CPn1018CP0835	2	UP		2.52	1.49	1.56	1.04	0.48	0	0.63	0.67
CPn1027CP0825	2			2.63	1.79	1.31	1.33	0.41	0	0.45	0.29
CPn1035CP0817aroE	2			1.32	-0.01	0.76	0.51	-0.13	0	0.51	0.17
CPn1045CP0807	2			1.12	0.63	0.68	0.49	0.3	0	0.02	-0.13
CPn0958CP0902plsB	2			2.48	1.18	0.89	0.88	0.45	0	-0.01	-0.02
CPn0929CP0937	2	DOWN		3.43	2.42	0.78	0.46	0.11	0	0.31	0.84
CPn0943CP0917	2			2.83	1.82	1.15	0.16	-0.08	0	1.05	0.97
CPn0772CP1100uvrD	2			0.95	0.51	0.76	0.16	-0.08	0	-0.02	-0.09
CPn0831CP1039	2	UP		2.07	1.01	0.82	0.51	-0.2	0	0.37	-0.27
CPn0783CP1089	2	UP		2.01	1.35	1.7	1.08	0.51	0	0.69	0.36
CPn0788CP1084sdhC	2	UP		2.26	1.29	1.01	0.85	0.04	0	0.13	-0.02
CPn0790CP1082sdhB	2	UP		2.1	0.91	1.28	0.52	0.47	0	-0.05	0
CPn0823CP1048yscT	2			2.26	1.72	0.99	0.52	0.15	0	0.36	0.17
CPn0837CP1032nth	2	UP		2.38	2.05	1.31	0.86	0.56	0	0.62	0.25
CPn0928CP0938	2			2.57	1.95	1.46	0.86	0.01	0	0.1	0.13
CPn0838CP1031thdF	2			1.47	1.05	0.83	0.74	0.16	0	0.1	-0.48
CPn0843CP1026	2			2.42	0.83	0.75	0.55	0.3	0	0.67	0.39

CPn0882CP0984	2			1.8	0.51	0.77	0.41	0.16	0	0.62	-0.05
CPn0898CP0968groEL3	2			1.65	0.8	0.79	0.72	-0.18	0	0.46	0.38
CPn0505CP0248	2			1.77	1.03	1.12	0.85	0.05	0	0.32	-0.12
CPn0561CP0189euo	2			2.43	2	1.42	0.61	-0.09	0	0.47	1.44
CPn0492CP0262	2	UP		3.32	2.21	1.74	1.26	0.34	0	0.66	0.35
CPn0486CP0268	2	UP		2.07	1.29	1.33	0.76	0.39	0	0.3	0.58
CPn0313CP0445acpS	2			1.11	0.59	0.79	0.25	0.33	0	0.09	-0.1
CPn0281CP0477dhnA	2	UP		2.96	1.67	1.35	0.16	0.28	0	0.64	-0.02
CPn0284CP0474	2			1.5	0.96	1.05	0.89	0.4	0	-0.09	-0.06
CPn0285CP0473	2			2.06	1.24	1.07	0.55	0.17	0	0.2	0.48
CPn0287CP0471	2			1.12	0.74	0.45	0.57	-0.19	0	-0.1	0.35
CPn0288CP0470	2	UP		1.17	0.57	1.07	0.57	0.23	0	0.62	0.52
CPn0320CP0437ribF	2			1.08	1.28	0.85	0.87	0.07	0	0.43	-0.33
CPn0268CP0492	2	UP		3.17	2.1	1.9	1.08	-0.01	0	0.55	0.64
CPn0328CP0429	2	UP		1.81	0.52	1.07	0.6	0.15	0	0.31	0.34
CPn0336CP0422yojL	2			1.74	0.73	0.75	0.66	0.15	0	0.45	0.21
CPn0353CP0406	2	UP		1.92	0.79	1.47	0.73	0.35	0	0.1	0.17
CPn0354CP0405	2			0.56	0.22	0.64	-0.08	0.05	0	0.28	0.05
CPn0366CP0391	2	UP		2.96	1.67	1.04	0.95	-0.29	0	0.63	0.97
CPn0272CP0487dnaX2	2			1.05	0.78	0.49	0.59	0.13	0	-0.34	0.07
CPn0267CP0493	2			3.04	2.07	1.64	0.89	0.31	0	0.47	0.19
CPN0368_CP0389	2			1.52	0.93	0.42	0.49	0.08	0	0.45	0.4
CPn0224CP0541	2			2.48	1.5	0.48	0.63	0.24	0	0.5	1
CPn0201CP0566oppD	2			1.38	0.25	0.79	0.47	0.32	0	0.02	0.54
CPn0212CP0553	2	UP		3.22	2.14	1.61	0.76	0.55	0	0.58	0.98
CPn0218CP0547	2	UP		2.87	2.18	1.06	1.38	0.1	0	0.7	0.46
CPn0219CP0546tgt	2			0.93	0.68	0.78	0.75	0.01	0	0.13	0.3
CPn0223CP0542	2			2.32	1.32	1.16	1.11	0.03	0	0.3	0.28
CPn0225CP0539	2	UP		2.06	1.3	0.71	0.99	-0.1	0	0.26	0.55
CPn0266CP0495	2	UP		3.79	2.88	1.91	1.59	0.33	0	0.69	0.77
CPn0226CP0538	2			1.73	0.7	0.04	0.68	-0.18	0	0.63	0.26
CPn0240CP0522	2	UP		2.53	1.86	1.78	0.89	0.59	0	0.2	0.72
CPn0241CP0521	2			1.98	0.82	0.78	0.84	0.52	0	0.3	0.51
CPn0242CP0520	2			2.61	1.42	0.42	0.09	0.26	0	1.06	1.3
CPn0261CP0500ydaO	2	UP		1.5	0.75	1.22	0.94	-0.23	0	-0.34	-0.11
CPn0367CP0390	2			1.48	0.6	0.43	0.72	-0.05	0	0.05	0.17
CPn0348CP0412ytgB1	2	UP		1.97	0.93	1.16	0.36	0.27	0	0.72	0.12
CPj0339Cpn0339	2			1.46	0.71	0.62	0.65	0.46	0	0.55	0.66
CPn0452CP0301pmp12	2			1.68	1.37	1.08	0.5	-0.29	0	-0.15	0.23
CPn0431CP0322	2			1.02	0.69	0.74	0.26	0.28	0	0.49	0
CPn0432CP0321	2			1.36	0.82	0.48	0.34	-0.14	0	-0.11	-0.14
CPn0436CP0317	2		Yes	1.81	0.85	0.38	0.57	-0.05	0	0.34	0.33
CPn0439CP0314	2			1.72	1.43	0.96	0.79	0.42	0	-0.66	0.02
CPn0442CP0311	2			2.36	1.22	1.46	0.85	0.6	0	0	0.19
CPn0461CP0291	2			1.26	0.53	1.14	0.42	0.03	0	0.46	0.27
CPn0406CP0349	2		Yes	0.75	0.25	0.55	0.14	0.3	0	-0.06	0.53
CPn0464CP0288	2	UP		3.09	2.41	1.69	0.93	0.21	0	0.53	0.77
CPn0465CP0287	2	UP		2.62	1.94	1.82	0.95	0.21	0	0.41	0.63
CPn0470pmp173	2			1.9	0.93	1.09	0.75	-0.19	0	0.11	0.54
CPn0477CP0277yqeV	2			1.37	1.05	1.07	0.7	0.45	0	0.11	0.01
CPn0481	2			0.66	0.55	0.15	0.02	0.34	0	0.11	-0.08
CPn0429CP0324nqr4	2			1.85	0.93	0.47	0.59	0.14	0	0.15	0.02
CPn0435CP0318	2			0.64	0.43	0.23	-0.09	0.13	0	0.05	-0.13
CPn0380CP0376hemN1	2	UP		2.83	1.78	1.81	1.11	-0.04	0	0.89	0.6
CPn0372CP0385	2			1.62	1.43	0.85	0.37	0.5	0	0.04	0.08
CPn0400CP0355	2			1.81	0.66	0.75	0.67	0.07	0	0.57	0.38

CPn0404CP0351	2	UP		2.24	1.52	1.3	0.87	-0.46	0	0.68	0.79
CPn0737CP0008recC	1			0.85	0.22	-0.11	0.12	0.19	0	0.32	-0.5
CPn1072CP0777	1			1.12	-0.09	0.39	-0.2	0.26	0	0.41	0.12
CPn0475CP0279glgB	1			0.72	0.08	0.3	0.47	-0.17	0	-0.14	0.09
CPn0751CP1121	1			1.07	-0.09	-0.19	0.49	0.76	0	0.64	-0.14
CPn0957CP0903ide	1			0.58	-0.16	0.32	0.27	0.46	0	0.05	-0.01
CPn0980CP0876	1			0.14	-0.26	-0.02	0.04	0.12	0	0.16	-0.24
CPn0480CP0274	1			1.06	0.5	0.27	0.45	0.2	0	0.24	-0.23
CPn0526CP0226kpsF	1			1.02	0.33	0	0.42	-0.17	0	-0.19	-0.23
CPn0654CP0093yciA	1			1.45	0.44	0.37	0.47	-0.24	0	0.28	-0.08
CPn0763CP1109ygfA	1			2.01	0.56	0.6	0.48	0.51	0	0.17	-0.31
CPn0516CP0237	1			1.99	0.72	0.17	0.17	0.15	0	0.12	-0.19
CPn0514CP0239	1			1.12	-0.14	0.27	0.44	0.41	0	0.06	-0.04
CPj0820CPn0820	1			0.73	-0.2	0.22	0.3	0.55	0	-0.04	0.15
CPn0483CP0271	1			0.69	0.04	0.4	0.2	0.58	0	0.37	0.08
CPn0947CP0912pgsA2	1	UP		1.6	0.67	0.68	0.57	0.38	0	-0.05	-0.12
CPn0815CP1056gspD	1		Yes	1.11	0.26	0.25	0.18	0.54	0	0.12	0.12
CPn0764CP1108	1			1.61	0.53	-0.07	0.1	-0.04	0	0.21	-0.26
CPn0765CP1107	1			1.76	0.13	0.18	0.22	0.55	0	0.11	-0.15
CPn0814CP1057	1			1.34	0	0.29	0.53	0.01	0	0.34	0.47
CPn0830CP1041	1	UP		0.48	-0.33	0.28	0.42	0.3	0	-0.07	-0.34
CPn0819CP1052	1			0.69	-0.18	0.15	0.36	0.28	0	0.15	-0.09
CPn0821CP1050	1			0.6	0.25	0.23	0.25	0.38	0	-0.05	-0.47
CPn0662CP0085aspS	1		Yes	1.16	0.64	0.26	0.43	0.39	0	-0.15	-0.2
CPn0817CP1054gspF	1			0.79	-0.12	0.14	0.3	0.57	0	-0.13	-0.22
CPn0784CP1088exbD	1			1.37	0.1	0.29	0.56	-0.32	0	0.25	-0.37
CPn0844CP1025yphC	1			1.04	0.31	0.36	0.29	0.61	0	0	-0.17
CPn0816CP1055gspE	1			1.78	0.52	0.75	0.71	0.23	0	-0.02	0.04
CPn0381CP0375	1			1.17	0.54	0.63	0.77	0.27	0	-0.07	-0.39
CPn0484CP0270aroG	1			0.78	0.31	0.15	0.37	-0.18	0	-0.13	-0.36
CPn0868CP1001zntA	1			2.53	1.12	0.64	0.34	0.17	0	-0.05	-0.18
CPn0878CP0991	1			0.53	0.19	0.27	0.29	0.18	0	-0.09	-0.33
CPn0862CP1007yfhO2	1			2.07	0.34	0.36	0.32	0.06	0	0.08	-0.11
CPn0031CP0745rs21	1			1.19	0.82	0.08	0.13	0.02	0	-0.07	-0.24
CPN03681_CP03891	1	DOWN		1.26	0.69	0.14	0.48	0	0	-0.1	-0.05

(B) persistent infection

NAME	where in acute infection	DAM 24 h	DAM 48h	DAM 72h	Repleted 96 h	120h
CPn1073CP0776	2	0.4	1.1	0.96	2.62	1.62
CPn1072CP0777	1	0.26	0.55	0.77	1.89	0.95
CPn1069CP0781yfgA	10	0.05	0.13	0.36	-0.26	0.28
CPn1067CP0783def		0.13	0.67	0.74	0.13	0.24
CPn1066CP0784		-0.08	0.85	0.9	-0.43	0.02
CPn1065	3	0.1	1.15	1.03	0.72	1.2
CPn1064CP0785		0.1	0.2	0.83	-0.33	-0.05
CPn1063CP0786tpiS	2	0.04	0.87	1.06	0.66	0.31
CPn1060CP0790tktB2	6	-0.06	-0.33	-0.84	-0.63	-0.69
CPn1059CP0791kgsA	4	0.16	-0.24	-0.41	-0.42	-0.62
CPn1058CP0792		0.01	0.77	0.7	-0.51	-0.42
CPn1057CP0793		0	0.1	-1.55	1.26	0.65
CPn1055CP0795		0.14	0.58	0.7	0.69	0.37
CPn1053CP0799	3	-0.05	0.25	0.73	0.8	0.42
CPn1052CP0800	3	0.22	0.93	0.06	0.23	0.33
CPn1050CP0802dapA		-0.03	0.61	0.74	-0.21	-0.15

CPn1049CP0803lysC	7	-0.1	-0.79	-0.68	-0.51	-0.5
CPn1039CP0813aroA		-0.19	0.85	0.54	-0.46	-0.76
CPn1038CP0814aroL		-0.16	0.62	0.92	0.04	0.13
CPn1037CP0815aroC		0.05	0.38	0.67	0.03	0.17
CPn1036CP0816aroB		0.18	-0.02	0.16	-0.18	-0.9
CPn1035CP0817aroE	2	-0.13	0.31	-0.05	2.04	0.31
CPn1034CP0818	10	0.03	0.14	0.49	0.42	0.06
CPn1033CP0819	10	-0.5	-1.28	-0.9	-1.21	-0.07
CPn1032CP0820	9	-0.48	-0.94	-0.43	-0.23	-0.17
CPn1031CP0821arcD	7	0.06	-0.92	-0.18	-0.8	0.16
CPn1030CP0822		-0.07	0.49	0.76	0.72	0.12
CPn1029CP0823	8	-0.28	0.45	0.59	-0.46	-0.67
CPn1027CP0825	2	0.33	0.7	-0.08	1.53	0.78
CPn1025CP0827pgi	12	0.1	-0.15	-1.05	0.25	0.69
CPn1024CP0828xerD		0.27	0.58	0.56	0.34	0.14
CPn1023CP0829	12	0.08	-0.9	-1.55	0.56	0.83
CPn1018CP0835	2	0.45	2.12	1.63	0.57	2.14
CPn1017CP0836lytB	6	0.36	-0.22	0.02	-0.5	-0.37
CPn1015CP0838	5	0.22	0.64	1.16	-0.25	-0.55
CPn1013CP0840fumC	9	-0.58	-0.71	-0.38	-0.59	-0.54
CPn1011	2	0.08	-0.18	-0.82	-0.54	0.6
CPn1007CP0846		-0.19	-0.02	0.14	0.5	0.18
CPn1006CP0847	12	-0.39	-1.5	-1.03	-0.9	1.28
CPn1005CP0848	11	-0.36	-1.52	-1.69	-0.29	0.77
CPn1004CP0849	11	-0.3	-1.84	-1.52	-0.53	0.5
CPn1001CP0852yfhC	8	0.17	0.83	0.96	-0.04	0.54
CPn0999CP0855pnp		0.04	0.45	0.74	1.31	0.58
CPn0998CP0857ftsH	10	-0.14	-0.78	-0.82	-0.09	0.1
CPn0997CP0858mesJ	2	-0.02	1.07	0.98	1.09	0.25
CPn0995CP0860		-0.1	-0.06	-0.2	-0.56	-0.94
CPn0994CP0861	11	0.14	-1.09	-1.56	-0.22	-0.57
CPn0990CP0865infC	6	-0.01	1.26	1.02	-0.5	-0.33
CPn0988CP0867murB		0.02	0.05	-0.05	-0.3	-0.13
CPn0986CP0869yggH	3	0.24	0.93	0.84	0.05	0.01
CPn0985CP0871nrdB	7	-0.24	-0.96	-1	-0.91	-0.43
CPn0984CP0872nrdA	7	0.03	-1.2	-1.04	-0.81	-0.08
CPn0981CP0875		0.31	0.85	0.87	-0.3	0.81
CPn0980CP0876	1	0.06	0.3	0.59	0.7	0.04
CPn0979CP0877htrA	2	-0.25	1.25	1.16	-0.13	0.69
CPn0976CP0880	2	0.47	1.53	1.04	1.95	2.66
CPn0971CP0888yccA2	10	0.1	0.65	0.65	0.41	0.67
CPn09691yccA1	10	-0.18	0.08	0.94	0.44	0.37
CPn0966CP0894pcnB2	5	0.25	-0.59	-0.63	0.03	-0.49
CPn0961CP0899rl32	4	-0.04	0.79	1.18	0.33	0.12
CPn0960CP0900	2	0.67	2.07	1.77	-0.29	1.92
CPn0956CP0904	10	-0.6	-1.03	-0.81	-0.71	-0.62
CPn0948CP0911glgA		0.46	1.15	0.62	-0.82	0.95
CPn0947CP0912pgsA2	1	0.22	1.18	0.77	1.8	0.53
CPn0938CP0923		-0.11	0.46	1.17	-0.46	-0.82
CPn0936CP0925rl36	9	0.18	0.39	0.27	-0.93	0.62
CPn0935CP0926rl34		0.43	0.05	-0.02	0.26	0.27
CPn0934CP0927rnpA	3	0.57	-0.64	-0.71	-0.2	0.02
CPn0930CP0935	12	0.32	-0.46	-1	0.57	0.42
CPn0929CP0937	2	-0.12	-0.43	-1.35	0.58	0.45
CPn0927CP0939	3	-0.28	0.35	0.4	0.9	0.37
CPn0921CP0945	8	-0.32	-0.5	-0.45	-0.81	-0.53

CPn0913CP0953	9	0.52	-0.38	-0.69	-1.17	-0.28
CPn0912CP0954	6	0.6	0.07	-0.3	-1.2	-0.51
CPn0909CP0957rsbV2	8	-0.06	0.06	0.85	-0.46	-0.21
CPn0902CP0964nlpD	9	0.24	0.99	1.54	0.83	-0.03
CPn0897CP0969		-0.19	-0.26	0.02	0.15	0.19
CPn0895CP0971efp2		-0.39	0.29	0.9	-0.56	-0.27
CPn0894CP0972amn	9	-0.15	-1.26	-0.82	-0.13	-0.61
CPn0891CP0975mfd	6	-0.29	0.6	0.74	0.57	-0.14
CPn0888CP0978hemG		0.15	0.3	0.04	-0.9	-0.99
CPn0887CP0979		-0.18	0.69	0.72	0.94	0.52
CPn0884CP0982	8	-0.17	-1.03	0.02	-1.57	-0.32
CPn0882CP0984	2	-0.21	0.1	0.59	-0.09	0.23
CPn0879CP0990yycJ		-0.12	-0.9	-0.72	-0.59	-0.78
CPn0873CP0996ribE	8	-0.07	0.15	0.14	-0.88	0.2
CPn0871CP0998ribD	3	0.12	0.57	0.86	0.13	0.2
CPn0870CP0999serS	7	-0.06	-0.61	-0.87	-1.12	-0.29
CPn0869CP1000		0.18	-0.45	-0.29	-1.12	-0.56
CPn0867CP1002rodA	3	0.42	1.25	1.09	1.35	0.48
CPn0866CP1003birA		0.01	0.64	0.8	-0.07	-0.35
CPn0862CP1007yfhO2	1	0.25	0.61	0.78	2.53	0.28
CPn0860CP1009fliF	10	-0.7	-1.8	-0.95	-1.26	-0.23
CPn0859CP1010		0.31	-0.03	-0.19	1.2	0.28
CPn0857CP1012	10	-0.19	-1.74	-0.81	-1.48	-0.37
CPn0853CP1016	11	-0.08	-2.01	-1.81	-0.56	0.26
CPn0852CP1017	11	-0.29	-2.49	-2.29	-0.66	0.18
CPn0849CP1020	8	-0.17	-1	-0.91	-1.35	-0.61
CPn0848CP1021tigA	4	-0.12	-0.37	-0.73	-1.02	-0.23
CPn0844CP1025yphC	1	0.34	0.65	0.31	-0.85	-0.66
CPn0843CP1026	2	0.46	0.7	0.42	1.32	0.98
CPn0842		-0.2	0.13	-0.1	1.69	0.73
CPn0841CP1028secA2		0.13	-1	-0.87	-0.83	-0.35
CPn0837CP1032nth	2	0.8	2.17	1.62	3.58	2.09
CPn0831CP1039	2	0.32	0.76	1	1.78	0.49
CPn0830CP1041	1	0.08	0.89	1.36	0.77	-0.11
CPn0829CP1042	4	0.12	0.82	0.91	0.52	0.33
CPn0826CP1045yscL	7	0.16	-1.03	-0.76	-1.12	-0.56
CPn0824CP1047yscS	10	-0.21	-0.61	-0.76	-1.3	-0.5
CPn0822CP1049	10	-0.26	-0.56	-0.31	0.24	-0.04
CPn0818CP1053		0.14	-0.51	-0.67	-0.57	-0.44
CPn0817CP1054gspF	1	0.3	-0.44	-0.72	-0.06	-0.95
CPn0813CP1058pepP	2	0.25	0.32	0.45	-0.35	-0.17
CPn0811CP1060lcrH1	11	-1.03	-3.11	-1.32	-0.44	1.41
CPn0810CP1061	11	-0.63	-2.31	-1.84	-0.49	1.21
CPn0809CP1062	11	-0.3	-1.89	-0.64	0.07	1.51
CPn0808CP1063	10	-0.32	-2.25	-1.94	-0.06	1.38
CPn0807CP1064		0.32	1.18	1.09	0.06	0.13
CPn0806CP1065thrS	7	-0.05	0.47	0.95	-0.79	-0.44
CPn0801CP1070	4	-0.45	-0.76	-0.84	-0.62	-0.24
CPn0798CP1073	9	-0.22	0.9	1	0.14	-0.47
CPn0794CP1077	11	-0.39	-1.61	-0.91	-0.87	-0.18
CPn0791CP1081	11	-0.18	-1.01	-1.19	-0.45	0.08
CPn0790CP1082sdhB	2	-0.04	1	0.35	1.57	0.16
CPn0788CP1084sdhC	2	0.53	1.37	0.8	3.15	0.86
CPn0787CP1085yabD	3	0.15	1.3	0.94	0.48	0.04
CPn0783CP1089	2	0.35	1.68	1.29	0.94	1.05
CPn0780CP1092amiB		-0.39	-0.67	-0.32	-0.78	-0.92

CPn0779CP1093		-0.19	-0.59	0.29	0.36	-0.2
CPn0778CP1094ahpC	7	-0.47	0.46	0.88	-0.5	-0.38
CPn0777CP1095groEL2	10	0.1	-1.11	-1.05	-0.21	-0.63
CPn0773CP1099ung		0.3	0.33	0.4	0.54	-0.48
CPn0769CP1103topA	10	0.09	-0.94	-0.7	-1.45	-0.21
CPn0767CP1105		-0.35	0.7	0.8	0.34	-0.3
CPn0755CP1117		0.1	-0.41	-0.13	1.57	0.06
CPn0753CP1119	6	0.02	1.08	0.91	-0.7	-0.07
CPn0750CP1122tctD	9	-0.72	-1.7	-1.36	-0.48	-0.26
CPn0742CP0003	10	-0.37	-0.97	-0.64	0.37	0.97
CPn0740tyrB	6	0.02	-0.02	-0.4	-0.58	-0.85
CPn0738CP0007	6	-0.32	-0.43	-0.56	-0.13	-0.68
CPn0734yceA	5	0.11	0.11	-0.06	-0.38	-0.46
CPn0732nfo		0.48	0.53	0.73	1.06	1.15
CPn0731CP0015	10	-0.18	-0.42	-0.42	0.61	0.44
CPn0730CP0016mviN	2	0.62	1.32	0.53	1.81	0.18
CPn0728CP0018	11	0.06	-1.88	-2.34	0.24	1.37
CPn0727CP0019	11	-0.03	-2.09	-2.41	-0.46	0.56
CPn0726	12	0.05	-0.77	-0.75	0.2	0.7
CPn0725	11	-0.47	-1.52	-1.59	0.14	0.39
CPn0724CP0022	12	0.02	-0.64	-1.06	0.23	0.21
CPn0723yhbG	3	0.18	0.69	1.07	2.05	1.17
CPn0720CP0026	11	-0.66	-0.48	-1.32	-0.04	1.19
CPn0718CP0028		0.1	-0.74	-0.53	0.11	-0.1
CPn0716CP0030	6	-0.36	-0.85	-0.59	-0.52	-0.52
CPn0712CP0034	8	-0.01	-1.35	-1.01	-0.95	-0.55
CPn0711CP0035	7	-0.41	-1.39	-0.78	-1.05	-0.33
CPn0708CP0038	8	-0.11	-1.35	-0.87	-0.79	-0.2
CPn0707CP0039yscN	7	-0.27	-1.16	-0.96	-1.2	-0.28
CPn0706CP0040		-0.47	-0.69	-1.1	-0.12	-0.81
CPn0705CP0041	7	-0.45	-1.43	-1.17	-0.82	-0.78
CPn0704fliN	8	-0.26	-0.82	-0.8	-0.83	-0.8
CPn0702CP0044yscC	7	-0.5	-1.61	-0.95	-1.03	-0.37
CPn0701CP0045karG	10	0.34	-1.06	-0.81	-1.07	0.11
CPn0700CP0046	11	-0.71	-1.12	-0.73	-0.59	0
CPn0697CP0049tsf		0.42	0.04	-0.09	-1.28	0.03
CPn0696rs2		0.25	0.32	0.24	-0.75	0.03
CPn0693	11	-0.25	-1.24	-1.19	-0.33	0.05
CPn0688CP0058	5	0.07	-0.82	-0.7	-0.58	-0.87
CPn0685CP0061		1.24	-0.34	0.47	-0.38	0.46
CPn0682CP0065dppD	2	0.51	1.38	1.26	2.78	1.31
CPn0679CP0068	1	0.36	1.52	1.23	2.43	1.18
CPn0678	12	0.01	-0.72	-0.72	1.12	2.46
CPn0677CP0070	10	-0.28	-2.2	-1.83	0.13	1.63
CPn0676CP0071	12	-0.01	-2.3	-2.46	-0.05	1.56
CPn0675CP0072		-0.14	-0.88	-1.85	0.44	0.08
CPn0670rsbW	7	-0.25	-1.26	-0.91	-1.12	0
CPn0669CP0078	10	-0.45	-1.35	-0.84	-0.92	0.2
CPn0664		0.08	1.02	1.02	0.65	0.89
CPn0660spoU2		-0.13	-0.4	-0.14	-0.96	-0.61
CPn0658	5	-0.18	0.28	0.78	0.02	-0.18
CPn0657CP0090		-0.11	-0.29	-0.3	-0.72	-0.02
CPn0655CP0092dnaQ2	4	-0.12	-0.47	-0.94	-1.17	-0.2
CPn0654CP0093yciA	1	0.18	0.78	0.8	0.9	0
CPn0651CP0096fabZ		0.11	-0.51	-0.68	-0.9	-0.12
CPn0650CP0097lpxA	6	-0.11	-0.21	-0.59	-0.84	-0.36

CPn0649CP0098fmt	4	0.04	-1.02	-0.92	-0.72	0.31
CPn0635CP0112rl5	5	0.14	-0.21	-0.72	-1.38	-0.48
CPn0634CP0113rs8	5	-0.08	-0.21	-0.85	-1.44	-0.63
CPn0633CP0114rl6	5	-0.11	-0.6	-0.91	-1.26	-0.29
CPn0632CP0115rl18	5	0.21	-0.54	-0.87	-1.31	-0.49
CPn0631CP0116rs5	6	0.2	-0.6	-0.98	-1.38	-0.27
CPn0630CP0117rl15	5	-0.06	-0.47	-0.78	-1.29	-0.45
CPn0629CP0118secY	4	0.34	-0.49	-0.85	-1.53	-0.15
CPn0626CP0121rpoA	5	-0.2	-0.34	-0.64	-1.53	-0.73
CPn0620CP0127ruvA	2	0.16	1.05	0.88	2.13	0.98
CPn0618CP0129lplA2	2	-0.17	1.1	0.95	2.7	1.44
CPn0613CP0134sohB	6	0.11	0.29	0.71	-0.01	-0.3
CPn0611CP0136yacE	4	-0.15	-0.62	-0.74	-0.27	-0.37
CPn0610CP0137rho		-0.5	-0.32	-0.9	-0.39	-0.01
CPn0607CP0140glgC	12	0.06	-1.14	-1.21	0.77	-0.25
CPn0596CP0152ada	2	0.45	0.7	0.49	1.82	0.82
CPn0589CP0159	3	0.02	-0.7	-1.03	-0.76	-0.08
CPn0588CP0160	2	0.47	1.1	-0.21	2.96	1.67
CPn0587CP0161yvyD	11	-0.28	-0.48	-0.72	0.12	0.54
CPn0586CP0162atoC	12	0.06	0.16	0.2	2.11	0.68
CPn0584CP0164atoS	11	-0.36	-1.46	-0.96	-0.97	-0.16
CPn0583CP0165		-0.22	-0.24	-0.69	-0.51	0.3
CPn0580CP0168truA	2	0.14	0.99	0.8	2.03	0.72
CPn0579CP0169yacM	2	0.41	0.35	0.12	1.4	1.31
CPn0578CP0170yaeI	4	0.07	-0.04	-0.24	0.05	-0.24
CPn0577CP0171	9	0.3	0.06	0.19	-1.32	-0.11
CPn0575aprfB	5	0.16	-0.09	0.27	-0.23	-0.32
CPn0572CP0177	1	0.3	0.47	-0.97	2.34	1.35
CPn0568CP0181		0.07	-0.65	-0.64	0.78	-0.57
CPn0567CP0182cdsA		-0.03	-0.55	-0.66	-1.1	-0.77
CPn0565CP0185	2	0.38	0.38	-0.03	0.21	0.61
CPn0559CP0191	12	0.28	0.01	-0.89	1.79	2.18
CPn0558CP0193	11	-1.36	-1.18	0.01	-0.29	1.78
CPn0557CP0195omcB	10	-1.85	-2.27	-0.44	-0.51	1.18
CPn0555CP0197tsp		-0.13	-1.24	-1.09	0.33	0.17
CPn0554CP0198	3	0.18	1.29	1.03	1.61	0.57
CPn0550fusA	4	-0.27	-0.3	-0.61	-1.32	-0.56
Cpn0548_CP0204	5	-0.1	-0.09	-0.64	-1.04	-1.27
CPn0545CP0207rl27		-0.2	0.61	0.97	-0.1	0.67
CPn0542CP0210	4	0.28	0.56	0.06	0.49	-0.2
CPn0539CP0213pmp19	5	0.07	0.68	0.85	-0.08	-0.59
CPn0538CP0214	11	-1.11	-1.72	-1.8	0.25	1.32
CPn0537CP0215	12	-0.47	-1.66	-1.63	1.32	1.66
CPn0535CP0217lspA	8	0.4	-0.24	-0.79	-1.47	-0.5
CPn0529CP0223ycaH	2	0.03	0.7	0.78	0.31	-0.04
CPn0528CP0224gltT		0.17	0.41	0.81	-0.55	0.09
CPn0524CP0229	12	-0.37	-1.11	-1.88	-0.31	0.96
CPn0522CP0231	2	0.21	1.01	1.04	2.48	1.01
CPn0517CP0236		0.09	0.75	-0.13	2.15	0.55
CPn0515CP0238ubiE	2	0.35	0.85	0.6	1.28	-0.18
CPn0514CP0239	1	0.3	0.26	-0.05	0.07	-0.15
CPn0513CP0240		0.2	0.38	0.11	0.45	-0.41
CPn0504CP0249vacB	5	-0.34	-0.33	-0.56	-1.24	-0.64
CPn0503CP0251dnaK		-0.48	-0.87	-0.25	-0.5	0.23
CPn0502CP0252grpE		0.24	-0.2	-0.11	-0.65	0.03
CPn0499CP0255	2	0.36	1.26	-0.05	3.11	2.06

CPn0496CP0258		0.27	0.28	0.18	-0.27	-0.2
CPn0495CP0259aspC	8	0.58	0.52	0.4	-0.54	0.08
CPn0494CP0260	12	0.06	-1.12	-1.23	0.85	1.51
CPn0493CP0261	12	0.17	0.22	-0.82	1.61	1.41
CPn0492CP0262	2	0.49	1.66	1.23	2.77	1.83
CPn0487CP0267	6	0.2	-0.2	-0.85	0.13	-0.85
CPn0486CP0268	2	0.39	1.15	0.78	2.63	1.44
CPn0473CP0281	12	0.29	1.18	0.15	1.86	2.24
CPn0472CP0282	11	-0.23	-1.16	-1.92	-0.35	1.16
CPn0470pmp173	2	0.08	0.04	0.03	1.11	0.63
CPn0467CP0285pmp16	10	0.07	-1.11	-1.03	-1.05	-0.05
CPn0466CP0286pmp15	11	-0.09	-0.37	-0.64	0.46	0.54
CPn0465CP0287	2	0.41	1.54	1.4	1.63	1.37
CPn0464CP0288	2	0.3	1.58	1.09	2.61	0.94
CPn0463CP0289	3	0.01	0.59	0.52	2.41	0.59
CPn0461CP0291	2	0.13	0.76	0.38	0.79	0.76
CPn0459CP0293		-0.15	-0.01	-0.34	1.32	1.2
CPn0454CP0298pmp14		0.12	-0.25	0.02	1.46	0.73
CPn0453CP0299pmp13	9	-0.56	-1.16	-0.84	-1.74	-0.33
CPn0448CP0305yxjG2	7	-0.44	0.02	0.14	-0.39	-0.74
CPn0446CP0307pmp8	9	-0.37	-1.77	-1.53	-0.8	-0.09
CPn0445CP0308pmp7	10	-0.26	-0.96	-0.73	-0.23	0.17
CPn0444CP0309pmp6	9	-0.34	-1.05	-0.9	-0.39	-0.27
CPn0443CP0310		0.19	-0.16	-0.76	0.82	1.66
CPn0442CP0311	2	0.49	0.55	0.14	0.83	0.39
CPn0440CP0313	5	-0.16	0.35	-0.77	-0.68	-0.53
CPn0439CP0314	2	0.13	0.23	-0.25	-0.43	0.27
CPn0434CP0319	8	-0.18	-0.32	-0.25	-0.91	0.46
CPn0433CP0320gcsH	10	0.08	-0.34	-0.11	-0.96	-0.05
CPn0429CP0324nqr4	2	0.25	0.42	0.17	0.9	0.32
CPn0419CP0335pbp3	11	-0.01	-0.81	-0.56	-0.63	-0.48
CPn0418CP0336murE	3	0.23	1.26	1.08	-0.5	2.13
CPn0416CP0338himD	10	-0.85	-1.11	0.46	-0.44	0.11
CPn0415CP0339	8	-0.06	-0.52	-1.09	-1.24	-0.95
CPn0408CP0346		0.15	0.45	0.66	0.81	0.57
CPn0406CP0349	1	0.04	0.58	0.48	1.02	0.44
CPn0404CP0351	2	0.73	2.13	1.61	3.44	2.49
CPn0403yceC	3	0.1	1.11	1.31	1.97	1.16
CPn0402CP0353mutY	7	-0.14	-0.2	0.02	0.06	0.05
CPn0395CP0360		-0.62	-0.34	-0.58	-0.42	-0.38
CPn0391CP0364		-0.75	-0.05	0.17	0.28	0.09
CPn0390CP0365ruvB	8	-0.15	-0.45	-0.22	-0.34	0.19
CPn0389CP0366	8	-0.35	-0.2	0.08	-0.57	-0.52
CPn0386CP0369ssb		-0.15	-0.64	-0.5	-0.96	-0.34
CPn0385CP0370pepA		0.03	-0.29	-0.65	-0.83	-0.17
CPn0384CP0371hctB	11	-2.46	-2.97	-0.29	-0.81	1.14
CPn0380CP0376hemN1	2	0.05	1.18	1.01	2.57	1.56
CPn0376CP0380		-0.51	0.11	0	0.81	0.77
CPn0375CP0381		-0.16	0.35	0.32	-0.69	0.27
CPn0370CP0387		0.19	-0.11	-0.39	0.67	0.61
CPn0369CP0388	12	-0.02	-1.04	-1.12	0.53	1.02
CPN03681_CP03891	1	-0.12	-0.82	-0.96	0.1	0.11
CPn0366CP0391	2	0.36	1.24	0.13	1.05	1.22
CPn0365CP0392	12	0.07	-0.69	-0.79	0.51	0.68
CPn0363CP0394flhA		-0.02	-0.83	-0.66	-0.63	-1.02
CPn0362CP0395rpsD	11	-0.47	-1.38	-1.25	0.03	0.99

CPn0358CP0400	3	0.03	0.06	0.99	-0.25	-0.39
CPn0357CP0401		-0.13	0.72	1.33	-0.04	-0.58
CPn0355CP0404	10	-0.37	0.26	0.74	-0.74	-0.02
CPn0353CP0406	2	0.14	1.75	1.43	0.41	2.14
CPn0348CP0412ytgB1	2	0.28	0.91	0.95	-0.06	0.55
CPn0340CP0417	12	0.05	-0.93	-1.33	0.44	0.87
CPn0337CP0421smpB	9	-0.08	0.34	0.93	0.27	0.4
CPn0335folD		-0.16	0.56	0.24	-0.59	-0.38
CPn0334CP0424		0.17	0.63	0.43	0.36	0.01
CPn0332	12	0.08	-0.59	-2.06	1.31	2.8
CPn0331CP0426		0.26	0.63	-1.27	1.66	1.9
CPn0330CP0427	12	0.15	-1.74	-2	0.04	1
CPn0328CP0429	2	0.48	1.4	1.11	1.18	1.29
CPn0327CP0430rl28	5	0.11	0.27	0.54	-0.61	0.48
CPn0326CP0431malQ		-0.08	-0.42	0.06	0.68	-0.04
CPn0325CP0432sycE	10	-0.39	-1.59	-1.4	-1.07	-0.73
CPn0324CP0433lcrE	10	-0.35	-1.15	-0.8	-0.48	-0.47
CPn0318CP0439rbfA	5	-0.04	-0.21	-0.88	-1.27	-0.89
CPn0317CP0440infB	5	0.01	-0.37	-0.86	-0.96	-0.92
CPn0310CP044860IM		-0.23	0.48	0.75	-0.27	-0.32
CPn0309CP0449dnaA1	7	-0.08	-0.23	-0.46	-4.83	-0.6
CPn0308CP0450		0.34	0.61	0.21	0.91	0.67
CPn0307CP0451glgP	10	-1.11	-1.2	-0.98	0.21	-0.05
CPn0303CP0455		-0.11	1	1.1	-0.1	0.83
CPn0293CP0465		0.03	0.25	0.7	-0.29	-0.21
CPn0292CP0466incC	3	0.37	0	-0.59	-1.18	-0.25
CPn0291CP0467incB		0.01	-0.19	-1.18	-1.5	-0.54
CPn0290CP0468	4	0.42	0.84	0.74	-0.3	-0.03
CPn0288CP0470	2	0.49	0.9	0.98	1.82	0.99
CPn0285CP0473	2	0.15	0.1	0.19	-0.56	0.08
CPn0283	5	0.21	-0.03	0.11	0.5	-0.16
CPn0282CP0476xasA	8	-0.56	-0.35	-0.46	-1.05	-0.49
CPn0281CP0477dhnA	2	0.42	0.85	0.63	2.21	0.05
CPn0280CP0478dppF1		0.29	1.12	1.04	-0.27	-0.02
CPn0279CP0479	5	0.47	1.53	1.23	-0.48	-0.13
CPn0277CP0481		0.12	1.16	0.98	0.51	0.74
CPn0271CP0488		-0.31	0.51	0.27	-0.83	-0.13
CPn0268CP0492	2	0.01	1.49	1.24	1.99	1.61
CPn0267CP0493	2	-0.35	0.05	-0.02	0.82	-0.34
CPn0266CP0495	2	0.09	1.49	1.08	2.12	1.99
CPn0264CP0497ubiD	3	0.13	1.26	1.02	0.88	0.37
CPn0263CP0498yqfU	7	0.13	1.01	0.77	-0.55	0.2
CPn0261CP0500ydaO	2	0.06	1.62	1.16	0.17	1.02
CPn0260CP0501secA1	8	-0.31	0.91	0.78	-0.03	0.01
CPn0257CP0504		0.29	0.19	0.25	0.96	-0.16
CPn0256CP0505	11	-0.41	0.58	0.62	-0.25	0.48
CPn0255CP0506		0.12	1.01	0.38	1.17	0.84
CPn0254CP0507	12	-0.01	-0.09	-0.19	0.77	0.04
CPn0250CP0511rl33	8	0.13	0.46	0.57	-0.44	1.19
CPn0246CP0516rs9		-0.05	0.42	0.49	-0.08	0
CPn0245CP0517ydhO		0.1	0.34	-0.12	-0.32	-0.18
CPn0244CP0518adk		0.42	0.86	0.64	-0.79	-0.01
CPn0241CP0521	2	0.21	0.58	-0.22	2.38	0.82
CPn0240CP0522	2	0.71	1.69	1.18	2.51	1.79
CPn0237CP0525yggF	4	-0.33	-0.67	-0.69	-0.67	-0.2
CPn0236CP0526pyrG	4	0.07	-0.37	-0.71	-0.66	-0.25

CPn0235CP0527kdsB	4	-0.12	0.19	0.16	-0.09	-0.18
CPn0234CP0529	12	0.08	-0.04	-1.35	1.7	1.23
CPn0233CP0531		-0.07	-0.36	-1.82	0.72	0.13
CPn0231CP0533tauB	9	0.11	1.28	0.69	0.03	-0.07
CPn0229CP0535	10	-0.19	-0.71	-0.56	-0.9	0.07
CPn0228CP0536dsbG	10	-0.18	-2.07	-1.71	-1.72	-0.07
CPn0227CP0537dsbB	10	-0.43	-0.92	-1.53	-1.26	-0.1
CPn0225CP0539	2	0.13	0.93	0.43	0.32	0.32
CPn0223CP0542	2	0.32	0.81	0.07	1.95	0.6
CPn0220CP0545		-0.03	-0.14	-0.91	0.52	1.33
CPn0219CP0546tgt	2	0.11	0.41	0.21	0.13	0.12
CPn0218CP0547	2	0.43	1.6	1.16	3.8	1.49
CPn0213CP0552		0.57	-0.6	-1.21	-0.51	-0.38
CPn0212CP0553	2	0.39	2.04	1.55	3.48	1.98
CPn0211CP0554	3	0.33	1.27	0.92	1.61	0.24
CPn0209CP0557	12	0.03	0.17	0	0.43	0.22
CPn0204CP0563	11	-0.18	-0.58	-0.86	0.47	-0.6
CPn0203CP0564		-0.11	0.8	-0.58	0.25	-0.6
CPn0201CP0566oppD	2	-0.05	0.81	0.25	0.63	0.54
CPn0198CP0569oppA4	2	-0.08	0.21	0.04	0.5	0.47
CPn0197CP0570oppA3	2	-0.12	0.24	-0.33	0.03	0.07
CPn0196CP0571oppA2	2	-0.29	0.12	-0.48	0.32	-0.82
CPn0195CP0572oppA1	10	-0.2	-0.9	-0.89	-0.93	-0.2
CPn0190CP0577	12	0.21	1.01	-0.42	0.62	0.81
CPn0189CP0578	12	-0.26	-1.22	-1.36	-0.62	0.22
CPn0188CP0579	11	-0.28	-1.04	-1.66	-0.48	0
CPn0180CP0588		0.03	-1.12	-1.24	0.24	0.35
CPn0176CP0593		0.12	1.18	0.76	0.69	0.61
CPn0175CP0594		-0.19	0.34	-0.16	-0.22	-0.28
CPn0170CP0600		0.43	0.92	0.72	1.06	1
CPn0169CP0602		-0.05	-0.35	-0.65	-0.46	-0.23
CPn0168CP0603	2	-0.09	1.13	1	2.23	1.39
CPn0165CP0606		-0.28	-0.47	-0.77	-0.06	-0.18
CPn0163CP0608	2	-0.01	0.94	0.18	0.88	0.33
CPn0161CP0610	10	-0.18	-0.4	-0.77	-0.56	-0.37
CPn0159CP0612		0.18	1.12	0.75	0.63	1.38
CPn0158CP0613		0.13	0.67	0.66	0.59	0.65
CPn0157CP0614		0.34	0.51	0.46	0.85	-0.14
CPn0154CP0617gseA	8	-0.62	-0.02	-0.32	-0.64	-0.44
CPn0152CP0620	2	0.16	0.23	-0.19	-0.76	-0.21
CPn0151CP0622mhpA		0	0.24	-0.37	-0.15	-0.34
CPn0150CP0623	3	0.3	0.23	0.52	0.5	0.52
CPn0149CP0624dnlJ	2	0.49	0.86	0.8	3.49	1.52
CPn0144CP0629clpB	8	0.13	-0.1	-0.08	-0.58	-0.06
CPn0143CP0630yxjG1	11	0.37	0.98	0.71	0.41	0.25
CPn0142	12	0.15	-0.6	-1.34	0.4	-0.09
CPn0140CP0632yqdE	10	0.37	-1.47	-0.53	-0.59	0.01
CPn0139CP0633yqgE	10	-0.07	-1.52	-1.34	-0.78	-0.36
CPn0138CP0634hemL	10	-0.09	-0.17	-0.63	-0.24	-0.12
CPn0137CP0635ybgI		0.29	0.6	0.23	0.93	-0.14
CPn0132CP0640	9	0.14	0.62	0.19	0.57	-0.46
CPn0131CP0641		0.22	1.52	1.14	0.97	-0.43
CPn0129CP0642	12	0.17	-0.27	-0.79	0.25	0.1
CPn0127CP0645ytfF	11	-0.12	-0.37	-0.73	-0.16	0.11
CPn0123CP0650recD1		0.18	0.83	0.44	0.31	-0.02
CPn0122CP0651metG	3	-0.04	-0.35	-0.67	-1.01	-0.55

CPn0116CP0657rs16	5	0.25	-0.53	-0.81	-1.38	-0.48
CPn0115CP0658ffh	4	-0.1	-0.21	-0.7	-1.48	-0.53
CPn0113CP0660pfrA	1	-0.1	0.69	0.44	-0.3	-0.11
CPn0112CP0661rl31	9	0.21	0.99	0.68	-0.31	0.13
CPn0108CP0666		0.65	1.17	0.19	2.04	1.04
CPn0105CP0669	10	-0.07	-0.63	-0.41	-0.44	0.15
CPn0102CP0672cydA	2	0.53	1.33	1.06	2.35	1.11
CPn0098htrB	2	0.01	0.71	-0.22	2.07	0.93
CPn0096CP0678uvrA	11	-0.17	-0.24	-0.74	-1.11	-0.37
CPn0092CP0682atpK	5	0.1	0.44	0.74	-0.09	0.1
Cpn0090_CP0684	4	0	-0.07	-0.84	-0.54	-0.86
CPn0086_CP0688	2	0.26	1.52	1.11	2.6	0.5
CPn0085CP0690	11	-0.1	-0.31	-0.17	-0.11	-0.49
CPn0084CP0691		-0.37	-0.71	-0.93	-0.38	-0.77
CPn0081CP0694rpoB	5	-0.09	-0.89	-1.3	-1.01	-0.65
CPn0072CP0703		-0.35	-0.26	-0.55	-0.2	-0.63
CPn0070		-0.26	-0.37	-0.76	0.14	-0.54
CPn0067CP0707	8	0.05	0.23	0.15	0.03	-0.7
CPn0066CP0708	2	0.33	1.89	1.49	1.94	1.72
CPn0065CP0709	12	-0.36	-1.6	-2.1	0.46	1.44
CPn0063CP0711		0.06	-0.75	-0.82	1.25	-0.04
Cpn0061_CP0714	8	-0.1	0.65	0.16	-0.19	-0.46
CPn0059CP0716dut	8	-0.33	0.14	0.06	-0.31	-0.15
CPn0055CP0720	4	0.09	0.3	-0.01	-0.69	-0.28
CPn0054CP0721rnc	2	-0.07	0.37	0.29	0.07	-0.08
CPn0053CP0722sms		0.24	0.58	0.77	0.2	0.25
CPn0047	9	0	0.57	0.89	-0.17	0.44
CPn0046CP0728	2	0.28	1.13	1.04	1.66	0.74
CPn0044CP0729	8	-0.37	-0.19	0.22	0.27	-0.67
CPn0043CP0730	3	-0.12	1	0.72	2.15	0.58
CPn0041CP0733	2	-0.34	0.6	0.78	0.33	0.34
CPn0039CP0736ybaB		0	-0.24	-0.31	-0.75	-0.24
CPn0038CP0737ptsI	2	0.56	1.71	1.99	3.52	2.38
CPn0035CP0741	2	0.02	1.5	1.3	3.01	1.78
CPn0034CP0742	2	0.19	0.93	1.01	1.31	0.61
CPn0028CP0748	2	0	1.33	1.4	1.72	1.32
CPn0026CP0750	3	-0.06	0.29	0.16	1.25	0.15
CPn0025CP0751atsA	3	0.05	1.28	0.37	0.43	0.18
CPn0024CP0752xerC	2	0.28	1.3	1.01	1.58	1.42
CPn0020CP0756	2	0.28	0.94	0.67	1.64	0.32
Cpn0018_pmp51	10	-0.31	-1.05	-0.74	-1.14	-0.34
Cpn0017_pmp42	11	0.25	-0.85	-0.98	0.32	-0.14
Cpn0016_pmp41	11	-0.71	-1.4	-1.09	-0.96	-0.44
Cpn0015_pmp32	10	-0.38	-0.69	-0.67	-1.06	-0.45
Cpn0014_pmp31	10	-0.75	-1.26	-0.67	-1.56	0.09
CPn0009CP0765	2	0.15	1.21	0.85	0.93	0.69
CPn0008CP0766	2	-0.11	0.85	0.77	1.83	0.52
CPn0006	2	-0.2	1.08	1.19	2.07	1.54
CPn0004CP0771gatB	6	0.14	1.09	1.11	-0.62	-0.27
CPn0003CP0772gatA	3	0.22	0.65	0.66	0.28	-0.13
CPn0002CP0773gatC		0.18	0.52	0.84	-1.04	0.13
CPj05762CPn0576	3	-0.13	0.68	-0.01	2.94	0.2
CPj0339Cpn0339	2	0.15	0.7	0.14	2.55	0.6
