# Supplementary material for: Gene Expression Profiles of Chlamydophila pneumoniae during the Developmental Cycle and Iron Depletion–Mediated Persistence
Source: PLoS Pathog. 2007 Jun 22;3(6):e83. doi: 10.1371/journal.ppat.0030083 (PMC1894823; doi:10.1371/journal.ppat.0030083)
Supplement: Supplementary file 15 [file ppat.0030083.st004.pdf]

Mäurer et. al 2007

Table S4 – EB mRNA Content

Supplementary Table 4

Cpn name	Cluster	EBmRNA
CPn0947CP0912pgsA2	1	Yes
CPn0868CP1001zntA	1	Yes
CPn0035CP0741	2	Yes
CPn0024CP0752xerC	2	Yes
CPn0098htrB	2	Yes
CPn0006	2	Yes
CPn0168CP0603	2	Yes
CPn0028CP0748	2	Yes
CPn0596CP0152ada	2	Yes
CPn0588CP0160	2	Yes
CPn0656CP0091	2	Yes
CPn0679CP0068	2	Yes
CPn0715CP0031gyrB2	2	Yes
CPn0523CP0230	2	Yes
CPn0572CP0177	2	Yes
CPn0682CP0065dppD	2	Yes
CPn0766CP1106	2	Yes
CPn0499CP0255	2	Yes
CPn1011	2	Yes
CPn0944CP0915	2	Yes
CPn0958CP0902plsB	2	Yes
CPn0943CP0917	2	Yes
CPn0772CP1100uvrD	2	Yes
CPn0788CP1084sdhC	2	Yes
CPn0790CP1082sdhB	2	Yes
CPn0837CP1032nth	2	Yes
CPn0843CP1026	2	Yes
CPn0561CP0189euo	2	Yes
CPn0268CP0492	2	Yes
CPn0212CP0553	2	Yes
CPn0266CP0495	2	Yes
CPn0240CP0522	2	Yes
CPn0242CP0520	2	Yes
CPn0406CP0349	2	Yes
CPn0429CP0324nqr4	2	Yes
CPn0641CP0106rs3	3	Yes
CPn0147CP0626	3	Yes
CPn0627CP0120rs11	3	Yes
CPn0026CP0750	3	Yes
CPn0530CP0222spoU1	3	Yes
CPn0351CP0408adt1	4	Yes
CPn0560CP0190gltX	4	Yes
CPn0953CP0906rl9	5	Yes
CPn0644CP0103rl2	5	Yes
CPn0550fusA	5	Yes
CPn0422CP0331	8	Yes
CPn0661mip	8	Yes
CPn0134CP0638groEL1	8	Yes

CPn0621CP0126ruvC	8	Yes
CPn0884CP0982	8	Yes
CPn0709	8	Yes
CPn0710CP0036	8	Yes
CPn0695CP0051ompA	9	Yes
CPn0754CP1118rs20	9	Yes
CPn0876CP0993dagA2	9	Yes
CPn0913CP0953	9	Yes
CPn0135CP0637groES	9	Yes
CPn0194CP0573gcp2	10	Yes
CPn0446CP0307pmp8	10	Yes
CPn0451CP0302pmp11	10	Yes
CPn0796CP1075	10	Yes
CPn0971CP0888yccA2	10	Yes
CPn0933CP0928	10	Yes
CPn0926CP0940	10	Yes
CPn1016CP0837	10	Yes
CPn0001CP0775	10	Yes
CPn1032CP0820	10	Yes
CPn09691yccA1	10	Yes
CPn0886CP0980	10	Yes
CPn0970CP0889tyrP2	10	Yes
CPn0416CP0338himD	10	Yes
CPn0808CP1063	11	Yes
CPn0811CP1060lcrH1	11	Yes
CPn0538CP0214	11	Yes
CPn0587CP0161yvyD	11	Yes
CPn0384CP0371hctB	11	Yes
CPn0445CP0308pmp7	11	Yes
CPn0677CP0070	11	Yes
CPn0742CP0003	11	Yes
CPn0557CP0195omcB	11	Yes
CPn0558CP0193	11	Yes
CPn0383CP0372	11	Yes
CPn0537CP0215	12	Yes
CPn0493CP0261	12	Yes
CPn1006CP0847	12	Yes
CPn0333CP0425	12	Yes
CPn0332	12	Yes
CPn0667CP0080	12	Yes
CPn0473CP0281	12	Yes
CPn0678	12	Yes
CPn0559CP0191	12	Yes
