# Supplementary material for: Gene Expression Profiles of Chlamydophila pneumoniae during the Developmental Cycle and Iron Depletion–Mediated Persistence
Source: PLoS Pathog. 2007 Jun 22;3(6):e83. doi: 10.1371/journal.ppat.0030083 (PMC1894823; doi:10.1371/journal.ppat.0030083)
Supplement: Supplementary file 16 [file ppat.0030083.st005.pdf]

Mäurer et. al 2007

Table S5 – LACK Calculations for EB mRNA Content

Supplementary Table 5

Cluster	SGL	Hits	Size	Hits		p-value
12	42	9	754	91	(P>=9)	=0.05
11	48	11	754	91	(P>=11)	=0.02
10	80	14	754	91	(P>=14)	=0.09
9	50	5	754	91	(P>=5)	=0.73
8	86	7	754	91	(P>=7)	=0.90
7	34	0	754	91	(P>=0)	=1.00
6	55	0	754	91	(P>=0)	=1.00
5	65	3	754	91	(P>=3)	=0.98
4	64	2	754	91	(P>=2)	=0.99
3	53	5	754	91	(P>=5)	=0.78
2	142	33	754	91	(P>=33)	<0.01
1	34	2	754	91	(P>=2)	=0.92
