# Supplementary material for: Gene Expression Profiles of Chlamydophila pneumoniae during the Developmental Cycle and Iron Depletion–Mediated Persistence
Source: PLoS Pathog. 2007 Jun 22;3(6):e83. doi: 10.1371/journal.ppat.0030083 (PMC1894823; doi:10.1371/journal.ppat.0030083)
Supplement: Supplementary file 17 [file ppat.0030083.st006.pdf]

Mäurer et. al 2007

Table S6 – LACK Calculations for EB Proteins

Supplementary Table 6

Cluster	SGL	Hits	Size	Hits		p-value
12	42	4	754	127	(P>=4)	=0.94
11	48	10	754	127	(P>=10)	=0.28
10	80	16	754	127	(P>=16)	=0.27
9	50	15	754	127	(P>=15)	=0.02
8	86	28	754	127	(P>=28)	<0.01
7	34	8	754	127	(P>=8)	=0.20
6	55	8	754	127	(P>=8)	=0.73
5	65	13	754	127	(P>=13)	=0.29
4	64	10	754	127	(P>=10)	=0.65
3	53	7	754	127	(P>=7)	=0.81
2	142	5	754	127	(P>=5)	=0.99
1	34	2	754	127	(P>=2)	=0.99
