# Supplementary material for: Gene Expression Profiles of Chlamydophila pneumoniae during the Developmental Cycle and Iron Depletion–Mediated Persistence
Source: PLoS Pathog. 2007 Jun 22;3(6):e83. doi: 10.1371/journal.ppat.0030083 (PMC1894823; doi:10.1371/journal.ppat.0030083)
Supplement: Supplementary file 18 [file ppat.0030083.st007.pdf]

Mäurer et. al 2007

Table S7 – One-Class SAM Calculations for Persistent Infection at 24hpi

Significant: 73
Median number of false positives: 1.19
False Discovery Rate (%): 1.64

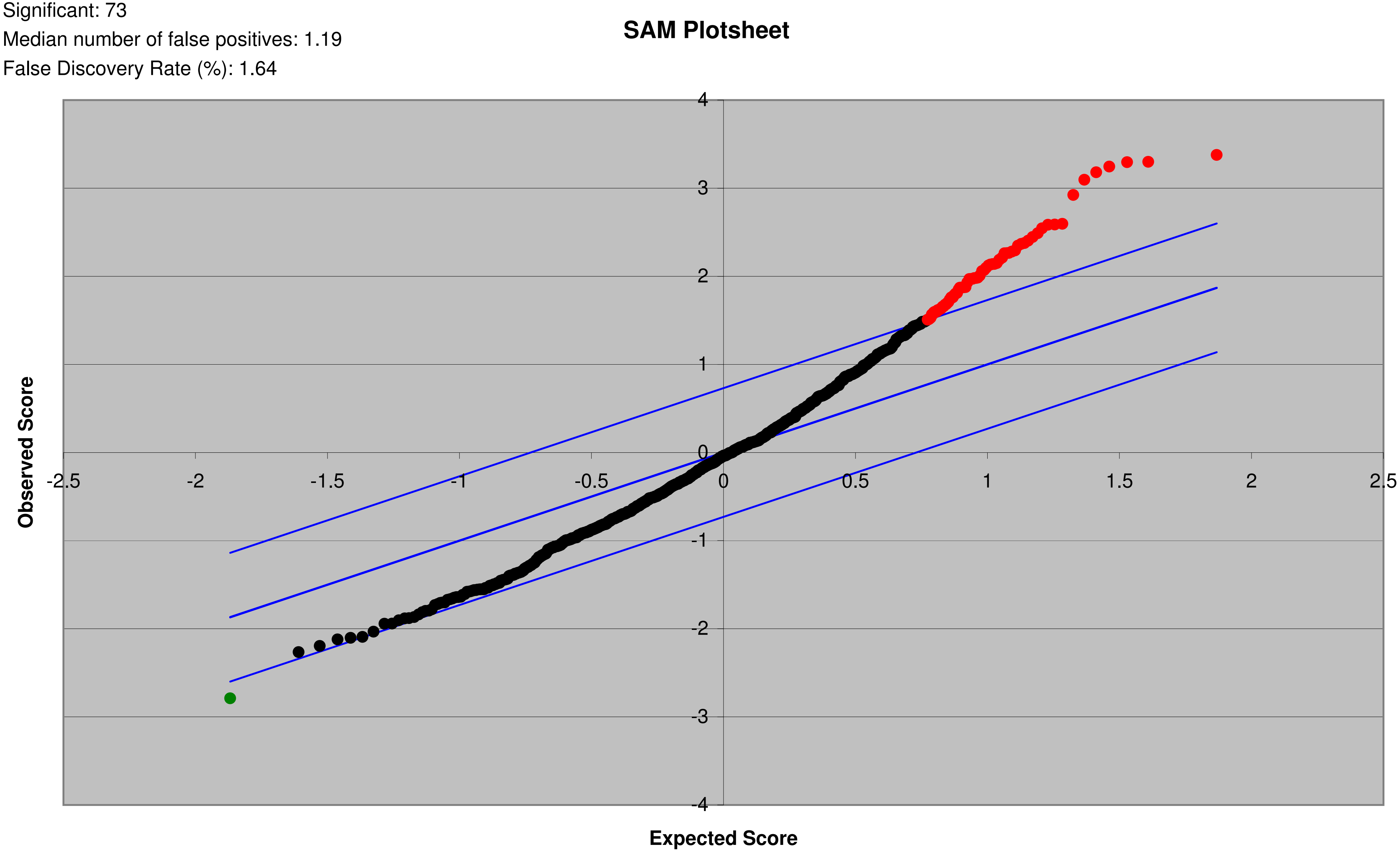

Current settings

Input parameters

**Data type?**	One class
**Arrays centered?**	FALSCH
**Delta**	0.730513142
**Minimum fold change**	0
**Test statistic**	standard
**Are data are log scale?**	FALSCH
**Number of permutations**	100
**Input percentile for exchangeability factor s0**	Automatic choice
**Number of neighbors for KNN**	10
**Seed for Random number generator**	1234567

Computed values

**Estimate of pi0 (proportion of null genes)**	0.596986817
**Exchangibility factor s0**	0.126870643
**s0 percentile**	74.95291902
**False Discovery Rate (%)**	1.635580321

List of Significant Genes for Delta = 0.731

Positive genes (72)

Gene ID	Score(d)	Numerator(r)	Denominator	q-value(%)
CPn0837CP1032nth	3.377633417	1.03214318	0.30558177	0
CPn0404CP0351	3.299635628	0.78404192	0.2376147	0
CPn0960CP0900	3.294858332	0.80717632	0.24498058	0
CPn1018CP0835	3.245125862	0.76002371	0.23420469	0
CPn0108CP0666	3.180097968	0.61640698	0.1938327	0
CPn0588CP0160	3.09566968	0.60024856	0.19389942	0
CPn0465CP0287	2.923639221	0.45951947	0.15717379	0
CPn0948CP0911glgA	2.595857598	0.64569022	0.24873869	0
CPn0240CP0522	2.587941021	0.51365655	0.19848078	0
CPn0038CP0737ptsI	2.584255033	0.55177859	0.21351553	0
CPn0290CP0468	2.544884916	0.45270389	0.17788776	0
CPn0976CP0880	2.488885524	0.51390868	0.20648145	0
CPn0170CP0600	2.446027006	0.47651037	0.19480994	0
CPn0843CP1026	2.404069214	0.39751739	0.16535189	0
CPn0492CP0262	2.376856994	0.47982904	0.20187543	0
CPn0212CP0553	2.367359503	0.5378972	0.22721399	0
CPn0682CP0065dppD	2.347899486	0.49895282	0.2125103	0
CPj0339Cpn0339	2.293291088	0.5897739	0.25717359	0
CPn0024CP0752xerC	2.280169268	0.53773526	0.23583129	0
CPn0102CP0672cydA	2.265288974	0.51420488	0.22699306	0
CPn0859CP1010	2.262391304	0.49845475	0.22032208	0
CPn0020CP0756	2.260813246	0.36208116	0.16015527	0
CPn0066CP0708	2.211298532	0.45069966	0.20381674	0
CPn0579CP0169yacM	2.187533939	0.4349743	0.19884231	0
CPn0470pmp173	2.149695025	0.42514022	0.19776769	0
CPn0328CP0429	2.140374778	0.49249173	0.23009603	0

CPn0679CP0068	2.138358122	0.43974792	0.20564746	0
CPn0559CP0191	2.133317971	0.46091881	0.21605725	0
CPn0190CP0577	2.122963869	0.54075964	0.25471919	0
CPn1073CP0776	2.09410872	0.46687572	0.22294722	0
CPn0143CP0630yxjG1	2.069205681	0.36392084	0.17587466	0
CPn0788CP1084sdhC	2.058373036	0.42142285	0.2047359	0
CPn0596CP0152ada	2.006478006	0.40922744	0.20395311	0
CPn0867CP1002rodA	1.984699292	0.43773531	0.22055498	0
CPn0464CP0288	1.981286384	0.45274036	0.22850829	0
CPn0454CP0298pmp14	1.980043453	0.53938015	0.27240824	0
CPn0149CP0624dnlJ	1.970782102	0.40793228	0.20699004	0
CPn0366CP0391	1.970664251	0.47319529	0.24011969	0
CPn0150CP0623	1.968020477	0.33585909	0.17065833	0
CPn0053CP0722sms	1.928898705	0.33122078	0.17171497	0
CPn0499CP0255	1.877241153	0.42100356	0.22426717	0
CPn0268CP0492	1.876206478	0.60799568	0.32405585	0
CPn0572CP0177	1.873853461	0.42031008	0.22430253	0
CPn0331CP0426	1.871536992	0.36522772	0.19514854	0
CPn0308CP0450	1.870088501	0.35559551	0.19014903	0
CPn0418CP0336murE	1.849820333	0.33334129	0.18020198	0
CPn0473CP0281	1.810602821	0.45811082	0.25301564	0
CPn0281CP0477dhnA	1.802299443	0.62839821	0.34866471	0
CPn0620CP0127ruvA	1.784343162	0.36473868	0.20441062	0
CPn1057CP0793	1.761658082	0.44284141	0.25137762	0
CPn0112CP0661rl31	1.759374122	0.5631388	0.32007905	0
CPn0157CP0614	1.735127249	0.3704593	0.21350555	0
CPn0685CP0061	1.707168588	0.65959386	0.38636715	0
CPn0732nfo	1.696473057	0.41187096	0.24278073	0
CPn0486CP0268	1.682600325	0.37480594	0.22275399	0.865198286
CPn0332	1.678158371	0.41910855	0.24974314	0.865198286
CPn0279CP0479	1.659302868	0.3329424	0.20065198	0.865198286
CPn0159CP0612	1.654986003	0.44493119	0.26884287	0.865198286
CPn0912CP0954	1.635592783	0.38037662	0.23256193	0.865198286
CPn0935CP0926rl34	1.62832456	0.44516335	0.27338736	0.865198286
CPn0380CP0376hemN1	1.61945271	0.40101686	0.24762493	0.865198286
CPn1052CP0800	1.615148008	0.29365708	0.18181434	0.865198286
CPn1024CP0828xerD	1.598905977	0.31514826	0.19710244	0.865198286
CPn0211CP0554	1.598326795	0.35849891	0.22429638	0.865198286
CPn0288CP0470	1.596933055	0.35958402	0.22517163	0.865198286
CPn0493CP0261	1.589916504	0.39174337	0.24639242	0.865198286
CPn0947CP0912pgsA2	1.5701386	0.24307147	0.15480893	0.865198286
CPn0806CP1065thrS	1.56501623	0.43256821	0.27639854	0.865198286
CPn0201CP0566oppD	1.529016735	0.35786816	0.23405118	1.635580321
CPn0034CP0742	1.519375639	0.27088038	0.17828401	1.635580321
CPn0913CP0953	1.511744961	0.32378386	0.21417889	1.635580321
CPn0144CP0629clpB	1.507589324	0.27388276	0.18166934	1.635580321

Estimated Miss rates for Delta=0.730513141968424

Quantiles	Cutpoints	Miss Rate(%)
0 -> 0.05	-2.266 -> -1.4	93.76
0.05 -> 0.1	-1.489 -> -1.1	83.32
0.1 -> 0.15	-1.157 -> -0.9	79.24
0.15 -> 0.2	-0.964 -> -0.8	71.18
0.2 -> 0.25	-0.829 -> -0.6	59.78
0.25 -> 0.75	-0.698 -> 0.4(	9.91
0.75 -> 0.8	0.404 -> 0.58	0
0.8 -> 0.85	0.58 -> 0.748	32
0.85 -> 0.9	0.748 -> 0.93	53.85
0.9 -> 0.95	0.935 -> 1.17	72.85
0.95 -> 1	1.17 -> 1.496	84.03

Significant: 191
Median number of false positives: 35.52
False Discovery Rate (%): 18.6

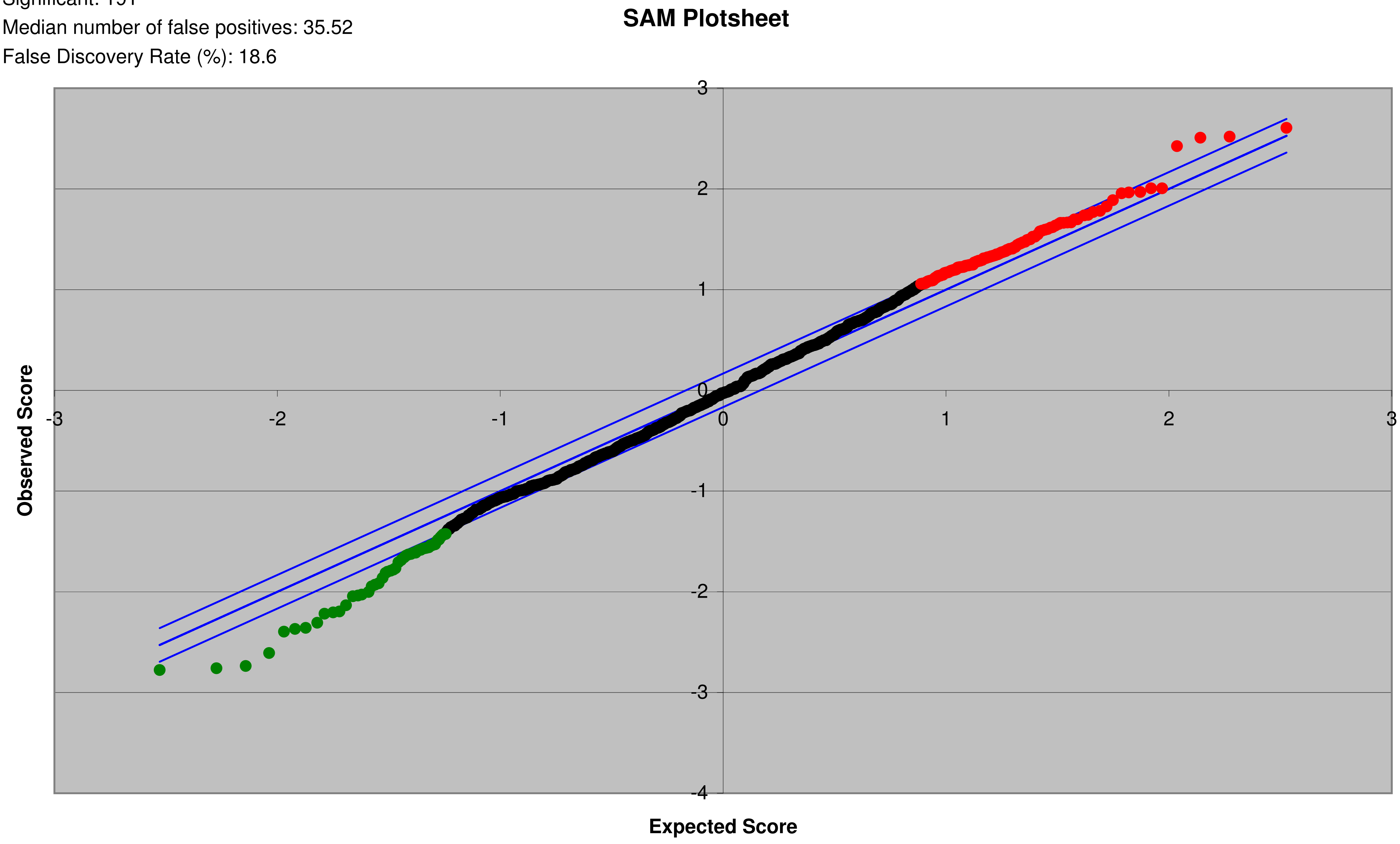

Current settings

Input parameters

**Data type?**	One class
**Arrays centered?**	FALSCH
**Delta**	0.16730977
**Minimum fold change**	0
**Test statistic**	standard
**Are data are log scale?**	FALSCH
**Number of permutations**	100
**Input percentile for exchangeability factor s0**	Automatic choice
**Number of neighbors for KNN**	10
**Seed for Random number generator**	1234567

Computed values

**Estimate of pi0 (proportion of null genes)**	0.71751412
**Exchangibility factor s0**	0.0813116
**s0 percentile**	10.0753296
**False Discovery Rate (%)**	18.5952613

List of Significant Genes for Delta = 0.167

Positive genes (139)

Gene ID	Score(d)	Numerator	Denominat	q-value(%	localfdr(%)
CPn0152CP0620	2.60635784	0.335131	0.128582	3.587571	16.43838
CPn0439CP0314	2.51915096	0.33322	0.132275	3.587571	16.11938
CPn0429CP0324nqr4	2.50825145	0.463608	0.184833	3.587571	16.08113
CPn1072CP0777	2.42428078	0.449968	0.185609	3.587571	15.80901
CPn0514CP0239	2.00597154	0.329268	0.164144	3.587571	15.46309
CPn0513CP0240	2.005444	0.278866	0.139055	3.587571	15.46495
CPj0339Cpn0339	1.96922353	0.577135	0.293078	3.587571	15.60483
CPn0565CP0185	1.96479084	0.285941	0.145533	3.587571	15.6234
CPn0201CP0566oppD	1.95578412	0.580697	0.296913	3.587571	15.66201
CPn0517CP0236	1.88828353	0.44091	0.233498	3.587571	15.9864
CPn0873CP0996ribE	1.82478841	0.248065	0.135942	3.587571	16.34617
CPn0842	1.78157801	0.347329	0.194956	3.587571	16.61569
CPn0198CP0569oppA4	1.76957268	0.333831	0.188651	3.587571	16.69375
CPn0495CP0259aspC	1.74113473	0.354141	0.203397	3.587571	16.88591
CPn0219CP0546tgt	1.73715986	0.358363	0.206292	18.59526	16.91372
CPn0241CP0521	1.69734153	0.338748	0.199576	18.59526	17.20838
CPn0268CP0492	1.69655506	0.937284	0.552463	18.59526	17.21453
CPn0059CP0716dut	1.66668874	0.162866	0.097718	18.59526	17.45915
CPn0055CP0720	1.66546586	0.212501	0.127593	18.59526	17.46968
CPn0108CP0666	1.66287775	0.641526	0.385793	18.59526	17.49211
CPn0024CP0752xerC	1.66206851	0.802666	0.482932	18.59526	17.49917
CPn0054CP0721rnc	1.65056668	0.265968	0.161137	18.59526	17.60182
CPn0464CP0288	1.63526334	0.808246	0.49426	18.59526	17.74582
CPn0936CP0925rl36	1.6202181	0.312557	0.192911	18.59526	17.89676
CPn0783CP1089	1.61558768	0.719523	0.445363	18.59526	17.94526
CPn0335folD	1.6013807	0.395103	0.246726	18.59526	18.10053

CPn0912CP0954	1.59362732	0.291122	0.182679	18.59526	18.1896
CPn0123CP0650recD1	1.58516282	0.474883	0.29958	18.59526	18.29056
CPn0151CP0622mhpA	1.57817056	0.300046	0.190123	18.59526	18.377
CPn0020CP0756	1.54828811	0.469083	0.302969	18.59526	18.77999
CPn0586CP0162atoC	1.52555724	0.237823	0.155892	18.59526	19.12654
CPn0026CP0750	1.52433356	0.352644	0.231343	18.59526	19.14626
CPn0442CP0311	1.49916071	0.334376	0.223042	18.59526	19.57752
CPn0223CP0542	1.49637481	0.491477	0.328445	18.59526	19.62837
CPn0328CP0429	1.4910055	0.679869	0.45598	18.59526	19.72823
CPn0053CP0722sms	1.47504625	0.491036	0.332895	18.59526	20.03972
CPn0098htrB	1.46872494	0.35946	0.244743	18.59526	20.16939
CPn0271CP0488	1.45927712	0.268076	0.183705	18.59526	20.37009
CPn0025CP0751atsA	1.45402414	0.517356	0.35581	18.59526	20.48534
CPn0588CP0160	1.44146225	0.492911	0.341952	18.59526	20.77188
CPn0113CP0660pfrA	1.42349686	0.337339	0.236979	18.59526	21.20949
CPn1027CP0825	1.41856055	0.332725	0.234551	18.59526	21.33568
CPn0067CP0707	1.40602729	0.171324	0.12185	18.59526	21.66799
CPn0132CP0640	1.40587439	0.349406	0.248533	18.59526	21.67215
CPn0170CP0600	1.40397417	0.491257	0.349904	18.59526	21.72409
CPn0008CP0766	1.39448216	0.41033	0.294253	18.59526	21.98967
CPn0370CP0387	1.39366219	0.362083	0.259807	18.59526	22.0131
CPn0158CP0613	1.38191812	0.404112	0.292428	18.59526	22.35734
CPn0882CP0984	1.37806076	0.298036	0.216272	18.59526	22.47399
CPn0461CP0291	1.372361	0.371023	0.270354	18.59526	22.64969
CPn0176CP0593	1.37059532	0.528307	0.385458	18.59526	22.70492
CPn0254CP0507	1.36290045	0.210831	0.154693	18.59526	22.95019
CPn0404CP0351	1.35878195	1.025378	0.75463	18.59526	23.08454
CPn0245CP0517ydhO	1.35001706	0.210377	0.155833	18.59526	23.37771
CPn0261CP0500ydaO	1.34946262	0.646617	0.479166	18.59526	23.39659
CPn0844CP1025yphC	1.34146316	0.36173	0.269653	18.59526	23.67348
CPn0066CP0708	1.33797393	0.841998	0.629308	18.59526	23.79691
CPn0515CP0238ubiE	1.33317004	0.442471	0.331894	18.59526	23.96949
CPn0580CP0168truA	1.33146003	0.424126	0.318542	18.59526	24.03167
CPn0837CP1032nth	1.32513813	1.050087	0.792436	18.59526	24.26499
CPn0009CP0765	1.32132553	0.543524	0.411347	18.59526	24.40833
CPn0190CP0577	1.3199585	0.444336	0.336629	18.59526	24.46021
CPn0159CP0612	1.31331841	0.505168	0.38465	18.59526	24.71588
CPn0999CP0855pnp	1.31089895	0.261858	0.199755	18.59526	24.81055
CPn0813CP1058pepP	1.3094107	0.265272	0.202589	18.59526	24.8692
CPn0257CP0504	1.29422386	0.348963	0.269631	18.59526	25.4855
CPn0255CP0506	1.29158488	0.443232	0.343169	18.59526	25.59595
Cpn0061_CP0714	1.28659723	0.322631	0.250763	18.59526	25.80744
CPn0197CP0570oppA3	1.28600047	0.256592	0.199527	18.59526	25.83299
CPn0418CP0336murE	1.28000745	0.494783	0.386547	18.59526	26.09242
CPn0492CP0262	1.27821444	0.713333	0.55807	18.59526	26.17107
CPn0403yceC	1.27090927	0.557298	0.438503	18.59526	26.49639
CPn1069CP0781yfgA	1.2680209	0.155391	0.122546	18.59526	26.6272
CPn0578CP0170yaeI	1.24713327	0.165073	0.132362	18.59526	27.61078
CPn0035CP0741	1.24651157	0.706179	0.566524	18.59526	27.64108
CPn0375CP0381	1.24475947	0.262629	0.210988	18.59526	27.72679
CPn0496CP0258	1.24393581	0.204576	0.164459	18.59526	27.76725
CPn0225CP0539	1.24051347	0.481562	0.388196	18.59526	27.93647
CPn0927CP0939	1.2400287	0.296013	0.238715	18.59526	27.96059
CPn0596CP0152ada	1.23744637	0.310465	0.250891	18.59526	28.08968
CPn0137CP0635ybgI	1.23642632	0.388247	0.314007	18.59526	28.14095
CPn0577CP0171	1.2338332	0.189729	0.153772	18.59526	28.27203
CPn0867CP1002rodA	1.22767852	0.531259	0.432735	18.59526	28.5873
CPn0003CP0772gatA	1.22523633	0.32753	0.26732	18.59526	28.71403
CPn0331CP0426	1.22361362	0.446684	0.365053	18.59526	28.79875
CPn0470pmp173	1.2235217	0.438948	0.358758	18.59526	28.80356
CPn0102CP0672cydA	1.2233442	0.557724	0.455901	18.59526	28.81286
CPn0862CP1007yfhO2	1.22118246	0.416619	0.341161	18.59526	28.92644
CPn1053CP0799	1.22083492	0.402266	0.329501	18.59526	28.94477

CPn1073CP0776	1.22016761	0.47243	0.387185	18.59526	28.98001
CPn0773CP1099ung	1.2170903	0.269704	0.221597	18.59526	29.14344
CPn0843CP1026	1.2022525	0.356303	0.296363	18.59526	29.95211
CPn1036CP0816aroB	1.20020235	0.185756	0.15477	18.59526	30.06654
CPn0209CP0557	1.19688032	0.23777	0.198658	18.59526	30.25334
CPn1035CP0817aroE	1.19606543	0.316648	0.264742	18.59526	30.29943
CPn0790CP1082sdhB	1.1947663	0.404311	0.338402	18.59526	30.37312
CPn0406CP0349	1.1918362	0.26988	0.226441	18.59526	30.54028
CPn0618CP0129lplA2	1.18778439	0.452893	0.381292	18.59526	30.77363
CPn0522CP0231	1.18739826	0.545858	0.45971	18.59526	30.796
CPn0376CP0380	1.18086127	0.154466	0.130808	18.59526	31.17818
CPn0366CP0391	1.17627865	0.539734	0.458848	18.59526	31.44996
CPn1034CP0818	1.17238474	0.247901	0.211451	18.59526	31.68337
CPn0980CP0876	1.17127967	0.194713	0.166239	18.59526	31.75002
CPn0149CP0624dnlJ	1.16992739	0.589345	0.503745	18.59526	31.83182
CPn0163CP0608	1.16845994	0.387739	0.331838	18.59526	31.9209
CPn0948CP0911glgA	1.16537158	0.435859	0.374009	18.59526	32.10939
CPn0131CP0641	1.16350219	0.681065	0.585358	18.59526	32.22416
CPn0175CP0594	1.15453588	0.234526	0.203134	18.59526	32.78163
CPn1065	1.14706471	0.730976	0.637258	18.59526	33.25482
CPn0041CP0733	1.14601832	0.320936	0.280045	18.59526	33.32171
CPn0682CP0065dppD	1.14500875	0.636676	0.556045	18.59526	33.38639
CPn0463CP0289	1.13920213	0.34549	0.303273	18.59526	33.76113
CPn0380CP0376hemN1	1.13756727	0.579376	0.509311	18.59526	33.86746
CPn0679CP0068	1.13639616	0.547404	0.481701	18.59526	33.94385
CPn0303CP0455	1.13599517	0.57512	0.506269	18.59526	33.97005
CPn0006	1.13281468	0.463225	0.408915	18.59526	34.1786
CPn0988CP0867murB	1.12430122	0.15605	0.138797	18.59526	34.74343
CPn0281CP0477dhnA	1.11905196	0.467614	0.417867	18.59526	35.09639
CPn0732nfo	1.11740968	0.375296	0.335863	18.59526	35.20754
CPn0288CP0470	1.10943849	0.488028	0.439887	18.59526	35.75184
CPn1018CP0835	1.09795509	0.793539	0.722743	18.59526	36.54974
CPn0888CP0978hemG	1.09207735	0.197219	0.18059	18.59526	36.96426
CPn0976CP0880	1.0918974	0.656479	0.601228	18.59526	36.97701
CPn0235CP0527kdsB	1.08953089	0.185595	0.170344	18.59526	37.1451
CPn0246CP0516rs9	1.08880992	0.27875	0.256013	18.59526	37.19644
CPn0334CP0424	1.08845681	0.339149	0.311587	18.59526	37.22161
CPn0788CP1084sdhC	1.08735837	0.458946	0.422074	18.59526	37.29998
CPn0150CP0623	1.08519342	0.885511	0.815994	18.59526	37.45487
CPn0730CP0016mviN	1.08262606	0.488748	0.451447	18.59526	37.63923
CPn0212CP0553	1.08045816	0.728996	0.67471	18.59526	37.79549
CPn0285CP0473	1.07262931	0.243938	0.22742	18.59526	38.3641
CPn0579CP0169yacM	1.07079392	0.276468	0.25819	18.59526	38.49836
CPn0734yceA	1.06321601	0.150852	0.141883	18.59526	39.05643
CPn0697CP0049tsf	1.06264735	0.184904	0.174003	18.59526	39.09854
CPn0043CP0730	1.06061444	0.381355	0.359561	18.59526	39.24936
CPn0448CP0305yxjG2	1.06044316	0.310827	0.293111	18.59526	39.26209
CPn0696rs2	1.05946611	0.200312	0.189069	18.59526	39.33474
CPn0542CP0210	1.05731936	0.22157	0.209559	18.59526	39.4947
CPn0465CP0287	1.05689785	0.612667	0.579684	18.59526	39.52616

Negative genes (52)

Gene ID	Score(d)	Numerator	Denominat	q-value(%	localfdr(%)
CPn0503CP0251dnaK	-2.7768358	-0.618048	0.222573	0	9.430365
CPn0084CP0691	-2.7592558	-0.754956	0.273608	0	9.542052
CPn0655CP0092dnaQ2	-2.7370254	-0.482148	0.176158	0	9.682457
CPn0985CP0871nrdB	-2.6088296	-0.653209	0.250384	0	10.40143
CPn0390CP0365ruvB	-2.3953852	-0.335294	0.139975	0	10.68354
CPn0105CP0669	-2.3687428	-0.439411	0.185504	0	10.6408
CPn0165CP0606	-2.3581423	-0.530622	0.225017	0	10.62236
CPn0779CP1093	-2.3071541	-0.480381	0.208214	0	10.53466
CPn0309CP0449dnaA1	-2.2177153	-0.310731	0.140113	0	10.4525

CPn0402CP0353mutY	-2.2043383	-0.23149	0.105016	0	10.45613
CPn0318CP0439rbfA	-2.195254	-0.38639	0.176012	0	10.46189
CPn0363CP0394flhA	-2.1344631	-0.673444	0.31551	0	10.58103
CPn0575aprfB	-2.0446614	-0.321006	0.156997	4.484463	11.05451
CPn0039CP0736ybaB	-2.0374498	-0.267565	0.131324	4.484463	11.10991
CPn0085CP0690	-2.0277648	-0.273665	0.134959	4.484463	11.18869
CPn0433CP0320gcsH	-2.0017307	-0.336947	0.168328	4.484463	11.42619
CPn0995CP0860	-1.9463824	-0.54584	0.280438	4.484463	12.06649
CPn0731CP0015	-1.9304078	-0.766787	0.397215	4.484463	12.28871
CPn0389CP0366	-1.9153793	-0.281698	0.147072	4.484463	12.51422
CPn0044CP0729	-1.8630367	-0.250935	0.134691	4.484463	13.42821
CPn0660spoU2	-1.8138508	-0.240006	0.132319	5.125101	14.45811
CPn0072CP0703	-1.8010191	-0.412067	0.228797	5.125101	14.75154
CPn0897CP0969	-1.7903524	-0.436236	0.243659	5.125101	15.00272
CPn0657CP0090	-1.7806532	-0.295225	0.165796	5.125101	15.23663
CPn0154CP0617gseA	-1.7673394	-0.30981	0.175297	6.185467	15.56595
CPn0685CP0061	-1.7069581	-0.32732	0.191756	7.175141	17.16831
CPn0626CP0121rpoA	-1.6857753	-0.374962	0.222427	7.175141	17.76815
CPn0822CP1049	-1.6586228	-0.531888	0.32068	7.175141	18.56191
CPn0780CP1092amiB	-1.6416902	-0.372409	0.226845	7.175141	19.06978
CPj05762CPn0576	-1.6309534	-0.940024	0.576365	7.175141	19.3965
CPn1017CP0836lytB	-1.6251402	-0.241676	0.148711	7.175141	19.57483
CPn0611CP0136yacE	-1.6142432	-0.46747	0.289591	10.46375	19.9117
CPn0921CP0945	-1.6114726	-0.448276	0.278178	10.46375	19.99788
CPn0755CP1117	-1.593957	-0.356993	0.223966	10.46375	20.54723
CPn0070	-1.5867264	-0.568301	0.358159	10.46375	20.77619
CPn1013CP0840fumC	-1.5831033	-0.494733	0.312509	10.46375	20.89136
CPn0502CP0252grpE	-1.5700856	-0.296289	0.188709	10.46375	21.30752
CPn0884CP0982	-1.5668582	-0.527248	0.3365	10.46375	21.4112
CPn0434CP0319	-1.5628162	-0.205559	0.131531	10.46375	21.54132
CPn0632CP0115rl18	-1.5587782	-0.365804	0.234674	10.46375	21.67158
CPn1059CP0791kgsA	-1.538826	-0.364324	0.236754	10.46375	22.31843
CPn0558CP0193	-1.5385881	-0.703202	0.457044	10.46375	22.32617
CPn0386CP0369ssb	-1.5313033	-0.360118	0.235171	10.46375	22.56339
CPn0282CP0476xasA	-1.529584	-0.37693	0.246427	10.46375	22.61944
CPn1023CP0829	-1.5016666	-0.540077	0.359652	10.98236	23.53096
CPn0740tyrB	-1.4844271	-0.267635	0.180295	12.66201	24.0934
CPn1031CP0821arcD	-1.4816216	-1.32535	0.894527	12.66201	24.18477
CPn1007CP0846	-1.4601202	-0.368428	0.252327	12.66201	24.88233
CPn0869CP1000	-1.4455096	-0.293049	0.202731	12.66201	25.35275
CPn0384CP0371hctB	-1.4310584	-1.391409	0.972293	12.81275	25.81433
CPn0750CP1122tctD	-1.4271158	-0.88636	0.621085	12.81275	25.93952
CPn0416CP0338himD	-1.4261572	-0.637402	0.446937	12.81275	25.9699

timated Miss rates for Delta=0.167309774990(

Quantiles	Cutpoints	Miss Rate(%)
0 -> 0.05	-1.404 -> -1	40.87
0.05 -> 0.1	-1.1 -> -0.9	65.95
0.1 -> 0.15	-0.985 -> -(	64.05
0.15 -> 0.2	-0.891 -> -(	47.57
0.2 -> 0.25	-0.782 -> -(	35.26
0.25 -> 0.7!	-0.668 -> 0	5.01
0.75 -> 0.8	0.434 -> 0.!	26.4
0.8 -> 0.85	0.533 -> 0.(	15.11
0.85 -> 0.9	0.66 -> 0.7(	40.09
0.9 -> 0.95	0.764 -> 0.8	39.46
0.95 -> 1	0.886 -> 1.(	42.54

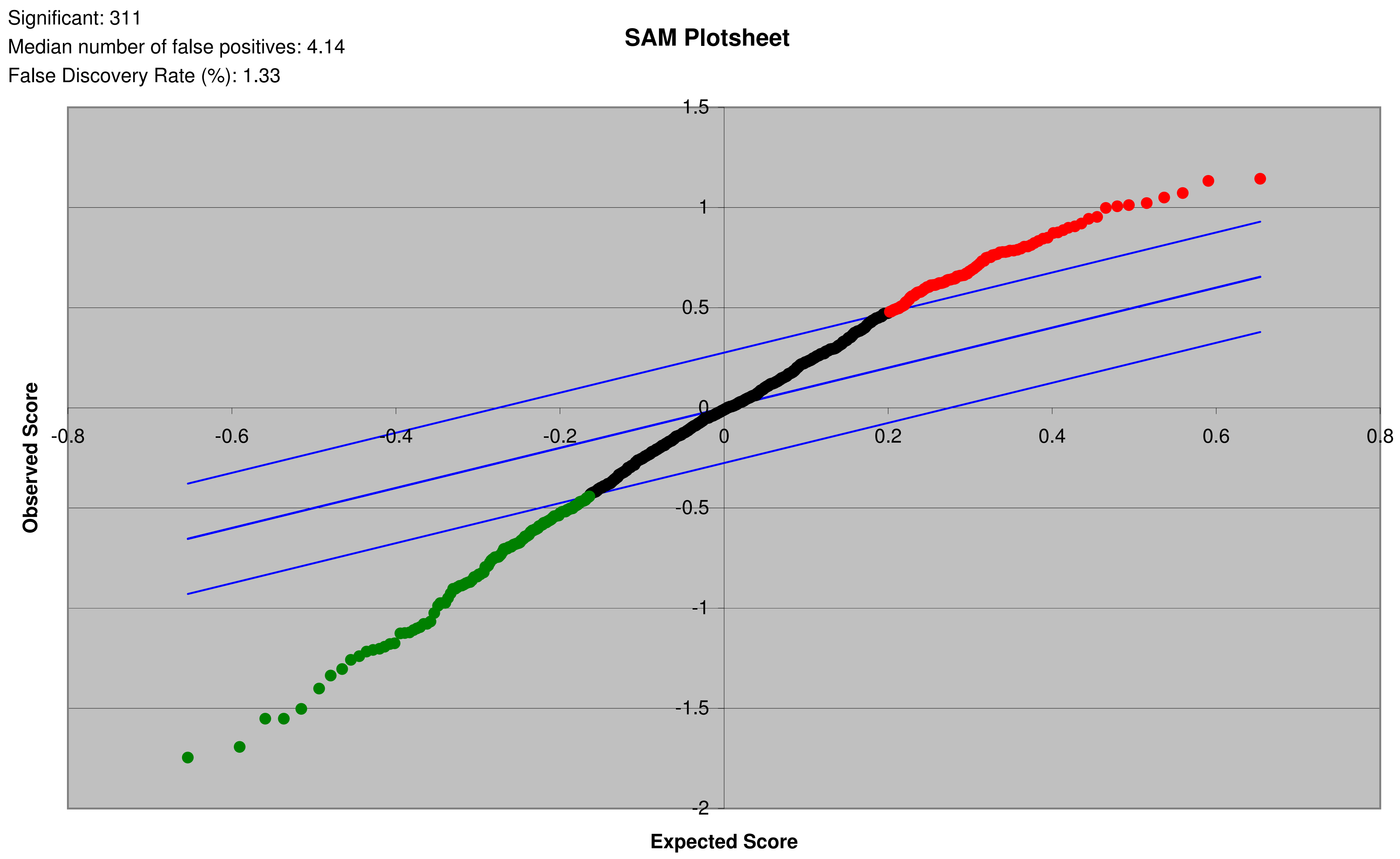

Significant: 311
Median number of false positives: 4.14
False Discovery Rate (%): 1.33
SAM Plotsheet
1.5
1
0.5
0
-0.5
-1
-1.5
-2
-0.8
-0.6
-0.4
-0.2
0
0.2
0.4
0.6
0.8
Observed Score
Expected Score

Current settings

Input parameters

**Data type?**	One class
**Arrays centered?**	FALSCH
**Delta**	0.275575943
**Minimum fold change**	0
**Test statistic**	standard
**Are data are log scale?**	FALSCH
**Number of permutations**	100
**Input percentile for exchangeability factor s0**	Automatic choice
**Number of neighbors for KNN**	10
**Seed for Random number generator**	1234567

Computed values

**Estimate of pi0 (proportion of null genes)**	0.414312618
**Exchangibility factor s0**	1.301766271
**s0 percentile**	99.90583804
**False Discovery Rate (%)**	1.332194912

List of Significant Genes for Delta = 0.276

Positive genes (128)

Gene ID	Score(	Numerator	Denomir	q-value(%)	localfdr(%)
CPn0404CP0351	1.143	1.695609	1.48308	0	0.2613
CPn0353CP0406	1.133	1.700759	1.5013	0	0.23351
CPn0837CP1032nth	1.072	1.741598	1.62477	0	0.0682
CPn0038CP0737ptsI	1.049	1.743096	1.66136	0	0.00315
CPn1018CP0835	1.022	1.492626	1.46063	0	0
CPn0028CP0748	1.011	1.418672	1.40262	0	0
CPn0960CP0900	1.006	1.502066	1.49351	0	0
CPn0212CP0553	0.998	1.439038	1.44236	0	0
CPn0465CP0287	0.953	1.393339	1.46237	0	0
CPn0902CP0964nlpD	0.943	1.367647	1.45027	0	0
CPn0279CP0479	0.92	1.22913	1.3366	0	0
CPn0240CP0522	0.906	1.25216	1.38238	0	0
CPn0830CP1041	0.899	1.29847	1.44495	0	0
CPn0403yceC	0.887	1.291139	1.45597	0	0
CPn1001CP0852yfhC	0.875	1.338135	1.52861	0	0
CPn0664	0.873	1.290334	1.47855	0	0
CPn0522CP0231	0.849	1.201965	1.41572	0	0
CPn0280CP0478dppF1	0.844	1.19752	1.41815	0	0
CPn0303CP0455	0.832	1.140807	1.3704	0	0
CPn0066CP0708	0.822	1.241097	1.50906	0	0
CPn0682CP0065dppD	0.813	1.153518	1.41971	0	0
CPn0218CP0547	0.805	1.28291	1.59362	0.3236817	0
CPn0168CP0603	0.804	1.207355	1.50164	0.3236817	0
CPn1015CP0838	0.795	1.120957	1.41087	0.3236817	0
CPn0102CP0672cydA	0.789	1.151493	1.45944	0.3236817	0
CPn0004CP0771gatB	0.785	1.12836	1.4383	0.3236817	0

CPn0288CP0470	0.784	1.075417	1.37103	0.3236817	0
CPn0554CP0198	0.78	1.056512	1.35424	0.3236817	0
CPn0961CP0899rl32	0.778	1.097956	1.41175	0.3236817	0
CPn0979CP0877htrA	0.778	1.153836	1.48386	0.3236817	0
CPn0046CP0728	0.776	1.116886	1.43994	0.3236817	0
CPn0679CP0068	0.767	1.149881	1.49923	0.3236817	0
CPn0006	0.764	1.10597	1.44757	0.3236817	0
CPn0464CP0288	0.762	1.098473	1.44238	0.3236817	0
CPn0047	0.752	1.075486	1.43105	0.3236817	0
CPn1063CP0786tpiS	0.751	1.047814	1.39492	0.3236817	0
CPn0807CP1064	0.748	1.068285	1.42809	0.3236817	0
CPn0009CP0765	0.734	1.099577	1.49883	0.3236817	0
CPn0268CP0492	0.731	1.059463	1.44864	0.3236817	0
CPn0035CP0741	0.72	1.10016	1.5277	0.3236817	0
CPn0981CP0875	0.71	1.063347	1.49711	0.3236817	0
CPn09691yccA1	0.703	1.045988	1.48711	0.3236817	0
CPn0131CP0641	0.694	0.99228	1.42941	0.5380683	0
CPn0024CP0752xerC	0.69	0.993212	1.4399	0.5380683	0
CPn0264CP0497ubiD	0.684	0.93068	1.36132	0.5380683	0
CPn0753CP1119	0.68	1.022591	1.5033	0.5380683	0
CPn0976CP0880	0.67	0.983187	1.46683	0.5380683	0
CPn0783CP1089	0.67	1.050446	1.56775	0.5380683	0
CPn0348CP0412ytgB1	0.662	0.947751	1.43219	0.5380683	0
CPn0261CP0500ydaO	0.661	0.992806	1.50125	0.5380683	0
CPn1038CP0814aroL	0.66	0.943553	1.43012	0.5380683	0
CPn0086_CP0688	0.659	0.966742	1.46624	0.5380683	0
CPn0938CP0923	0.657	0.937766	1.42822	0.5380683	0
CPn1066CP0784	0.655	0.936355	1.42852	0.5380683	0
CPn0778CP1094ahpC	0.646	0.943388	1.46003	0.5380683	0
CPn0787CP1085yabD	0.644	0.894998	1.38971	0.5380683	0
CPn0895CP0971efp2	0.644	0.907542	1.4094	0.5380683	0
CPn1053CP0799	0.642	1.034047	1.6111	0.5380683	0
CPn0871CP0998ribD	0.64	0.877604	1.3721	0.5380683	0
CPn0008CP0766	0.639	0.882555	1.38114	0.5380683	0
CPn0545CP0207rl27	0.638	0.974499	1.52713	0.5380683	0
CPn0266CP0495	0.636	0.938857	1.47579	0.5380683	0
CPn0806CP1065thrS	0.628	0.904982	1.44038	0.6829329	0
CPn0829CP1042	0.627	0.935905	1.49208	0.6829329	0
CPn0798CP1073	0.624	0.872396	1.39811	0.6829329	0
CPn0867CP1002rodA	0.623	0.895514	1.43805	0.6829329	0
CPn0654CP0093yciA	0.622	0.863556	1.38749	0.6829329	0
CPn1065	0.622	0.905751	1.45539	0.6829329	0
CPn0250CP0511rl33	0.619	0.91769	1.48201	0.6829329	0
CPn0034CP0742	0.616	0.872958	1.41691	0.6829329	0
CPn0211CP0554	0.613	0.884003	1.44137	0.6829329	0
CPn0658	0.613	0.904398	1.47493	0.6829329	0
CPn0492CP0262	0.613	0.970993	1.58505	0.6829329	0
CPn1055CP0795	0.612	0.863975	1.41145	0.6829329	0
CPn0112CP0661rl31	0.612	0.887599	1.4511	0.6829329	0
CPn1030CP0822	0.612	0.942185	1.54071	0.6829329	0
CPn0529CP0223ycaH	0.606	0.889867	1.46743	0.6829329	0
CPn0418CP0336murE	0.603	0.916111	1.51883	0.6829329	0
CPn0618CP0129lplA2	0.603	0.875225	1.45258	0.6829329	0
CPn0831CP1039	0.602	0.869464	1.44331	0.6829329	0
CPn0380CP0376hemN1	0.599	0.868541	1.45103	0.8286252	0
CPn1064CP0785	0.595	0.846545	1.42299	0.8286252	0.0037
CPn0260CP0501secA1	0.593	0.863122	1.45585	0.8286252	0.05601
CPn0947CP0912pgsA2	0.587	0.860549	1.46697	0.8286252	0.2249
CPn0997CP0858mesJ	0.583	0.829402	1.42159	0.8286252	0.31624
CPn0990CP0865infC	0.581	0.831217	1.43078	0.9331365	0.38993
CPn0358CP0400	0.578	0.826283	1.42929	0.9331365	0.47719
CPn0290CP0468	0.578	0.814551	1.40988	0.9331365	0.48861
CPn1067CP0783def	0.577	0.819485	1.41919	0.9331365	0.49844

CPn0043CP0730	0.576	0.801577	1.39194	0.9331365	0.54803
CPn0277CP0481	0.576	0.822175	1.4285	0.9331365	0.55818
CPn0149CP0624dnlJ	0.571	0.844259	1.47895	0.9331365	0.71373
CPn0723yhbG	0.569	0.878903	1.54435	0.9331365	0.77353
CPn0002CP0773gatC	0.563	0.82751	1.47056	0.9331365	1.00332
CPn0528CP0224gltT	0.56	0.75022	1.34012	0.9331365	1.113
CPn0355CP0404	0.558	0.778032	1.39339	1.0357815	1.16899
CPn0263CP0498yqfU	0.558	0.789216	1.41534	1.0357815	1.19865
CPn0891CP0975mfd	0.557	0.784224	1.40893	1.0357815	1.23849
CPn0293CP0465	0.552	0.774116	1.40348	1.0357815	1.44446
CPn0971CP0888yccA2	0.548	0.854867	1.5591	1.0357815	1.58373
CPn0909CP0957rsbV2	0.539	0.786728	1.45869	1.0519656	1.99149
CPn1050CP0802dapA	0.537	0.783866	1.45856	1.0519656	2.0832
CPn0788CP1084sdhC	0.532	0.796448	1.49686	1.0519656	2.34902
CPn0539CP0213pmp19	0.529	0.738861	1.39696	1.0781369	2.51325
CPn1058CP0792	0.528	0.761701	1.44198	1.0781369	2.5488
CPn0866CP1003birA	0.525	0.728882	1.38887	1.0781369	2.73339
CPn0732nfo	0.513	0.733768	1.42942	1.1508684	3.394
CPn1037CP0815aroC	0.512	0.709383	1.38593	1.1508684	3.48491
CPn0170CP0600	0.509	0.739091	1.45145	1.1508684	3.64871
CPn0092CP0682atpK	0.509	0.69268	1.36033	1.1508684	3.64932
CPn0310CP044860IM	0.505	0.734078	1.45332	1.1508684	3.91161
CPn0244CP0518adk	0.505	0.695175	1.37643	1.1508684	3.9146
CPn0357CP0401	0.503	0.753765	1.4986	1.1508684	4.05125
CPn0767CP1105	0.497	0.704923	1.41921	1.1508684	4.47903
CPn0256CP0505	0.497	0.669877	1.34903	1.1508684	4.48896
CPn0620CP0127ruvA	0.496	0.748733	1.51048	1.1508684	4.54992
CPn0887CP0979	0.496	0.688901	1.39026	1.1508684	4.56225
CPn0231CP0533tauB	0.493	0.713678	1.44883	1.1508684	4.77183
CPn1039CP0813aroA	0.493	0.701829	1.42494	1.1508684	4.77586
CPn0408CP0346	0.491	0.68145	1.38789	1.1508684	4.88767
CPn0613CP0134sohB	0.489	0.679127	1.38774	1.3321949	5.0074
CPn0986CP0869yggH	0.489	0.716846	1.46503	1.3321949	5.01276
CPn0999CP0855pnp	0.487	0.70858	1.4556	1.3321949	5.20117
CPn0327CP0430rl28	0.485	0.721827	1.48917	1.3321949	5.36033
CPn0862CP1007yfhO2	0.484	0.688668	1.42279	1.3321949	5.41365
CPn1029CP0823	0.48	0.691507	1.43924	1.3321949	5.69402
CPn0337CP0421smpB	0.48	0.72222	1.50605	1.3321949	5.76763
CPn0158CP0613	0.479	0.700555	1.46273	1.3321949	5.817

Negative genes (183)

Gene ID	Score(	Numerator	Denomir	q-value(%)	localfdr(%)
CPn0676CP0071	-1.75	-2.541974	1.45606	0	0
CPn0852CP1017	-1.69	-2.371182	1.40077	0	0
CPn0065CP0709	-1.55	-2.142925	1.38083	0	0.00234
CPn0728CP0018	-1.55	-2.248637	1.44899	0	0.00235
CPn0330CP0427	-1.5	-2.150901	1.43108	0	0.00676
CPn0472CP0282	-1.4	-1.957647	1.39688	0	0.0213
CPn0727CP0019	-1.34	-2.135874	1.59819	0	0.03382
CPn0677CP0070	-1.3	-1.969001	1.50983	0	0.04011
CPn0994CP0861	-1.26	-1.976432	1.57103	0	0.04752
CPn1005CP0848	-1.24	-1.857571	1.49793	0	0.04942
CPn0538CP0214	-1.22	-1.803914	1.48251	0	0.05054
CPn0524CP0229	-1.21	-1.860159	1.5391	0	0.05047
CPn0725	-1.2	-1.680347	1.39722	0	0.05025
CPn0228CP0536dsbG	-1.19	-1.663741	1.39471	0	0.04955
CPn0233CP0531	-1.18	-2.687504	2.27967	0	0.04773
CPn0808CP1063	-1.18	-1.899613	1.61557	0	0.04718
CPn0332	-1.13	-1.772285	1.57416	0	0.02923
CPn0537CP0215	-1.12	-1.623406	1.44407	0	0.02829
CPn1023CP0829	-1.12	-1.607149	1.43349	0	0.02649
CPn0810CP1061	-1.11	-1.847056	1.6643	0	0.01904

CPn1004CP0849	-1.1	-1.727618	1.5686	0	0.01265
CPn0853CP1016	-1.09	-1.672121	1.52749	0	0.00703
CPn0227CP0537dsbB	-1.08	-1.500781	1.39132	0	0
CPn0446CP0307pmp8	-1.08	-1.560771	1.44735	0	0
CPn0188CP0579	-1.07	-1.539037	1.44203	0	0
CPn0234CP0529	-1.02	-1.552397	1.51473	0	0
CPn0139CP0633yqgE	-0.99	-1.436608	1.45327	0	0
CPn0362CP0395rpsD	-0.97	-1.336192	1.37053	0	0
CPn0675CP0072	-0.97	-2.371788	2.43461	0	0
CPn0340CP0417	-0.97	-1.409647	1.44741	0	0
CPn0142	-0.95	-1.683786	1.77344	0	0
CPn0750CP1122tctD	-0.93	-1.270327	1.37073	0	0
CPn0607CP0140glgC	-0.9	-1.315917	1.45638	0	0
CPn0081CP0694rpoB	-0.9	-1.292093	1.43048	0	0
CPn0693	-0.9	-1.213562	1.35439	0	0
CPn0811CP1060lcrH1	-0.89	-1.559742	1.75453	0	0
CPn0369CP0388	-0.89	-1.31879	1.48657	0	0
CPn0189CP0578	-0.88	-1.313065	1.48802	0	0
CPn0331CP0426	-0.88	-1.356796	1.54923	0	0
CPn0720CP0026	-0.87	-1.236616	1.41846	0	0
CPn0929CP0937	-0.87	-1.333477	1.53319	0	0
CPn0180CP0588	-0.86	-1.533932	1.78193	0	0
CPn0706CP0040	-0.85	-1.197193	1.41656	0	0
CPn0415CP0339	-0.84	-1.157501	1.37407	0	0
CPn0213CP0552	-0.84	-1.920679	2.28061	0	0
CPn0984CP0872nrdA	-0.83	-1.204116	1.44723	0	0
CPn0705CP0041	-0.83	-1.239431	1.49109	0	0
CPn0494CP0260	-0.82	-1.182425	1.43349	0	0
CPn0589CP0159	-0.82	-1.138338	1.3851	0	0
CPn0724CP0022	-0.79	-1.126833	1.41776	0	0
CPn0707CP0039yscN	-0.79	-1.109827	1.40015	0	0
CPn0444CP0309pmp6	-0.79	-1.107815	1.40572	0	0
Cpn0016_pmp41	-0.77	-1.065484	1.37767	0	0
CPn0325CP0432sycE	-0.76	-1.122139	1.47272	0	0
CPn0487CP0267	-0.76	-1.767174	2.33688	0	0
CPn1006CP0847	-0.75	-1.26499	1.68114	0	0
CPn0555CP0197tsp	-0.75	-1.272267	1.70519	0	0
CPn1057CP0793	-0.75	-1.224623	1.64346	0	0
CPN03681_CP03891	-0.74	-1.058391	1.42106	0	0
CPn0610CP0137rho	-0.74	-1.051564	1.41409	0	0
CPn1025CP0827pgi	-0.74	-1.030136	1.39711	0	0
CPn0930CP0935	-0.73	-1.03011	1.40828	0	0
CPn0070	-0.72	-1.656439	2.30784	0	0
CPn0794CP1077	-0.71	-0.959308	1.36026	0.422768	0
CPn0629CP0118secY	-0.7	-1.006018	1.4305	0.422768	0
CPn0678	-0.7	-1.108151	1.57598	0.422768	0
CPn0584CP0164atoS	-0.7	-0.934004	1.32978	0.422768	0
CPn1011	-0.7	-1.009426	1.45139	0.422768	0
CPn0777CP1095groEL2	-0.69	-0.971058	1.39938	0.422768	0
CPn0633CP0114rl6	-0.69	-0.985366	1.42091	0.422768	0
CPn0318CP0439rbfA	-0.69	-0.924927	1.34528	0.422768	0
CPn0631CP0116rs5	-0.68	-0.992021	1.45176	0.422768	0
CPn0860CP1009fliF	-0.68	-0.985875	1.44511	0.422768	0
CPn0220CP0545	-0.68	-0.960318	1.41247	0.422768	0
CPn0291CP0467incB	-0.68	-1.028692	1.51633	0.422768	0
CPn0857CP1012	-0.68	-0.992924	1.4662	0.422768	0
Cpn0548_CP0204	-0.68	-1.137401	1.68467	0.422768	0
Cpn0017_pmp42	-0.68	-1.075115	1.59249	0.422768	0
CPn0443CP0310	-0.67	-0.942922	1.40366	0.422768	0
CPn0688CP0058	-0.66	-0.981667	1.48021	0.422768	0
CPn0467CP0285pmp16	-0.66	-0.952968	1.44192	0.422768	0
CPn0712CP0034	-0.66	-0.94976	1.44902	0.422768	0
CPn0559CP0191	-0.65	-0.902362	1.38286	0.422768	0

CPn0307CP0451glgP	-0.64	-0.914538	1.42306	0.422768	0
Cpn0090_CP0684	-0.64	-0.91098	1.41868	0.422768	0
CPn1033CP0819	-0.64	-0.877373	1.36737	0.422768	0
CPn0063CP0711	-0.64	-0.995832	1.55765	0.422768	0
CPn0453CP0299pmp13	-0.63	-0.891701	1.40593	0.422768	0
CPn0493CP0261	-0.63	-0.932416	1.47026	0.422768	0
CPn0998CP0857ftsH	-0.63	-0.88202	1.40722	0.618377	0
CPn0704fliN	-0.62	-0.869499	1.40377	0.618377	0
CPn0116CP0657rs16	-0.62	-0.872623	1.41183	0.618377	0
CPn0630CP0117rl15	-0.61	-0.858128	1.40111	0.618377	0
CPn0769CP1103topA	-0.61	-0.878944	1.43668	0.618377	0
CPn0702CP0044yscC	-0.61	-0.864755	1.41489	0.618377	0
CPn0670rsbW	-0.61	-0.864252	1.4207	0.618377	0
Cpn0018_pmp51	-0.61	-0.881048	1.45345	0.618377	0
CPn0440CP0313	-0.61	-0.84902	1.40124	0.618377	0
CPn0649CP0098fmt	-0.6	-0.876022	1.45612	0.618377	0
CPn0966CP0894pcnB2	-0.6	-1.03108	1.71656	0.618377	0
CPn0204CP0563	-0.59	-0.942405	1.59346	0.6829329	0.17819
CPn0824CP1047yscS	-0.59	-0.832795	1.41086	0.6829329	0.20904
CPn0738CP0007	-0.59	-1.074531	1.82671	0.6829329	0.26524
CPn1060CP0790tktB2	-0.59	-0.844346	1.4405	0.6829329	0.32427
CPn0096CP0678uvrA	-0.58	-0.808054	1.38481	0.6829329	0.4011
CPn0634CP0113rs8	-0.58	-0.822643	1.41143	0.6829329	0.42098
CPn0826CP1045yscL	-0.58	-0.768198	1.33492	0.6829329	0.652
CPn0161CP0610	-0.57	-0.773381	1.34736	0.6829329	0.70053
CPn0326CP0431malQ	-0.57	-1.359162	2.37146	0.6829329	0.72947
CPn0572CP0177	-0.57	-0.910058	1.58988	0.6829329	0.75399
CPn0934CP0927rnpA	-0.57	-0.862314	1.50693	0.6829329	0.75995
CPn0632CP0115rl18	-0.57	-0.789672	1.39162	0.8286252	0.92756
CPn0459CP0293	-0.57	-1.297267	2.28965	0.8286252	0.95909
CPn0985CP0871nrdB	-0.56	-0.809466	1.43736	0.8286252	1.08602
CPn0557CP0195omcB	-0.56	-0.930708	1.65767	0.8286252	1.15117
CPn0237CP0525yggF	-0.56	-0.79473	1.41721	0.8286252	1.17775
CPn0841CP1028secA2	-0.56	-0.788947	1.41254	0.8286252	1.26607
CPn0801CP1070	-0.56	-0.799158	1.43795	0.8286252	1.37834
CPn0140CP0632yqdE	-0.55	-0.867651	1.57309	0.8286252	1.55546
CPn0535CP0217lspA	-0.55	-0.772661	1.40638	0.8286252	1.64967
CPn0669CP0078	-0.55	-0.752073	1.37746	0.9331365	1.80307
CPn0567CP0182cdsA	-0.54	-0.74365	1.37356	0.9331365	2.01793
CPn0716CP0030	-0.54	-0.803189	1.48411	0.9331365	2.0281
CPn0324CP0433lcrE	-0.54	-0.806572	1.49185	0.9331365	2.05418
CPn0587CP0161yvyD	-0.54	-0.739256	1.36749	0.9331365	2.057
Cpn0015_pmp32	-0.54	-0.761361	1.41358	0.9331365	2.15435
CPn0568CP0181	-0.54	-0.761222	1.41525	0.9331365	2.19073
CPn0395CP0360	-0.54	-0.761134	1.4154	0.9331365	2.19669
CPn0700CP0046	-0.54	-0.738112	1.37373	0.9331365	2.21911
CPn0742CP0003	-0.53	-0.740536	1.40215	0.9331365	2.69986
CPn1031CP0821arcD	-0.53	-1.010929	1.92463	1.0357815	2.86042
CPn0726	-0.52	-0.753928	1.44154	1.0357815	2.98937
CPn0701CP0045karG	-0.52	-0.761512	1.46099	1.0357815	3.09238
CPn0731CP0015	-0.52	-1.235302	2.37738	1.0357815	3.18844
CPn0203CP0564	-0.52	-0.82693	1.59152	1.0357815	3.18974
CPn0236CP0526pyrG	-0.52	-0.727776	1.40294	1.0357815	3.23973
CPn1049CP0803lysC	-0.52	-0.704355	1.3592	1.0357815	3.27207
CPn0956CP0904	-0.52	-0.729537	1.40819	1.0357815	3.28096
CPn0848CP1021tigA	-0.52	-0.722455	1.39608	1.0357815	3.31603
CPn0195CP0572oppA1	-0.52	-0.752674	1.45478	1.0357815	3.32251
CPn0115CP0658ffh	-0.51	-0.704845	1.37454	1.0357815	3.60832
CPn0809CP1062	-0.51	-0.770041	1.50977	1.0357815	3.78497
CPn0122CP0651metG	-0.51	-0.711106	1.39653	1.0357815	3.84027
CPn0365CP0392	-0.51	-0.720173	1.41679	1.0357815	3.89828
CPn0267CP0493	-0.51	-1.219441	2.40829	1.0357815	4.02925
CPn0445CP0308pmp7	-0.5	-0.727891	1.44499	1.0519656	4.20743

CPn0817CP1054gspF	-0.5	-0.702026	1.39634	1.0519656	4.27468
CPn0317CP0440infB	-0.5	-0.71509	1.42273	1.0519656	4.2848
CPn0711CP0035	-0.5	-0.718681	1.43033	1.0519656	4.29575
CPn0650CP0097lpxA	-0.5	-0.703698	1.40131	1.0519656	4.31585
CPn0084CP0691	-0.5	-0.736429	1.46703	1.0519656	4.32891
CPn0708CP0038	-0.5	-0.733612	1.46328	1.0519656	4.37361
CPn0635CP0112rl5	-0.49	-0.672628	1.36072	1.0519656	4.88339
CPn0655CP0092dnaQ2	-0.49	-0.706924	1.43137	1.0519656	4.91623
Cpn0014_pmp31	-0.49	-0.682138	1.38943	1.0519656	5.13883
CPn0849CP1020	-0.49	-0.76005	1.54907	1.0519656	5.16188
CPn0385CP0370pepA	-0.49	-0.672941	1.37715	1.0519656	5.31762
CPn0611CP0136yacE	-0.49	-0.668345	1.37286	1.0519656	5.46122
CPn0879CP0990yycJ	-0.49	-0.678573	1.39518	1.0519656	5.49766
CPn0818CP1053	-0.48	-0.681095	1.40696	1.0781369	5.68143
CPn0894CP0972amn	-0.48	-0.679188	1.40974	1.0781369	5.87079
CPn0419CP0335pbp3	-0.48	-0.710881	1.48146	1.0781369	6.03193
CPn0138CP0634hemL	-0.48	-0.66578	1.39468	1.0781369	6.24271
CPn0791CP1081	-0.47	-0.747353	1.58907	1.0781369	6.86552
CPn0718CP0028	-0.47	-0.67635	1.43915	1.0781369	6.8966
CPn0583CP0165	-0.47	-0.673269	1.43692	1.0781369	7.02583
CPn0391CP0364	-0.47	-1.135828	2.42712	1.1508684	7.07888
CPn0283	-0.47	-0.984718	2.10764	1.1508684	7.1492
CPn0651CP0096fabZ	-0.47	-0.630773	1.35295	1.1508684	7.24151
CPn0229CP0535	-0.47	-0.714142	1.53305	1.1508684	7.27811
CPn0913CP0953	-0.46	-0.631604	1.36212	1.1508684	7.47988
CPn0550fusA	-0.46	-0.637122	1.37451	1.1508684	7.49558
CPn0466CP0286pmp15	-0.46	-0.679507	1.47351	1.1508684	7.72439
CPn0129CP0642	-0.46	-0.676821	1.46921	1.1508684	7.7706
CPn1032CP0820	-0.46	-0.690141	1.50167	1.1508684	7.87702
CPn0870CP0999serS	-0.45	-0.664613	1.46379	1.1508684	8.43182
CPn0196CP0571oppA2	-0.45	-0.651462	1.44542	1.3321949	8.7753
CPn0169CP0602	-0.45	-0.665774	1.47966	1.3321949	8.85454
CPn0504CP0249vacB	-0.45	-0.618156	1.37826	1.3321949	9.00701
CPn0127CP0645ytfF	-0.45	-0.632609	1.41434	1.3321949	9.13727
CPn0165CP0606	-0.45	-0.641551	1.4365	1.3321949	9.20931
CPn0292CP0466incC	-0.44	-0.609553	1.37552	1.3321949	9.58585
CPn0072CP0703	-0.44	-0.615042	1.38943	1.3321949	9.63935

ated Miss rates for Delta=0.27557594‍31

Quantiles	Cutpoint	Miss Rate(%)
0 -> 0.05	-0.434 ->	88.98
0.05 -> 0.1	-0.38 -> -	81.84
0.1 -> 0.15	-0.322 ->	66.13
0.15 -> 0.2	-0.259 ->	63.77
0.2 -> 0.25	-0.215 ->	51.26
0.25 -> 0.7!	-0.172 ->	11.8
0.75 -> 0.8	0.215 ->	67.82
0.8 -> 0.85	0.253 ->	75.38
0.85 -> 0.9	0.293 ->	77.22
0.9 -> 0.95	0.349 ->	84.1
0.95 -> 1	0.412 ->	89.54
