# Supplementary material for: Gene Expression Profiles of Chlamydophila pneumoniae during the Developmental Cycle and Iron Depletion–Mediated Persistence
Source: PLoS Pathog. 2007 Jun 22;3(6):e83. doi: 10.1371/journal.ppat.0030083 (PMC1894823; doi:10.1371/journal.ppat.0030083)
Supplement: Supplementary file 19 [file ppat.0030083.st008.pdf]

Mäurer et. al 2007

Table S8 – LACK Calculations for Upregulated Genes

Supplementary Table 8

Cluster	SGL	Hits	Size	Hits		p-value
12	42	3	754	97	(P>=3)	=0.92
11	48	0	754	97	(P>=0)	=1.00
10	80	1	754	97	(P>=1)	=0.99
9	50	7	754	97	(P>=7)	=0.47
8	86	4	754	97	(P>=4)	=0.99
7	34	1	754	97	(P>=1)	=0.99
6	55	2	754	97	(P>=2)	=0.99
5	65	3	754	97	(P>=3)	=0.99
4	64	2	754	97	(P>=2)	=0.99
3	53	17	754	97	(P>=17)	<0.01
2	142	54	754	97	(P>=54)	<0.01
1	34	2	754	97	(P>=2)	=0.94
