# Supplementary material for: Gene Expression Profiles of Chlamydophila pneumoniae during the Developmental Cycle and Iron Depletion–Mediated Persistence
Source: PLoS Pathog. 2007 Jun 22;3(6):e83. doi: 10.1371/journal.ppat.0030083 (PMC1894823; doi:10.1371/journal.ppat.0030083)
Supplement: Supplementary file 20 [file ppat.0030083.st009.pdf]

Mäurer et. al 2007

Table S9 – LACK Calculations Downregulated Genes

Supplementary Table 9

Cluster	SGL	Hits	Size	Hits		p-value
12	42	19	754	104	(P>=19)	<0.01
11	48	31	754	104	(P>=31)	<0.01
10	80	21	754	104	(P>=24)	<0.01
9	50	0	754	104	(P>=0)	=1.00
8	86	12	754	104	(P>=12)	=0.53
7	34	3	754	104	(P>=3)	=0.87
6	55	3	754	104	(P>=3)	=0.98
5	65	6	754	104	(P>=6)	=0.90
4	64	3	754	104	(P>=3)	=0.99
3	53	1	754	104	(P>=1)	=0.99
2	142	1	754	104	(P>=1)	=0.99
1	34	1	754	104	(P>=1)	=0.99
