# Supplementary material for: Gene Expression Profiles of Chlamydophila pneumoniae during the Developmental Cycle and Iron Depletion–Mediated Persistence
Source: PLoS Pathog. 2007 Jun 22;3(6):e83. doi: 10.1371/journal.ppat.0030083 (PMC1894823; doi:10.1371/journal.ppat.0030083)
Supplement: Supplementary file 21 [file ppat.0030083.st010.pdf]

Mäurer et. al 2007

Table S10 – Real-Time PCR Primers Used in This Study

Supplementary Table 10 - Primers used in this study

Name	Cpn#	Forward	Reverse
lrcH1	*811*	CTCAAGGGAAGTACAACGAAGC	ATTGTTAGATTCTTCGGGTTGC
yscC	*702*	AAGAAGTCGAAGAGCCTGTACC	TCTCTGTAACTGTGGAGGTTTGC
sctS	*824*	ACCTCCCATCATCTTAGCTTCC	CGACTAGTTTGACTGCAAAAGC
sctN	*707*	AAAGCCGTAGTTCCTAATGTGC	TTCCTGCTCGAATGTGTAAGG
pmp18	*471*	GCCAGCTTTATGGTGATTGG	TTTTGATGCACGAAGAGTCG
groEL3	*898*	CCTTAAAGACGCGACAATTAGC	AATCTTAAGAGCGAGGGTATATAAAGG
16S	*16S*	CCAGTTCGGATTGTAGTCTGC	AGTCATCAGCCTCACCTTGG
minD	*805*	GTTTCATCCAAGGAATTTCAGC	ACAGGTTTGCCATGAATCG
murB	*988*	AGGAGGCTCGTGAAGTTATCC	CTAAAGCTGCAAAGGAAAGACC
cdp/ftsK	*880*	CTTCCTAAGCCTTCGGTTCC	AGAACCGGGGATCTCTTCC
rs2	*696*	CGTCGATACTAACTGCGATCC	CGTCAGATTCGTCATCTTGG
tufA	*74*	GAAGGCGGACGTCATAAGC	GACCACCTTCACGAATTGC
L29	*639*	AGCGACGACGATTTAGATGC	TATGAACTTTCACAACTTTGTTTTGC
ABCT	*691*	GGTATCTTCGGGTGAGATTGC	CTCTGGAGGCATTTGAAAACC
htrA	*979*	CCTTCGGTCTTCAAGCTACG	AGGCTAGGAATCGCAAACC
nth	*837*	ACACTCAACGCGCTATTTCC	AAAATAGATTGGGCGTCTGG
secA2	*841*	CGCCATGATGTCTTACATGC	CAGCAGCGATCTTTTCAGC
gyrB2	*715*	ATGTAAAGGAAGCGATTGTACAGG	CCCTTCGGTAAGGAAAATCG
secA1	*260*	ACAGAAAGCGGCTGTTGG	ATATGCGAAAATCTTCTTGTGTAGG
gyrB1	*275*	TTCTACCGCGCTTACTAGGG	GTTTGCCCTTCAAATTGTGG
hctA	*886*	GATAGCATCCAACACGACTTAGC	GCAGGAGCCTTAGTTGATGG
hctB	*384*	AAACCTGCAGTTCGTAAGACG	GCTTTCTTAGGAGAACCTTTAGCC
omcB	*557*	GCGAAGCTGAATTCGTAAGC	TTGACCGCATTTAGTATAAGAACG
xseA	*1062*	TTTGGTTTACGCTGGAGAGG	AGTTCCCTTGGACAGTGACG
sctS	*824*	ACCTCCCATCATCTTAGCTTCC	CGACTAGTTTGACTGCAAAAGC
sycE	*325*	TTTAATCCGTTTCAGCGATACC	GTTGAGTGACCTCACCGTAGC
rpsD	*362*	CTCAATATTTCGCAACAAGAGC	AAGTTCCTGAATCGCATTGG
rpoD	*756*	TCTCTACAAGCCGAGGTTGG	AAACAAATCGCTCACGATCC
rpoN	*771*	TCCCTCTAAAGCACCTCTTCC	TTAGTTGTGCACGGTATTTTGC
ftsW	903	CAGCTAACGATTCAACCTTCG	GTCATGATAAACACAGGAATCAGG
ltuB	*333*	AGAAGAGCAGAACGGATAGGG	AAGAGAGAGCGAACTAACTGATCG
atoC/ctcB	*586*	GACGACATCCTCCCTCTGG	AGCATGTCTTCGGTGAGTAGG
noN	*189*	CATCTCGTCTCCTGAAGTGG	GGGAAGGGGTGTACTGTTGG
noN	*1006*	TGCTGGAGCAAAAGAAGACG	CTTGTGAGGATTGCTGTTGG
noN	*720*	ACCGCCCTGAAGAAGTCC	CCTTACATTGTTCCTGCTTGC
noN	*676*	ATCGGGTATCGGAAAGTGC	CTCCCTGTTCTTTTGGATGG
noN	*667*	GGGTACGCGAAAATCATCC	GTCATTGTATTCGCCCAAGC
noN	*677*	AGGCGATAGCTCCAGTACCC	AAGCACCCTGAGAAGAATCG
topA	*769*	GAGAAAGCCCGTCACTATGC	GGAATCCGTACGCATGTAGG
mdhC	*1028*	ATTTCCATGCGATGTTACGC	TCCAACCAATCTCGATCTCC
oppC1	*200*	CGAAGCCTTCATTAGCTTCC	ATCCATGAGATCCCTCTTCG
yfhC	*1001*	CTTCAAAGAAGCGCGTAAGG	AGGACGGTATCCAACAAACG
surE	*262*	CAAGTAGTCTGCGCCTCTCC	ATCGACCAAGGCTTGTTTTG
noN	*1070*	GACTGGAGGTCGTGAAATGG	ACCAGGACAATGCTCTCTGC
lepB	110	AGCTATGCCGATCTTTTTGG	GAGGCTTTGGGTAGGAAAGG
ribA/ribB	872	GGACACTTTTTCCCCCTAGC	GAGCAATGATCGATGTCACG
lspA	*535*	GCGGATATTTGTGATTCTTGG	GAAATCAATACATCGGCAACG
dnaB	616	CAATTACTTTCCGGCTCAGG	ACATCACTAAATCCGAATCTTGC
dcrA	55	TTCGAGATCTCTGGGTCTTCC	AACCCCGTAGTTCCCTTCTC
ide/ptr	957	GAATCTTCCCATCCGATTCC	TATCGCCTTTCTCCGTAAGC
