# Supplementary material for: Gene Expression Profiles of Chlamydophila pneumoniae during the Developmental Cycle and Iron Depletion–Mediated Persistence
Source: PLoS Pathog. 2007 Jun 22;3(6):e83. doi: 10.1371/journal.ppat.0030083 (PMC1894823; doi:10.1371/journal.ppat.0030083)
Supplement: Supplementary file 22 [file ppat.0030083.st011.pdf]

Mäurer et. al 2007

Table S11 – Determination and Stability of Housekeeping Gene Candidates together with Randomly Picked Control Genes

Supplementary Table 11

Rank	Gene name	M-value
	lcrH1	1.76
	yscC	1.88
	sctS	0.92
	sycE	1.94
	sctN	1.6
#1	*pmp18*	1.53
#2	*groEL*	1.45
#3	*16S*	1.19
#4	*minD*	1.11
#5	*murB*	1.04
#6	*cdp*	0.86
#7	*rs2*	0.78
#8	*tufA*	0.71
#9	*L29*	0.59
#10	*ABCT*	0.59
