# Supplementary material for: Gene Expression Profiles of Chlamydophila pneumoniae during the Developmental Cycle and Iron Depletion–Mediated Persistence
Source: PLoS Pathog. 2007 Jun 22;3(6):e83. doi: 10.1371/journal.ppat.0030083 (PMC1894823; doi:10.1371/journal.ppat.0030083)
Supplement: Supplementary file 23 [file ppat.0030083.st012.pdf]

Mäurer et. al 2007

Table S12 – qRT-PCR Data for Different Time Points of the Acute and Iron Depletion–Mediated Persistence

SupplementaryTable 12 - qRTPCR data

Legend: noN=NoName
n.A.=experiment not performed
noS=noSignal (experiment performed but not qRTPCR signal at this timepoint)

Cluster	Cpn#	Gene	qRTPCR 16S 6hpi Cpn+	SD DAM-	qRTPCR HKI 6hpi Cpn+	SD DAM-	Array 6hpi Cpn+ DAM-
noC	*841*	*secA2*	-5.33	5.48	-1.15	1.84	-2.44
noC	*1062*	*xseA*	-1.03	2.26	3.18	0.65	-2.73
12	*333*	*ltuB*	1.49	0.09	1.76	0.55	1.80
12	*586*	*atoC/ctcB*	3.83	0.73	2.77	1.75	1.46
12	*189*	*noN*	4.92	0.30	5.13	1.67	2.82
12	*1006*	*noN*	9.83	2.36	17.47	1.94	4.13
12	*720*	*noN*	-1.02	1.80	1.34	0.31	-1.29
12	*676*	*noN*	1.39	0.27	1.55	0.56	1.05
12	*667*	*noN*	-2.62	1.81	1.18	1.52	1.30
11	*384*	*hctB*	-3.48	0.66	-3.32	0.34	-10.90
11	*557*	*omcB*	-2.91	1.57	-2.14	0.26	-7.38
10	*886*	*hctA*	-148.00	1.30	-85.40	1.27	-10.91
10	*769*	*topA*	-6.58	2.06	-6.98	1.66	-1.68
10	*1028*	*mdhC*	-5.72	2.56	-4.42	1.34	-3.78
10	*200*	*oppC1*	noS	noS	noS	noS	-1.18
10	*824*	*sctS*	-1.97	0.38	1.03	0.03	-1.23
9	*1001*	*yfhC*	-2.96	0.47	-1.55	0.45	-1.40
9	*262*	*surE*	-3.78	0.24	-1.45	0.41	-2.38
9	*1070*	*noN*	1.06	2.74	-1.66	0.08	-1.09
9	*872*	*ribA/ribB*	-1.15	0.03	3.68	0.03	-6.55
8	*260*	*secA1*	1.05	2.60	-3.41	0.14	-4.39
8	*535*	*lspA*	noS	noS	noS	noS	-1.80
8	*616*	*dnaB*	noS	noS	noS	noS	-3.52
7	*275*	*gyrB1*	1.98	2.32	-3.42	0.88	-4.00
6	*903*	*ftsW*	1.28	0.37	1.46	0.13	-1.10
6	*880*	*ftsK*	1.72	0.10	1.58	0.48	-1.34
4	*55*	*dcrA*	1.66	0.36	1.70	0.04	1.47
3	*979*	*htrA*	-6.23	3.33	-1.67	0.02	-1.12
2	*837*	*nth*	5.13	3.01	13.12	0.86	10.80
2	*715*	*gyrB2*	73.02	1.79	15.55	0.63	10.90
1	*957*	*ide/ptr*	4.80	1.37	7.67	0.57	1.78

Cluster	Cpn#	Gene	qRTPCR 16S 12hpi Cpn+	SD DAM-	qRTPCR HKI 12hpi Cpn+	SD DAM-	Array 12hpi Cpn+ DAM-
noC	*841*	*secA2*	1.61	3.34	-1.22	0.05	-1.19
noC	*1062*	*xseA*	4.81	3.34	2.52	0.91	-1.65
11	*384*	*hctB*	-5.76	1.74	-14.85	6.55	-47.46
11	*557*	*omcB*	-2.96	3.14	-6.46	4.05	-19.60
8	*260*	*secA1*	2.54	0.55	1.53	0.18	-1.33
7	*275*	*gyrB1*	2.17	0.54	-1.22	1.53	1.20
3	*979*	*htrA*	-1.29	0.84	-1.67	0.32	-1.19
2	*837*	*nth*	15.13	11.39	6.98	1.73	7.76
2	*715*	*gyrB2*	12.61	0.58	5.31	1.09	8.00

Cluster	Cpn#	Gene	qRTPCR 16S 18hpi Cpn+	SD DAM-	qRTPCR HKI 18hpi Cpn+	SD DAM-	Array 18hpi Cpn+ DAM-
noC	*841*	*secA2*	1.63	2.08	-1.26	0.01	-1.04
12	*333*	*ltuB*	-7.40	1.32	-10.73	2.08	-2.69
12	*586*	*atoC/ctcB*	-1.98	1.53	-1.03	1.38	-1.02
12	*189*	*noN*	noS	noS	noS	noS	-3.45
12	*1006*	*noN*	1.28	1.98	-2.63	1.76	-2.20
12	*720*	*noN*	-1.69	1.29	-2.26	1.80	-2.69
12	*676*	*noN*	-5.97	1.84	-8.57	1.03	-4.17
12	*667*	*noN*	-368.47	1.14	-175.45	3.08	-1.97
11	*811*	*lcrH1*	-17.68	7.25	-16.81	7.23	-72.20
11	*384*	*hctB*	-21.99	1.44	-34.07	3.19	-69.00
11	*677*	*noN*	-3.08	1.54	-2.60	1.35	-10.40
10	*325*	*sycE*	-13.07	6.81	-11.94	6.29	-10.91
10	*824*	*sctS*	-3.02	0.82	-2.86	0.84	-1.93
10	*886*	*hctA*	-450.16	1.48	-681.14	1.07	-139.00
10	*769*	*topA*	-1.02	1.55	-1.40	1.16	-1.82
10	*1028*	*mdhC*	-10.14	2.29	-12.05	3.51	-4.13
10	*200*	*oppC1*	-3.97	1.26	-2.89	1.03	-1.55
10	*824*	*sctS*	-2.00	1.18	-2.20	1.72	-1.93
9	*1001*	*yfhC*	1.15	1.41	-1.57	1.10	1.10
9	*262*	*surE*	-2.75	1.45	-1.86	1.16	-1.87
9	*1070*	*noN*	-2.23	1.74	1.25	1.33	-1.10
9	*110*	*lepB*	-4.80	1.72	-5.08	1.87	-1.28
9	*872*	*ribA/ribB*	-1.45	1.03	-1.72	1.62	-1.47
8	*707*	*sctN/yscN*	-5.57	0.54	-5.22	0.67	-1.75
8	*260*	*secA1*	-1.71	2.25	-1.51	0.13	-2.29
8	*702*	*yscC*	-3.57	1.26	-3.38	1.27	-4.17
8	*535*	*lspA*	-2.00	1.18	1.63	1.09	-2.77
8	*616*	*dnaB*	-1.57	1.48	-2.02	2.19	1.22
6	*903*	*ftsW*	1.18	1.03	-1.05	n.A.	1.08
6	*880*	*ftsK*	-1.02	3.70	1.06	2.14	1.17
4	*55*	*dcrA*	1.58	1.06	1.72	1.14	1.64
3	*979*	*htrA*	-1.33	1.71	-1.34	0.21	2.41
2	*837*	*nth*	2.24	0.35	2.32	1.11	3.70
2	*715*	*gyrB2*	1.59	0.12	1.15	0.08	3.15
1	*957*	*ide/ptr*	1.43	0.97	1.20	1.16	1.37

Cluster	Cpn#	Gene	qRTPCR 16S 24hpi Cpn+	SD DAM-	qRTPCR HKI 24hpi Cpn+	SD DAM-	Array 24hpi Cpn+ DAM-
noC	*841*	*secA2*	1.44	1.47	-1.25	0.10	-1.03
noC	*1062*	*xseA*	-1.41	1.52	-1.44	0.06	1.13
11	*811*	*lcrH1*	-9.60	2.53	-18.13	1.77	-11.10
10	*325*	*sycE*	-8.43	1.41	-17.15	5.63	-9.11
10	*824*	*sctS*	-2.99	0.89	-6.20	2.76	-1.80
8	*707*	*sctN/yscN*	-2.37	0.34	-4.57	0.12	-2.77
8	*260*	*secA1*	-1.43	1.75	-1.41	0.15	-1.61
8	*702*	*yscC*	-2.91	0.72	-5.99	2.40	-4.52
3	*979*	*htrA*	-1.66	1.82	-1.60	0.18	1.03

2	*837*	*nth*	1.62	0.46	1.43	0.18	2.36
2	*715*	*gyrB2*	1.46	0.02	-1.04	0.03	2.53

Cluster	Cpn#	Gene	***qRTPCR 16S* 60hpi Cpn+**	**SD DAM-**	***qRTPCR HKI* 60hpi Cpn+**	**SD DAM-**	***Array* 60hpi Cpn+ DAM-**
12	*333*	*ltuB*	5.08	1.30	8.49	1.30	8.75
12	*586*	*CtcB/atoC*	2.09	0.39	2.86	0.15	2.20
12	*189*	*noN*	1.06	2.41	1.25	0.81	1.46
12	*1006*	*noN*	-1.33	0.13	1.05	0.37	2.66
12	*720*	*noN*	1.82	0.29	3.39	0.02	1.39
12	*676*	*noN*	1.21	0.27	1.69	0.82	3.56
12	*667*	*noN*	2.62	0.49	3.02	0.51	2.50
11	*811*	*lcrH1*	2.43	0.74	4.11	1.06	3.38
11	*384*	*hctB*	3.16	0.50	5.28	0.86	1.93
10	*325*	*sycE*	-1.62	0.76	1.26	0.59	-1.33
10	*824*	*sctS*	-2.32	0.15	-1.36	0.15	-1.39
10	*886*	*hctA*	1.58	0.01	2.05	1.15	2.30
10	*769*	*topA*	0.99	0.37	-1.04	0.00	1.08
10	*1028*	*mdhC*	1.20	0.32	-1.04	0.25	1.11
10	*824*	*sctS*	1.15	0.09	1.11	1.62	-1.33
9	*1001*	*yfhC*	0.92	0.48	1.02	1.42	1.30
9	*262*	*surE*	1.20	3.04	-1.08	2.02	1.28
9	*1070*	*noN*	1.35	1.64	-1.23	0.21	1.17
9	*872*	*ribA/ribB*	1.39	0.53	-1.23	0.18	1.00
8	*707*	*sctN/yscN*	-1.18	1.38	1.55	0.40	-1.39
8	*702*	*yscC*	-1.04	0.05	1.64	0.00	1.75
6	*903*	*ftsW*	-1.82	1.29	-1.50	0.16	-1.16
6	*880*	*ftsK*	-3.39	0.72	0.82	-1.23	-1.12
4	*55*	*dcrA*	-1.79	0.27	-1.97	0.11	-1.50
1	*957*	*ide/ptr*	-1.30	0.08	1.03	0.04	1.05

Cluster	Cpn#	Gene	**qRTPCR 16S 72hpi Cpn+**	**SD DAM-**	**qRTPCR HKI 72hpi Cpn+**	**SD DAM-**	**Array 72hpi Cpn+ DAM-**
noC	*841*	*secA2*	-1.72	0.18	-1.18	0.11	-1.51
noC	*1062*	*xseA*	-1.14	0.13	1.86	0.56	-1.58
12	*333*	*ltuB*	25.81	3.74	33.69	1.26	8.33
12	*586*	*atoC/ctcB*	4.28	0.31	6.64	1.15	1.61
12	*189*	*noN*	3.29	0.48	4.17	1.50	1.76
12	*1006*	*noN*	11.40	1.27	10.54	2.12	5.75
12	*720*	*noN*	6.56	0.77	5.62	1.91	4.17
12	*676*	*noN*	10.36	1.43	10.11	2.62	10.50
12	*667*	*noN*	19.12	1.44	33.32	11.48	2.74
11	*811*	*lcrH1*	12.42	0.99	17.69	1.24	9.48
11	*384*	*hctB*	4.95	0.15	5.10	2.33	2.85
11	*677*	*noN*	6.96	0.16	17.80	5.36	10.80
10	*325*	*sycE*	-1.66	0.68	-1.12	0.61	-1.19
10	*824*	*sctS*	1.07	0.48	1.57	0.54	-1.30
10	*886*	*hctA*	1.60	0.19	1.48	0.67	2.71
10	*769*	*topA*	-1.02	0.36	1.22	0.67	1.00
10	*1028*	*mdhC*	1.03	0.26	1.05	0.47	1.17

10	*200*	*oppC1*	1.15	0.49	1.40	0.42	1.12
10	*824*	*sctS*	1.33	0.43	1.63	1.20	-1.30
9	*1001*	*yfhC*	1.44	0.01	1.22	0.96	1.28
9	*262*	*surE*	1.46	0.07	1.52	0.33	1.16
9	*1070*	*noN*	1.08	0.38	1.35	0.88	1.04
9	*110*	*lepB*	-1.04	0.19	1.42	0.19	1.02
9	*872*	*ribA/ribB*	-1.11	0.21	-1.02	0.22	-1.49
8	*707*	*sctN/yscN*	-1.36	0.02	1.04	0.05	-1.18
8	*260*	*secA1*	-1.23	0.22	1.66	0.03	-1.29
8	*702*	*yscC*	1.11	1.43	1.60	0.50	1.25
8	*535*	*lspA*	2.16	0.33	1.82	1.54	-1.24
8	*616*	*dnaB*	-1.59	0.28	-1.17	0.76	1.06
6	*903*	*ftsW*	-3.34	1.50	-1.28	0.10	-1.85
6	*880*	*ftsK*	-1.18	1.02	1.05	0.14	-1.46
4	*55*	*dcrA*	-1.41	0.63	-1.43	0.24	1.33
3	*979*	*htrA*	-1.46	0.20	1.39	0.08	1.30
2	*837*	*nth*	-1.58	0.44	1.33	0.11	1.28
2	*715*	*gyrB2*	-1.99	0.68	1.01	0.28	1.12
1	*957*	*ide/ptr*	-2.86	0.29	-3.95	0.05	1.00

**Cpn#**	**Name**	**qRTPCR 16S 24hpi DAM+**	**SD**	**qRTPCR HKI 24hpi DAM+**	**SD**	**Array 24hpi DAM+**
362	*rpsD*	1.17	0.35	1.16	0.39	-1.59
325	*sycE*	-1.12	0.04	1.02	0.14	-1.47
707	*sctN*	-1.24	0.36	-1.07	0.58	-1.30
260	*secA1*	1.35	0.10	1.25	0.06	-1.36
811	*lcrH1*	-2.70	0.57	-2.21	0.16	-2.80
979	*htrA*	1.48	0.19	1.31	0.25	-1.28
837	*nth*	2.49	0.27	2.35	0.51	2.22
702	*yscC*	-1.06	0.04	-1.27	0.13	-1.64
384	*hctB*	-8.54	1.16	-7.21	0.80	-11.70
557	*omcB*	-6.67	3.14	-2.86	1.25	-6.35
841	*secA2*	1.01	0.08	-1.14	0.16	1.13
1062	*xseA*	2.17	0.69	1.94	0.73	1.34
715	*gyrB2*	15.36	1.99	5.22	0.26	1.90
756	*rpoD*	-1.72	0.32	-1.75	0.37	-1.47
711	*rpoN*	2.58	0.66	2.33	0.48	1.27
275	*gyrB1*	5.78	5.49	2.21	1.30	1.38
886	*hctA*	-3.49	2.44	-3.56	0.22	-1.67

**CPn#**	**Gene**	**qRTPCR 16S 48hpi DAM+**	**SD**	**qRTPCR HKI 48hpi DAM+**	**SD**	**Array 48hpi DAM+**
362	*rpsD*	-2.56	1.14	-3.38	0.43	-3.97
325	*sycE*	-11.74	2.69	-11.21	2.92	-4.90
707	*sctN*	-5.09	1.36	-6.00	0.30	-3.18
260	*secA1*	3.15	1.56	2.39	1.41	2.48
811	*lcrH1*	-6.54	1.52	-6.09	1.06	-22.40
979	*htrA*	1.89	1.01	1.58	0.35	3.49
837	*nth*	2.68	1.36	2.25	0.44	8.75
702	*yscC*	-1.72	0.49	-2.79	0.22	-5.00

384	*hctB*	-10.85	0.76	-4.72	0.87	-7.81
557	*omcB*	-4.25	1.04	-1.62	0.65	-9.67
841	*secA2*	1.16	3.21	-1.01	1.93	-2.71
1062	*xseA*	3.08	0.19	2.38	1.14	1.80
715	*gyrB2*	9.90	0.82	21.17	4.21	1.52
756	*rpoD*	-1.94	1.16	-2.46	0.74	1.01
711	*rpoN*	3.50	2.97	1.81	1.29	-1.64
275	*gyrB1*	-1.53	3.63	2.71	3.20	-1.10
886	*hctA*	-17.46	15.04	-6.58	5.05	-3.92

**CPn#**	**Gene**	**qRTPCR 16S 72hpi DAM+**	**SD**	**qRTPCR HKI 72hpi DAM+**	**SD**	**Array**
362	*rpsD*	n.A.	n.A.	n.A.	n.A.	n.A.
325	*sycE*	-5.57	3.36	-2.82	1.59	-4.05
707	*sctN*	-8.17	0.42	-4.27	0.03	-2.61
260	*secA1*	3.56	2.06	2.74	0.42	2.18
811	*lcrH1*	-5.69	3.98	-7.36	0.96	-3.74
979	*htrA*	2.76	1.43	2.21	0.15	3.18
837	*nth*	8.76	1.56	6.02	0.60	5.50
702	*yscC*	-2.69	0.55	-1.40	0.21	-2.60
384	*hctB*	n.A.	n.A.	n.A.	n.A.	n.A.
557	*omcB*	n.A.	n.A.	n.A.	n.A.	n.A.
841	*secA2*	1.12	0.22	-1.02	0.34	-2.38
1062	*xseA*	5.15	0.92	4.00	0.54	1.26
715	*gyrB2*	n.A.	n.A.	n.A.	n.A.	n.A.
756	*rpoD*	n.A.	n.A.	n.A.	n.A.	n.A.
711	*rpoN*	n.A.	n.A.	n.A.	n.A.	n.A.
275	*gyrB1*	n.A.	n.A.	n.A.	n.A.	n.A.
886	*hctA*	n.A.	n.A.	n.A.	n.A.	n.A.
